# Bioaccumulation and biomagnification of microplastics in marine organisms: A review and meta-analysis of current data

**DOI:** 10.1371/journal.pone.0240792

**Published:** 2020-10-16

**Authors:** Michaela E. Miller, Mark Hamann, Frederieke J. Kroon

**Affiliations:** 1 Australian Institute of Marine Science (AIMS), Townsville, Queensland, Australia; 2 Division of Research and Innovation, AIMS@JCU, James Cook University, Townsville, Queensland, Australia; 3 College of Science and Engineering, James Cook University, Townsville, Queensland, Australia; VIT University, INDIA

## Abstract

Microplastic (MP) contamination has been well documented across a range of habitats and for a large number of organisms in the marine environment. Consequently, bioaccumulation, and in particular biomagnification of MPs and associated chemical additives, are often inferred to occur in marine food webs. Presented here are the results of a systematic literature review to examine whether current, published findings support the premise that MPs and associated chemical additives bioaccumulate and biomagnify across a general marine food web. First, field and laboratory-derived contamination data on marine species were standardised by sample size from a total of 116 publications. Second, following assignment of each species to one of five main trophic levels, the average uptake of MPs and of associated chemical additives was estimated across all species within each level. These uptake data within and across the five trophic levels were then critically examined for any evidence of bioaccumulation and biomagnification. Findings corroborate previous studies that MP bioaccumulation occurs within each trophic level, while current evidence around bioaccumulation of associated chemical additives is much more ambiguous. In contrast, MP biomagnification across a general marine food web is not supported by current field observations, while results from the few laboratory studies supporting trophic transfer are hampered by using unrealistic exposure conditions. Further, a lack of both field and laboratory data precludes an examination of potential trophic transfer and biomagnification of chemical additives associated with MPs. Combined, these findings indicate that, although bioaccumulation of MPs occurs within trophic levels, no clear sign of MP biomagnification *in situ* was observed at the higher trophic levels. Recommendations for future studies to focus on investigating ingestion, retention and depuration rates for MPs and chemical additives under environmentally realistic conditions, and on examining the potential of multi-level trophic transfer for MPs and chemical additives have been made.

## Introduction

Contamination of the marine environment with microplastics (MPs; plastics < 5 mm) has been identified as an issue of global concern [1], and documented extensively in seawater, marine sediments, and marine biota [2]. Microplastics are of particular concern as a pollutant in environmental systems because their small size and variable buoyancy makes them readily available for uptake by a wide range of organisms across different trophic levels and feeding strategies [3]. Indeed, the uptake of MPs has been confirmed in wild populations of numerous marine organisms across all trophic levels collected from their natural habitat [2]. The prevalence of such reports has resulted in bioaccumulation, and in particular biomagnification of MPs and associated chemical additives, often being inferred in the literature on marine MP contamination [4–6]. However, limited published evidence appears to exist for trophic transfer and biomagnification of MPs and associated additives within food webs in marine environments.

The ecological risks of MP contamination can be defined as the likelihood of adverse ecological effects occurring as a result of exposure to MPs [7, 8]. Marine organisms can be exposed through direct ingestion of MPs, through indirect ingestion of MPs via prey items, or by means of respiration. Irrespective of the pathway, MP intake can result in adverse physical and chemical impacts on marine organisms [9–11]. Examples of potential impacts include physical retention of MPs in digestive tracts [12] and chemical leaching of plastic additives into tissues [13]. These impacts are often investigated during controlled laboratory exposures using a variety of endpoints such as growth rate [14, 15], fecundity [16], and mortality [17]. In wild-caught organisms, however, causality between MP exposure pathways and observed effects is often difficult to ascertain due to the multitude of stressors present in the marine environment [18]. Hence, understanding endpoints such as bioaccumulation and biomagnification can assist in improving our understanding of the potential ecological effects associated with different MP exposure pathways in the marine environment [8].

Bioaccumulation and biomagnification are two critical concepts used in ecological risk assessments to determine the extent of pollutant transport within food webs [19]. The classical concept of bioaccumulation and biomagnification usually refers to dissolved chemical contamination [20], although the terminology has been readily adopted by the MP literature [21–23]. In this study, bioaccumulation (or body burden) is defined as the net uptake of a contaminant (i.e. MPs or additives) from the environment by all possible routes (e.g. contact, ingestion, respiration) from any source (e.g. water, sediment, prey) [24, 25]. In other words, bioaccumulation is occurring when uptake of a contaminant is greater than the ability of an organism to egest a contaminant [26]. Bioaccumulation and subsequent trophic transfer of a contaminant may result in the biomagnification of these contaminants at higher trophic levels [27]. Biomagnification across a food web can thus be defined as the increase in concentration of a contaminant (i.e. MPs or additives) in one organism compared to the concentration in its prey [24, 25]. An important assumption for this definition is that all contamination in higher trophic levels is a direct result of consumption of prey in lower trophic levels, i.e. trophic transfer is occurring.

This study examines whether current, published findings support the premise that MPs, and their chemical additives, bioaccumulate and biomagnify across a general marine food web. First, following a systematic review of the literature, uptake data on MPs and their additives derived from field observations and laboratory experiments were standardised by sample size for individual marine species. For each species, feeding habit was also noted to provide an alternative perspective based on previous findings [28, 29]. Second, following assignment of each species to one of five main trophic levels, the average uptake of MPs and of associated chemical additives was estimated across all species within each level. These uptake data within and across the five trophic levels were then critically examined for any evidence of bioaccumulation and biomagnification. If trophic transfer and biomagnification of MPs and associated additives occurs within marine food webs, an increase in average bioaccumulation from lower to higher trophic levels is expected.

## Materials and methods

### Systematic review procedures

**Fig 1:**
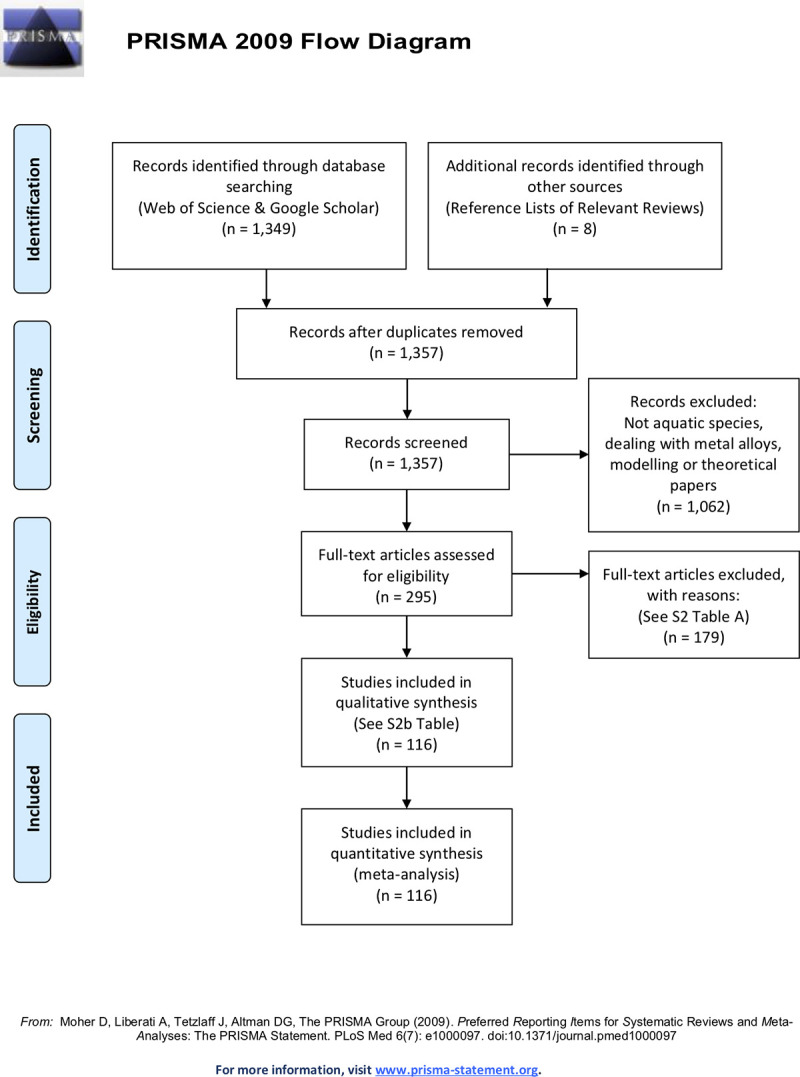
PRISMA flowchart providing the steps of data collection for the systematic review of evidence for bioaccumulation and biomagnification in a general marine food web. The review focussed on microplastics and associated chemical additives detected in marine species from coastal, pelagic, reef and deep-sea environments.

To conduct a systematic review and meta-analyses of the global literature on MP contamination data for individual marine species, an established protocol was followed (PRISMA [30], Fig 1). Specifically, a thorough literature search was conducted to evaluate whether bioaccumulation and biomagnification of MPs and associated additives occurs, either *in situ* or under experimental laboratory settings. The search was performed in Google Scholar and Web of Science^TM^, finalised in July 2019, and covered the years 1969 to 2019. The search included the following terms: microplastics, plastics, ingestion, trophic transfer, toxicity, fish, plastic additives, effects, and impacts. Additional records were also identified through reference lists in various review studies. Following removal of duplicate records, the remaining publications were screened based on study organisms and contaminants of concern (i.e. MPs and/or associated additives). Records that did not examine MPs and/or associated additives in aquatic species from coastal, pelagic, reef and deep-sea environments were subsequently removed. As the focus of this study was on MPs and associated chemical additives rather than environmental contaminants adsorbed to MPs, plastic additives considered here were limited to those outlined in Hahladakis et al. [31] and Hermabessiere et al. [3], and only if directly related to MP contamination. Full text articles were obtained for the remaining records where possible and assessed for eligibility for inclusion in the qualitative and quantitative assessment of evidence for bioaccumulation and biomagnification in a general marine food web. Criteria for exclusion included lack of species-specific information, scientific names not given, inability to assign a trophic level to species, non-aquatic species (i.e. birds), and contaminants were not MPs or associated additives. Lack of polymer assignment of putative MPs with a validated laboratory method, i.e. Fourier Transform Infrared Spectroscopy (FTIR), Raman Spectroscopy, or Gas chromatography–mass spectrometry (GC-MS) [32], was not used as a criterion as this would have excluded too many reports from the review. Finally, while there has been debate over the larger size limit of MPs being either < 1 mm [33] or < 5 mm [34], the more commonly used threshold of < 5 mm has been utilised when including literature [1].

### Standardisation of contamination data

To enable comparison of contamination data in a consistent format, the findings of eligible reports on MP contamination were collated and standardised into number of MPs per individual organism (MPs individual^-1^; i.e. body burden) for each individual species (S1 Fig in S1 File). For articles originally reporting in this unit the contamination data was used as is, while for articles reporting in MP g^-1^, the contamination data was converted to MPs individual^-1^ using the reported individual weights. The unit of MPs individual^-1^ is representative of MPs per number of total organisms in the sample size for a particular species, rather than taken from only the number of organisms that exhibited MP contamination. Moreover, average (± standard deviation, S.D.) MPs individual^-1^ values presented throughout this study include data collated from reports of zero contamination, with concentrations representative of MPs per number of total organisms in the sample size for a particular species, rather than taken from only the number of organisms that exhibited MP contamination. Similarly, concentrations of plastic additives per individual (ng g^-1^; i.e. body burden) were standardised for each individual species and were based on reported and quantified concentrations of additives in the tissues of the target organisms. All supplementary material available was examined if such data on MPs or additives were not reported in the original article. Any contamination data that could not be standardised given the information presented was removed from analysis. This included data presented as a percentage, without quantifying the number of particles extracted from organisms, as well as data lacking a sample size. Finally, to enable consistency in reviewing, terminology for MP shapes reported in the original article was condensed into four categories, namely fibres (alternatively ‘filaments,’ ‘rope’ and ‘fishing line’), fragments (alternatively ‘particle,’ ‘irregular’ and ‘crystal’), films, and spheres (alternatively ‘beads’ and ‘pellets’) [35].

### Assignment to trophic level

To enable comparison of contamination data within and across trophic levels, each individual species was assigned to a specific trophic level using FishBase [36] for all fish species and SeaLifeBase [37] for all other marine species. These databases use recent information on diet composition and food items, combined with modelling, to obtain a numerical trophic level value for individual species. In short, the trophic level of a given species is estimated using the equation:

[TrophicLevel=1+meantrophicleveloffooditems]

including a weighted mean based on the contribution of the various food items to the overall diet of the species [36, 37]. For the purpose of this study, each species was assigned to one of five main trophic levels, namely: (1) primary producers (i.e. autotrophs, level 1); (2) primary consumers (i.e. herbivores, level 2); (3) secondary consumers (levels 2.1 to 2.9), (4) tertiary consumers (levels 3 to 3.9); and (5) quaternary consumers (levels 4 to 4.9).

Prior to assignment, the taxonomic nomenclature for individual species was verified using the World Register of Marine Species [38]. Next, individual species were assigned to one of the following five trophic categories as mentioned above. First, primary producers (or autotrophs) are considered to be trophic level 1. While autotrophs produce their own food, primary producers still have the potential to interact with MPs through attachment to outer appendages and may act as an entry point into the food web [39]. Second, primary consumers (or herbivores) include a variety of zooplankton, bivalves and reef fishes. Omnivores and carnivores occur throughout levels 2.1 to 4.5 and include a wide variety of organisms (e.g. bivalves, fishes, mammals) with a multitude of feeding strategies. Tertiary and quaternary consumers are often top predators and are an important component to marine food webs. These species are of particular interest due to the potential biomagnification of contaminants resulting from the consumption of lower tropic levels, as well as their eventual use for human consumption [40].

Following trophic assignment, the feeding habit of each individual species was also noted using the information provided by FishBase [36] and SeaLifeBase [37]. Organisms can exhibit a wide variety of feeding strategies which may affect MP uptake; namely filter feeding, grazing or browsing, selectively feeding on plankton, predator, scavenger and variable. Filter feeding organisms utilise the movement of external or internal appendages to produce a current, drawing particles in [41]. Grazers and browsers are herbivorous organisms that feed on algae growing along the substratum, usually by means of scraping [42]. Selectively feeding planktivores and predators use capture-based feeding where prey is obtained in a striking manner (e.g. meroplankton, reef fishes) [43]. Scavengers are organisms that sift through the benthos opportunistically consuming plant and/or animal matter [44]. Finally, variable indicates that species showcase multiple feeding strategies.

### Assessment of bioaccumulation and biomagnification

To assess whether bioaccumulation and biomagnification was evident in a general marine food web, standardised data on MP and chemical additive contamination derived from field observations or laboratory experiments were compared within and across trophic levels. For bioaccumulation, the presence and abundance of MPs and chemical additives for individual trophic levels, and for individual species within each trophic level was examined. To assess bioaccumulation, specific attention was given to field-based reports that provided both estimates of contaminant exposure and quantified contamination within a species, and to laboratory-based reports that provided estimates of contaminant exposure, as well as uptake and retention of contaminants within a species. For biomagnification, data was examined to determine whether contamination of MPs and chemical additives increased with increasing trophic levels. To assess biomagnification, specific attention was given to field-based studies that quantified levels of contamination across individual trophic levels, and to laboratory-based reports that contained a trophic transfer component.

## Results

### Selection of suitable contamination data

The systematic review of the literature identified a total of 1,357 publications (Fig 1, S1 Table in S1 File). Following screening of the 1,357 records on study organisms and contaminants of concern (i.e. MPs and/or associated additives), 295 records remained for further assessment of eligibility. Based on eligibility criteria, primarily around inability to standardise contamination data given the information presented in the study (S2a Table in S1 File), a total of 116 publications were included in the qualitative and quantitative assessment (S2b Table in S1 File). These studies include reports on MP contamination in both *in situ* (n = 87) and laboratory-based marine organisms (n = 20), as well as reports on chemical additive uptake as a result of MP uptake in both *in situ* (n = 2 out of 87) and laboratory-based marine organisms (n = 7). Also included in this study are laboratory-based experiments demonstrating the trophic transfer of MPs (n = 2).

Across the 87 studies investigating MP contamination in field collected organisms, a total of 23,049 individuals comprising 411 species across 7 phyla were examined. From these, 537 individual organisms from 94 species exhibited no MP contamination, with the remaining 22,512 individual organisms from 329 species showing contamination with MPs (S3a Table in S1 File). Contamination data for most species could be divided by sample size to obtain estimates of MPs individual^-1^; data for 11 species (all bivalve molluscs) were transformed from MPs g^-1^ to MPs individual^-1^ to enable inclusion in this study (S3b Table in S1 File). From 2 out of the 87 studies that examined *in situ* organisms, chemical additive uptake linked with MP ingestion was quantified for a total of 8 chemicals in 115 individual organisms from 3 species (S4 Table in S1 File).

Laboratory studies investigating MP uptake (n = 20) included a minimum of 1,610 individuals comprising 21 marine species across 6 phyla (S5 Table in S1 File). Transfer of MPs across two trophic levels was specifically investigated in three laboratory studies (S6 Table in S1 File). In addition, seven laboratory studies investigated the uptake of chemical additives as a result of MP uptake on marine biota (S7 Table in S1 File). Across these 7 studies, 581 individual organisms comprising 6 species across 2 phyla were analysed for contamination of 5 chemical additives.

### Contamination in primary producers

**Fig 2:**
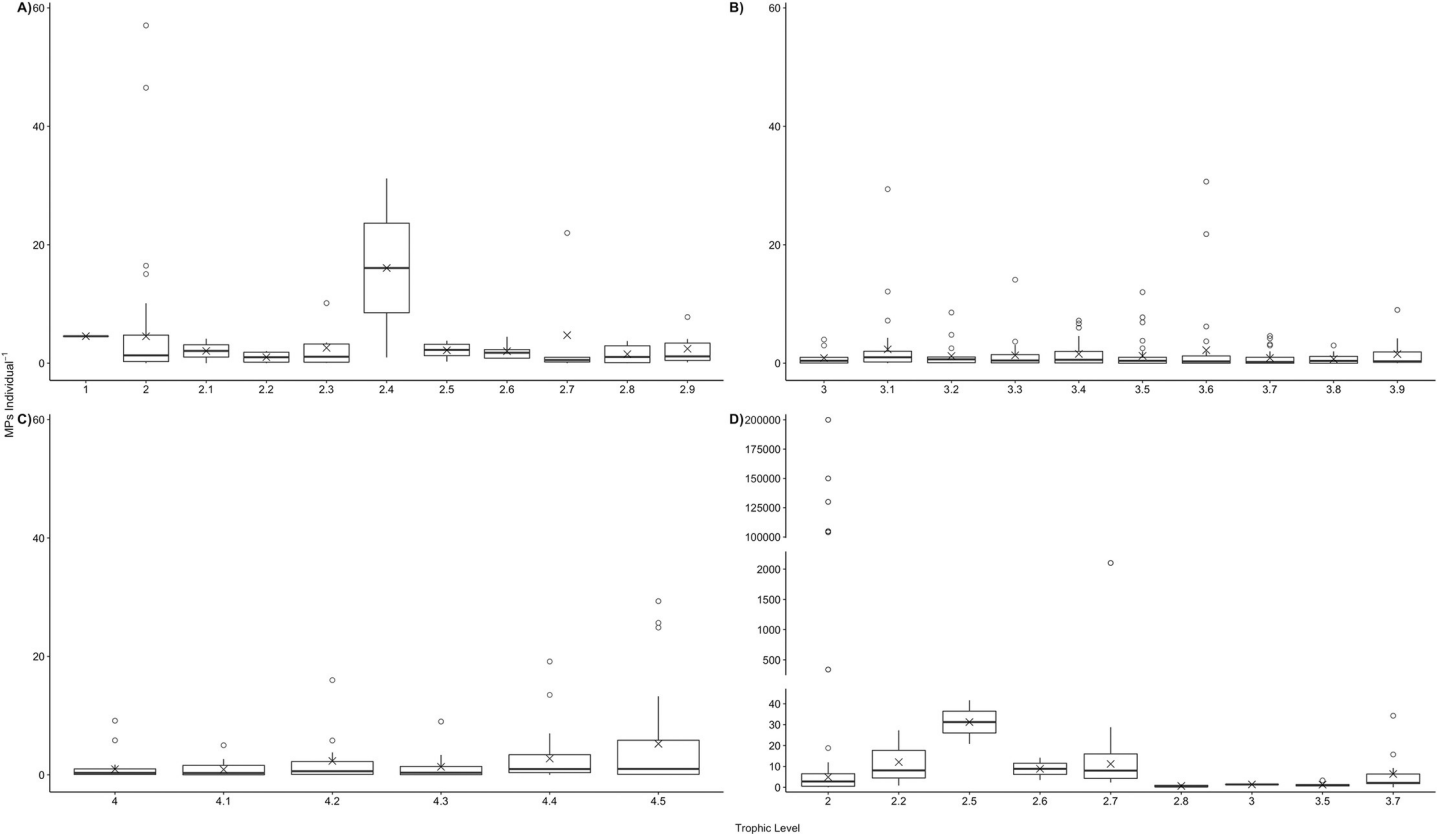
Body burden of bioaccumulated microplastics individual^-1^ estimated for different trophic levels, based on reports for marine species collected *in situ* (a) 1 to 2.9, (b) 3 to 3.9, and (c) 4 to 4.5, and exposed in laboratory experiments (d) 2 to 3.7. Data have been organised to show the minimum, first quartile, median, mean (X), third quartile, maximum and outliers (°). Trophic levels have been grouped into to a single decimal place, e.g. level 4.2 includes 4.21 to 4.29. Note different scales on y-axes in panels a-c) compared to panel d).

**Fig 3:**
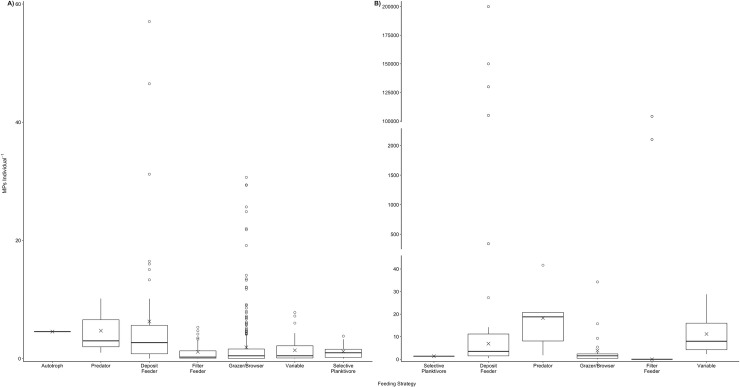
Body burden of bioaccumulated microplastics individual^-1^ estimated for different feeding strategies, based on reports for marine species collected *in situ* (a), and exposed in laboratory experiments (b). Data have been organised into feeding strategies and presented to show the minimum, first quartile, median, mean (X), third quartile, maximum and outliers (°). Note different scales on y-axes. Mean (X) values for (b) laboratory conditions are exclusive of outlier values.

**Fig 4:**
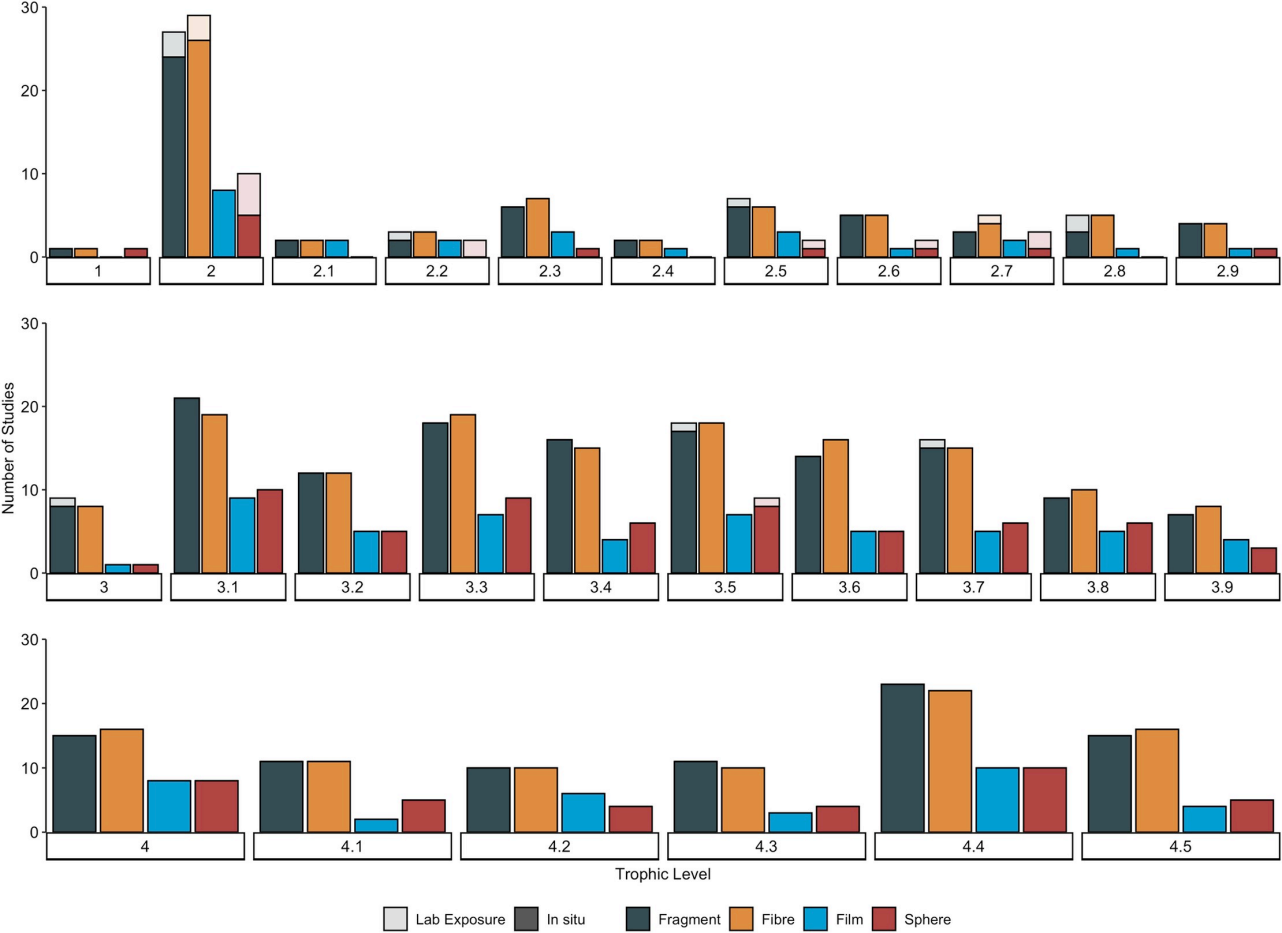
Frequency of microplastic (MP) shapes reported in studies on marine species collected *in situ* and exposed in laboratory experiments. Data has been organised by trophic level, which are grouped into to a single decimal place, i.e. level 4.2 includes 4.21 to 4.29. Microplastic shapes include those found in organisms collected from field samples (n = 87 studies; dark shaded on bottom) or used in laboratory experiments investigating MP uptake, including those focused on trophic transfer (n = 22 studies; light shaded on top). Details regarding the number and percentage of studies for each level have been provided in S8 Table in S1 File.

MP contamination of primary producers (trophic level 1) in the marine environment was only investigated in one study (S3a Table in S1 File) [45]. MPs were found within the epiphytic layer of the autotrophic seagrass *Thalassia testudinum* at a quantity of 4.56 MPs individual^-1^ (n = 16) (Figs 2A and 3). Contamination levels of the surrounding sampling area were not reported. While the shapes of putative MPs, including shapes including fibres, fragments and spheres, were indicative of MPs (Fig 4), their polymer types were not confirmed. Studies on primary producers that examined contamination with chemical additives *in situ*, or contamination with MPs or chemical additives under controlled laboratory exposures were not found.

### Contamination in primary consumers

A total of 41 publications on marine primary consumers (trophic level 2) contaminated with MPs and/or associated chemical additives were identified. Contamination of herbivores with MPs has been reported in both the field (n = 26 studies; S3a Table in S1 File) and in laboratory experiments (n = 12; S5 Table in S1 File). In contrast, only a few studies report on chemical additive contamination resulting from MP uptake by herbivores from *in situ* observations (n = 1; S4 Table in S1 File) or laboratory experiments (n = 4; S7 Table in S1 File).

#### a. Field observations

*In situ* herbivores have been found to exhibit contamination in 43 species across 4 phyla, with a total of 37 species. On average, herbivores were found to be contaminated with 4.55 ± 8.59 S.D. MPs individual^-1^ (n = 4,993) (Fig 2A). MP uptake was greatest in molluscs (6.97 ± 11.22 S.D. MPs individual^-1^; n = 3,135), followed by annelids (1.65 ± 1.48 S.D. MPs individual^-1^; n = 18), fishes (0.83 ± 1.68 S.D. MPs individual^-1^; n = 754), and arthropods (0.44 ± 0.48 S.D. MPs individual^-1^; n = 1,086). Herbivorous filter feeders demonstrated the highest level of contamination (6.83 ± 11.04 S.D. MPs individual^-1^; n = 21 sp.), followed by browsers/grazers (0.96 ± 1.71 S.D. MPs individual^-1^; n = 17 sp.), scavengers (0.96 MPs individual^-1^; n = 1 sp.), and selective planktivores (0.12 ± 0.15 S.D. MPs individual^-1^; n = 4 sp.) (Fig 3A). Five studies reported environmental MP contamination levels alongside MP uptake in primary consumers [46–50], however, different reporting units for environmental and organismal contamination makes direct comparisons difficult (Table 1). Notwithstanding, the MP body burdens reported for primary consumer species in these studies do not appear to support an accumulation of MPs within organisms compared to MP concentrations in the surrounding environments.

**Table 1:** Environmental contamination and bioaccumulation of microplastics (MP) for marine organisms (MPs individual^-1^) collected *in situ*.

Environment	Environmental Contamination	Unit (MPs per)	Trophic Level	Associated Species	MP	Location	Reference
					Individual^-1^		
Surface Water	659.9 ± 520.9	m^-3^	4.4	*Oncorhynchus tshawytscha*	1.15	Canada	[51]
Sediment	60.2 ± 63.4	kg^-1^ d.w.					
Surface Water	16,339–520,213	km^-2^	2	*Siganus luridus*	3.13	Turkey	[50]
			2.8	*Liza aurata*	3.26		
			3.1	*Mullus barbatus*	1.39		
				*Sardina pilchardus*	2.14		
			3.4	*Lithognathus mormyrus*	0.63		
				*Scomber japonicus*	6.71		
				*Serranus cabrilla*	1.50		
			3.5	*Mullus surmuletus*	1.18		
				*Pagellus erythrinus*	0.63		
				*Upeneus pori*	0.69		
			3.6	*Diplodus annularis*	1.96		
				*Pelates quadrilineatus*	1.48		
				*Upeneus moluccensis*	0.78		
			3.7	*Sparus aurata*	0.87		
			3.8	*Nemipterus randalli*	1.31		
				*Pagellus acarne*	1.63		
				*Pomadasys incisus*	0.79		
				*Sciaena umbra*	3.00		
				*Trachurus mediterraneus*	1.77		
			3.9	*Pagrus pagrus*	1.44		
			4	*Chelidonichthys lucerna*	0.75		
			4.1	*Caranx crysos*	5.00		
				*Dentex gibbosus*	0.29		
			4.3	*Argyrosomus regius*	1.84		
			4.5	*Saurida undosquamis*	1.22		
Surface Water	27	L^-1^	2	*Mytilus edulis*	1.23	North Sea	[46]
Sediment	48	kg^-1^ d.w.					
Surface Water	110	m^–3^	2	*Copepoda spp*.	0.33	Indian Ocean	[47]
Sediment	4.83 ± 2.44	ml^-1^	2	*Cerastoderma edule*	4.30	Atlantic Ocean	[48]
				*Hediste diversicolor*	2.70		
				*Pelecyora isocardia*	1.50		
				*Scolelepis squamata*	0.60		
				*Scrobicularia plana*	3.30		
				*Senilia senilis*	1.00		
			3	*Diopatra neapolitana*	1.00		
				*Glycera alba*	3.00		
Sub-surface Water (6 m)	2.4 ± 0.8	m^-3^	3.1	*Boreogadus saida*	0.22	Artic	[52]
			3.3	*Triglops nybelini*	0.39		
Sub-surface Water	1.39	m^-3^	3.3	*Callionymus lyra*	0.02	UK	[53]
				*Microchirus variegatus*	0.19		
			3.6	*Anguilla anguilla*	1.00		
			3.7	*Trisopterus minutus*	0.02		
Surface Sediment	560–4,205	kg^-1^ d.w.	2	*Acila mirabilis*	5.50	China	[49]
			3.19	*Crangon affinis*	29.40		

Environmental contamination data on MPs pertain to results reported for locations where marine species were collected; please note different units. Microplastic concentrations reported for marine species were standardised into number of MPs per individual organism (MPs individual^-1^; i.e. body burden) (see ‘Standardisation of contamination data’).

**Fig 5:**
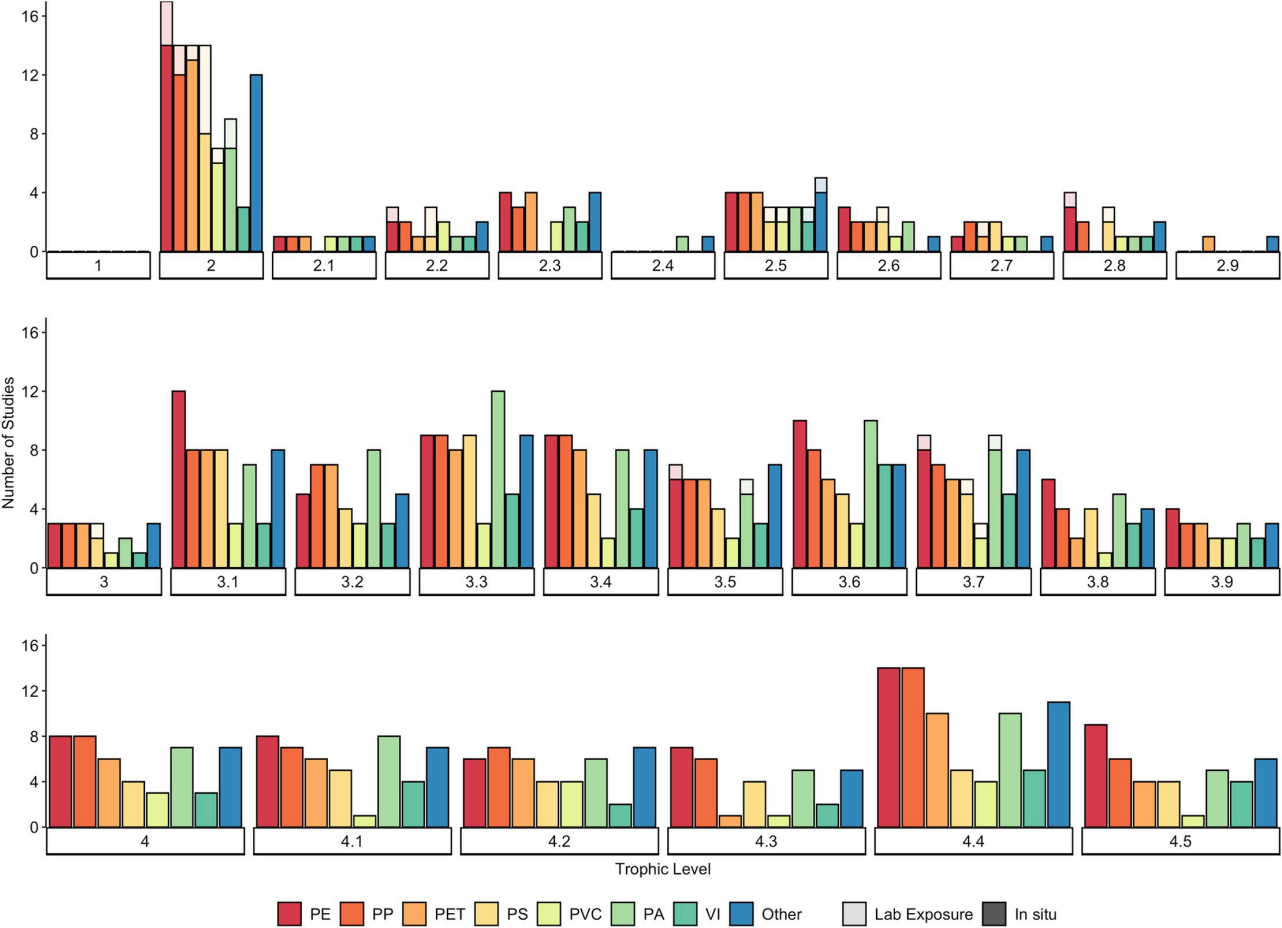
Frequency of microplastic (MP) polymer types confirmed in studies on marine species collected *in situ* and exposed in laboratory experiments. Data has been organised by trophic level, which are grouped into to a single decimal place, i.e. level 4.2 includes 4.21 to 4.29. Microplastic polymers include those found in organisms collected from field samples (n = 87 studies; dark shaded on bottom) or used in laboratory experiments investigating MP uptake, including those focused on trophic transfer (n = 22 studies; light shaded on top). Note that not all studies confirmed or reported MP polymer types. Details regarding the number and percentage of studies for each trophic level have been provided in S8 Table in S1 File. ‘Other’ includes less frequently found polymers such as: PAN, PMMA, CP, PC, ABS, EVA, PVA, PUR, PTFE, ASA, acrylic, alkyd and epoxy. Some varieties of polymers have been grouped together (i.e. PE includes HDPE, MDPE and LDPE; PET includes PET and PES).

The sizes of MPs detected ranges from 10 μm to 4.7 mm, while shapes included fibres, fragments, films and spheres (Fig 4). While not all studies confirmed polymer type of putative MPs detected in primary consumers, those that did found a wide range including polyethylene (PE), low-density polyethylene (LDPE), polypropylene (PP), polystyrene (PS), polyvinyl chloride (PVC), polyester (PES), viscose (VI; rayon), polyamide (PA; nylon), and others (Fig 5).

Only two herbivorous species, namely the bivalves *Mytilus edulis* and *Cerastoderma edule*, have been examined for contamination with chemical additives associated with MPs in their natural habitat (S4 Table in S1 File). Relatively high concentrations of phthalates were reported for *M*. *edulis* and C. *edule*, 26.36 and 52.36 ng g^-1^, with concentrations of polycyclic aromatic hydrocarbons (PAHs), polybrominated diphenyl ethers (PBDEs), and polychlorinated biphenyls (PCBs) substantially lower for both organisms [54]. Contamination levels in the surrounding environment were not measured.

#### b. Laboratory exposures

Evidence of MP uptake by herbivores under controlled laboratory exposures has been examined in 11 species and confirmed for 8 species (S5 Table in S1 File). While exposure conditions, such as durations, nominal MP concentrations, polymer types, sizes, and shapes varied, MP uptake ranged from 0 to 200,000 MPs individual^-1^ with an average of 25,596.57 ± 13,511.93 S.D. MPs individual^-1^ (n = 377) (Fig 2D). This extraordinary number is mainly a result of *C*. *helgolandicus*’ ingestion rates of 3,278 and 104,100 MPs individual^-1^ [16, 55] and *M*. *edulis*’ ingestion of 105,000 to 200,000 MPs individual^-1^ [56]. With those extreme values removed, average MP uptake drops to 25.62 ± 14.64 S.D. MPs individual^-1^ (n = 241). Measured MP exposure concentrations were not reported in any of these studies. Examination of MP egestion over a depuration period showed that MPs were retained in *A*. *compressa* for at least 36 hours [57], and in *T*. *gratilla* for fewer than 2 days [58].

A total of 4 species have been examined for contamination with chemical additive associated with MPs uptake under controlled laboratory exposures, namely the mussels *Mytilus edulis* [59] and *Mytilus spp*. [60], the clam *Scrobicularia plana* [61] and the amphipod *Allorchestes compressa* [57] (S7 Table in S1 File). Nominal exposure concentrations for PAHs (including fluoranthene and benzo[a]pyrene) and a range of PBDEs ranged from 5 ng to 100 μg l^-1^, with chemicals dosed alone, alongside MPs, or absorbed to MP particles. Uptake of chemical additives seemed to be highest when dosed alone (2,000 to 117,000 ng g^-1^) and lowest when absorbed to MPs (0 to 2,710 ng g^-1^), regardless of the chemical additive or organism used. None of the studies reported on measured exposure concentrations or on retention rates for these chemicals or quantified the MP uptake by the organisms examined.

### Contamination in secondary consumers

Overall, a total of 33 publications on marine secondary consumers (trophic level 2.1–2.9) contaminated with MPs and/or associated chemical additives were identified. Contamination of secondary consumers with MPs has been reported in both the field (n = 26 studies; S3a Table in S1 File) and in laboratory experiments (n = 7; S5 Table in S1 File). In contrast, only 3 studies report on chemical additive contamination resulting from MP uptake by secondary consumers from both *in situ* observations (n = 1; S4 Table in S1 File) or laboratory experiments (n = 2; S7 Table in S1 File).

#### a. Field observations

MP uptake by secondary consumers *in situ* has been investigated in 34 species, with 31 species exhibiting contamination, including various species of bivalves, echinoderms, arthropods, fishes and sea turtles. Overall, MP uptake by secondary consumers averaged 2.99 ± 6.40 S.D. MPs individual^-1^ (n = 2,755; Fig 2A). The highest levels of MP uptake are reported for molluscs (7.81 ± 20.67 S.D MPs individual^-1^; n = 434), arthropods (7.80 ± 10.05 S.D MPs individual^-1^; n = 900), and echinoderms (6.58 ± 5.06 S.D. MPs individual^-1^; n = 202) (S3a Table in S1 File). Lower levels were reported for sea turtles (*Chelonia mydas*; 2.3 ± 1.7 S.D. MPs individual^-1^; n = 53), ascidians (1.78 ± 1.12 S.D. MPs individual^-1^; n = 15), and fishes (1.39 ± 1.28 S.D. MPs individual^-1^; n = 1,151). When organised by feeding strategies, species with scavenging behaviours demonstrated the highest levels of MP contamination (6.58 ± 5.06 S.D. MPs individual^-1^; n = 2), followed by predators (5.44 ± 9.40 S.D. MPs individual^-1^) and filter feeders (5.27 ± 13.41 S.D. MPs individual^-1^); much lower levels were reported for species with variable feeding strategies, selective planktivores, and browsers/grazers (Fig 3A). Only one study reported environmental MP contamination levels alongside organism contamination (Table 1), with MP contamination in surrounding waters appearing to be much higher than levels found in the mullet *Liza aurata* [50]. The sizes of MPs detected ranged from 8 μm to 5 mm, while shapes included fibres, fragments, films, and spheres (Fig 4). Polymer types of MPs detected in secondary consumers were confirmed to include PE, PES, PA, PP, PET, PVC, VI, PS, and others (Fig 5).

Only one species, namely the ascidian *Microcosmus exasperates*, has been examined for contamination with chemical additives associated with MPs in their natural habitat. The highest concentrations were reported for bis(2-ethylhexyl) phthalate (DEHP) (range: 4,851–4,988 ng g^-1^; n = 15), followed by dibutyl phthalate (DBP) (range: 1,643–2,224 ng g^-1^; n = 15) [62] (S4 Table in S1 File). Contamination levels in the surrounding environment were not measured.

#### b. Laboratory exposures

Evidence of MP uptake by secondary consumers under controlled laboratory exposures has been documented in a total of 6 species, including bivalves, crustaceans, and fish; all species investigated exhibiting MP uptake (S5 Table in S1 File). While exposure conditions varied, average uptake by secondary consumers was 127.99 ± 853.44 S.D. MPs individual^-1^ (n = 566) (Fig 2D). When removing extreme uptake values for *Acanthochromis polyacanthus* (up to 2,102 MPs individual^-1^; [63]), average MP uptake is reduced to 11.87 ± 12.48 S.D. MPs individual^-1^ (n = 454). Measured MP exposure concentrations were not reported in any of these studies. Examination of MP egestion over a 48 hr depuration period showed that MPs were retained in *Palaemonetes pugio* for an average of 43 hrs, ranging from approximately 28 to 76 hrs depending on the MP polymer type [64]. Egestion of MPs by the mussel *Mytilus galloprovincialis* was 80% within the first 24 hrs, and 100% within 8 days [65].

Only one species, namely the bivalve *Mytilus galloprovincialis*, has been examined for contamination with chemical additive associated with MPs uptake under controlled laboratory exposures [66, 67] (S7 Table in S1 File). Nominal exposure concentrations for benzo[a]pyrene and pyrene ranged from 0.15 μg l^-1^ to 15,000 ng g^-1^ and 200 to 260 ng g^-1^, respectively, with additives either dosed alone or absorbed to MP particles. Uptake was greatest (470 ng g^-1^) in *M*. *galloprovincialis* following a 7 day exposure to pyrene absorbed to PE and PS [66]. Following a 28-day exposure, uptake of benzo[a]pyrene in the mussel’s digestive glands was higher when dosing alone (35 ng g^-1^) compared to when absorbed with low-density polyethylene (LDPE; 30 ng g^-1^) [67]. Beno[a]pyrene was found to accumulate over time in both the digestive gland and the gills, irrespective of exposure pathway [67]. Neither study reported on measured exposure concentrations or on retention rates for these chemicals or quantified the MP uptake by the bivalve examined.

### Contamination in tertiary consumers

Marine tertiary consumers (trophic level 3–3.9) were investigated and found contaminated with MPs and/or associated chemical additives in 50 publications. Contamination of these consumers with MPs has been reported in both the field (n = 44 studies; S3a Table in S1 File) and in laboratory experiments (n = 4; S5 Table in S1 File). In contrast, only two studies report on chemical additive contamination resulting from MP uptake by tertiary consumers from laboratory experiments (S7 Table in S1 File).

#### a. Field observations

*In situ*, evidence of MP uptake by tertiary consumers has been investigated in 224 species across 5 phyla and confirmed in 175 of these species (S3a Table in S1 File). On average, MP uptake by tertiary consumers was 1.47 ± 3.46 S.D. MPs individual^-1^ (n = 10,758) (Fig 2B). MP uptake was greatest in arthropods (8.15 ± 16.37 S.D. MPs individual^-1^; n = 300), largely due to high MP uptake by the shrimp *Crangon affinis* (29.40 MPs individual^-1^) [49]. In contrast, MP uptake was lower in annelids (2.00 ± 1.41 S.D MPs individual^-1^; n = 5), fishes (1.39 ± 2.97 S.D. MPs individual^-1^; n = 10,256), and sea turtles (1.5 ± 0.80 S.D. MPs individual^-1^; n = 49). Tertiary consumers that exhibit predator (n = 7,897) and selective planktivorous (n = 2,753) behaviour had the highest amount of contamination (1.53 ± 3.82 and 1.36 ± 1.64 S.D. MPs individual^-1^, respectively), with slightly lower contamination levels in organisms with scavenger (n = 4), variable (n = 94) and filter feeding (n = 10) strategies; Fig 3A). Only five studies reported environmental MP contamination levels alongside organism contamination (Table 1). Overall, MP contamination in surrounding environments appear to be much higher than in the polychaete worms *Glycera alba* and *Diopatra neapolitana* [48], the shrimp *Crangon affinis* [49], and in various fish species [50, 52]. Only one study found comparable levels of MP contamination in both surrounding waters (range: 0.26 to 3.79 MPs m^-3^) and in fish larvae (range: 0.02 to 4.8 MPs individual^-1^, n = 156) [53]. The sizes of MPs detected range from 10 μm to 5 mm, with the majority being smaller than 2 mm; shapes included fibres, fragments, films, and spheres (Fig 4). Polymer types of MPs detected in tertiary consumers were confirmed to include PET, PE, PVC, PP, PA, PS, PES, VI, and others (Fig 5).

#### b. Laboratory exposures

MP uptake by tertiary consumers under controlled laboratory exposures has been reported for 4 species, namely the polychaete worm *Arenicola marina* and the teleost fishes *Dicentrarchus labrax*, *Seriolella violacea*, and *Sparus aurata* (S5 Table in S1 File). While exposure conditions varied, average uptake by tertiary consumers was 4.76 ± 2.82 S.D. MPs individual^-1^ (n = 617) (Fig 2D). The highest average uptake was recorded for the seabream *S*. *aurata* (6.97 ± 10.13 S.D. MPs individual^-1^; n = 165), and the lowest for the palm ruff *S*. *violacea* (0.75 ± 0.15 S.D. MPs individual^-1^; n = 132). Measured MP exposure concentrations were not reported in any of these studies. Examination of MP egestion over a depuration period showed that MPs were retained for fewer than 2 days in *D*. *labrax* [68], for an average of 4.4 ± 0.9 days in *S*. *violacea* [69], and for more than 30 days in *S*. *aurata* [70].

Only one species, namely the lobster *Nephrops norvegicus*, has been examined for contamination with chemical additive associated with MPs uptake under controlled laboratory exposures. Exposure to a nominal exposure concentration of 1.34 μg for PCBs either dosed alone, dosed alongside MPs, or absorbed to MP revealed that uptake of PCBs was highest when exposed to the chemical additive alone [71]. The study did not report on measured exposure concentrations or on retention rates for PCBs or quantified the MP uptake by the bivalve examined.

### Contamination in quaternary consumers

A total of 42 publications on marine quaternary consumers (trophic level 4–4.9) contaminated with MPs and/or associated chemical additives were identified (S3a Table in S1 File). All these studies reported on MP uptake in the field, with no reports on chemical uptake associated with MP contamination *in situ*, or on MP or chemical additive contamination from controlled laboratory exposures.

#### a. Field observations

MP uptake by quaternary consumers *in situ* has been investigated in a total of 109 species, and confirmed for 85 species, including fish, sea turtles and marine mammals (S3a Table in S1 File). On average, quaternary consumers have ingested 2.42 ± 5.30 S.D. MPs individual^-1^ (n = 4,527; Fig 2C). MP uptake was greatest in cetaceans (11.06 ± 8.72 S.D. MPs individual^-1^; n = 225), followed by cartilaginous (1.25 ± 0.50 S.D. MPs individual^-1^; n = 9), and ray-finned fishes (1.04 ± 1.93 S.D. MPs individual^-1^; n = 4,131) (S3a Table in S1 File). The lowest level of MP uptake was reported for elasmobranchs (0.27 ± 0.10 MPs individual^-1^; n = 160). The baleen humpback whale, *Megaptera novaeangliae*, was the only filter-feeding quaternary consumer to be investigated, exhibiting the highest MP uptake of 16 MPs individual^-1^ (n = 1). In contrast, quaternary consumers that exhibit predator (n = 4,478) or variable (n = 48) feeding strategies contained substantially less MP contamination (2.34 ± 5.20 and 0.85 ± 0.84 S.D. MPs individual^-1^, respectively; Fig 3A). Two studies reported environmental MP contamination levels alongside organism contamination [50, 51] (Table 1). In both cases, the MP contamination in the waters surrounding fishes appears to be higher than levels detected in the organisms themselves. The sizes of MPs detected ranges from 10 μm to 5.0 mm with the majority being between 500 μm and 3 mm. Shapes included fragments, films, spheres, and fibres (Fig 4). Polymer types of MPs detected in quaternary consumers were confirmed to include PET, PA, PP, PE, PVC, PS, PES, VI, and others (Fig 5).

### Evidence for biomagnification across a general marine food web

Finally, to assess whether biomagnification was evident in a general marine food web, data was examined to determine whether contamination of MPs and chemical additives increased with increasing trophic level, using the standardised data for each trophic level from the 87 *in situ* reports (S3a Table in S1 File). The 2 laboratory-based reports that contain a trophic transfer component were also considered (S6 Table in S1 File).

#### a. Field observations

Across the 5 main tropic levels there was no apparent increase in MP bioaccumulation with increasing trophic level, based on the estimated average MPs individual^-1^ for each of these 5 levels derived from a total of 411 species (22,987 individuals) collected *in situ* (S3a Table in S1 File; Fig 2). On average, MP contamination is highest for herbivores (trophic level 2) at 4.55 ± 8.59 S.D. MP individual^-1^ (n = 4,993), and lowest for tertiary consumers (trophic level 3 to 3.9) at 1.47 ± 3.46 S.D. MP individual^-1^ (n = 10,738). Within the 5 tropic levels, the only slight increase in average MP body-burden was observed from trophic level 4 to 4.5 (Fig 2C). The slightly higher average MP individual^-1^ in trophic level 4.5 could be largely attributed to relatively high levels of contamination in marine mammals (S3a Table in S1 File). Notwithstanding, by far the highest average MP individual^-1^ was not in these highest tropic levels, but in secondary consumer trophic level 2.4 (Fig 2A) caused by the high bio-burden in the filter-feeding mussel *Perna perna* [72]. Indeed, rather than biomagnification of MPs across trophic levels, the body burden of MPs in marine species appears to be more influenced by feeding strategy (Fig 3A & 3B). Filter feeders have, on average higher levels of MP contamination than any of the other feeding strategies, both *in situ* and under laboratory conditions (6.62 ± 11.03 S.D. MP individual^-1^, n = 3,975; 32,523.89 ± 65,800.44 S.D. MP individual^-1^, n = 319, respectively). However, with outliers removed, laboratory-exposed grazers and browsers demonstrate higher levels of MP contamination on average (18.23 ± 15.24 S.D. MP individual^-1^; n = 142; Fig 3B)

*In situ* biomagnification of chemical additives as a result of MP uptake in a general marine food web cannot be supported nor refuted based on the current literature. Only three marine species across two trophic levels have been examined for contamination with chemical additives associated with MPs in their natural habitat [54, 62] (S4 Table in S1 File). The chemical additives examined differ across the two trophic levels, with phthalates (including benzyl butyl phthalate [BBP], diethyl phthalate [DEP], dimethyl phthalate [DMP], diethylhexyl adipate [DEHA], DEHP and DBP), PAHs, PBDEs, and PCBs quantified in two species categorised as primary consumer [54], and only DEHP and DBP quantified in one species categorised as secondary consumer [62].

#### b. Laboratory exposures

Two studies have demonstrated the trophic transfer of MPs between marine species, although neither of these studies quantified MP uptake (S6 Table in S1 File). Two experiments included feeding pre-exposed *Mytilus edulis* mussels (n = 24 to 50) to crabs *Carcinus maenas* (n = 24 to 42) [4, 73]. Microplastic retention times for *C*. *maenas* ranged from 14 to 21 days but were not estimated for *M*. *edulis* as these studies assumed the immediate consumption of mussels by crabs. None of the laboratory studies reviewed examined potential trophic transfer of chemical additives associated with MP uptake in marine species.

## Discussion

The aim of this review was to examine whether current, published findings support the premise that MPs, and their chemical additives, bioaccumulate and biomagnify across a general marine food web, a notion often inferred in the literature on marine MP contamination [4–6]. Following a systematic review of the global literature, data was synthesised from 116 publications that quantified MP contamination for a total of 23,049 individuals from 411 marine species in their natural habitat (n = 87 articles), and at least 1,610 individuals from 21 marine species in laboratory settings (n = 20 articles). Our results corroborate previous studies [74, 75] that bioaccumulation of MPs occurs in numerous individual marine species across four main trophic levels representing consumers, with MP contamination of primary producers also reported [45]. Further, bioaccumulation of chemical additives associated with MP uptake has also been documented, albeit in fewer species [54, 57, 71]. Interestingly, in all six species examined, uptake of chemical additives was higher when exposed to the chemical alone compared to exposure alongside or on MPs [57, 59, 60, 67, 71]. For most of the studies reporting bioaccumulation of MPs or chemical additives, the concentrations in the surrounding environment were not measured, hindering the elucidation of potential exposure pathways. In contrast to bioaccumulation, biomagnification of MPs across the five main trophic levels is not supported by field-based MP uptake data, although trophic transfer has been reported in two laboratory studies [4, 73]. *In situ* biomagnification of chemical additives as a result of MP uptake cannot be supported nor refuted, due to only a few studies examining different chemical additives [54, 62]. Finally, the body burden of MPs in marine species appears to be more influenced by feeding strategy rather than biomagnification [28, 29], a finding that may well be true for chemical additives as well.

### Evidence for bioaccumulation

For this review, bioaccumulation was defined as the net uptake of MPs (or chemical additives) from the environment by all possible routes (e.g. contact, ingestion, respiration) from any source (e.g. water, sediment, prey) [24, 25]. Results confirm bioaccumulation of MPs in numerous individual marine species constituting a general marine food web, in both field collected and laboratory exposed organisms. On average, however, the body burden for most marine species collected *in situ* could be considered low, with many reports of zero MP uptake for individual species and individuals within species [76–78]. Indeed, an apparent low incidence of marine debris (including MPs) uptake has been reported previously, with more than 80% of >20,000 individual coastal, marine and oceanic fish examined not containing any marine debris [79]. The relatively low body burden is likely to reflect the inclusion of all organisms in our quantification of MP individual^-1^ for each species, a more representative estimate of MP bioaccumulation than only including the number of organisms that exhibit contamination [77, 80]. More broadly, a potential publication bias towards effects (i.e. detecting MP contamination in marine species) versus no effects (i.e. not detecting MP contamination) may have influenced our findings, although the existence and scale of such a bias in the MP literature is currently unknown [81, 82]. Further, the large variety of methodological procedures used to quantify and report on MP contamination in marine organisms [2, 83, 84] is likely to have affected our estimates of MP bioaccumulation. For example, polymer type is not always confirmed using spectroscopy or polarised light microscopy [85, 86], a crucial step in the analysis workflow for MP quantification [87], potentially resulting in over-estimating MP contamination. Conversely, the *a priori* exclusion of microfibres in marine samples as potential contamination [28, 88] may result in under-estimates of MP bioaccumulation. Combined, while findings are based on the most exhaustive review of the global literature on MP contamination in marine organisms to date, future MP bioaccumulation estimates will likely be more robust with the development of agreed standardized procedures for sample processing and MP characterisation [1].

Bioaccumulation of chemical additives associated with MP uptake has been reported upon much less frequently than physical MP bioaccumulation, both *in situ* and in controlled laboratory experiments. Across all three marine species collected from the field, namely the clam *Cerastoderma edule*, the mussel *Mytilus edulis*, and the ascidian *Microcosmus exasperatus*, the concentrations of individual or combined phthalates were highest among the different chemical additives examined [54, 62]. This is not surprising as phthalates are primarily used as plasticisers and commonly detected in the oceanic environment [3]. Indeed, other studies have speculated chemical contamination of marine organisms that was indicative of plastic contamination in the marine environment [89, 90]. Interestingly, phthalate body burden did not increase with MP bioaccumulation across these three marine species suggesting that the two may not be positively correlated. A comparative study examining phthalate and MP body burden within a single species across different levels of environmental contamination would further elucidate uptake of chemical additives associated with MPs *in situ*. Indeed, bioaccumulation of chemical additives was consistently, and often several magnitudes higher, following laboratory exposures of additives only compared to additives on MPs [57, 59, 60, 67, 71]. Combined, these results would strongly suggest that environmental exposure to chemical additives *per se* affects bioaccumulation in marine organisms more strongly than exposure to chemical additives associated with MPs [91–93].

Comparing MP bioaccumulation to *in situ* MP exposure concentrations revealed that for most, if not all, marine species the reported MP body burdens do not appear to support an accumulation of MPs within species relative to the surrounding environment. However, different reporting units for organismal and environmental contamination levels makes direct comparisons difficult, an issue identified for marine debris research previously [94]. Previous studies detected higher number of MP particles in coastal fish collected from locations with higher MP particles in surrounding seawater and sediment [50]. While chemical additives have been detected in field-collected marine species, neither of these studies measured their concentrations in the surrounding environment [54, 62]. Repeated field sampling of marine species, in particular from early to mature life history stages, combined with measurements of exposure concentrations will assist in elucidating whether MPs and/or chemical additives accumulate over time. Such studies would also provide critical information for more realistic and comparative laboratory studies, including environmentally relevant exposure characteristics such as concentrations, polymer type, and plastic size, shape and colour, a recommendation raised in previous reviews [95–97]. Many of these characteristics have been demonstrated to affect retention, and thus bioaccumulation of MPs [64, 98, 99], but have rarely been examined using environmentally relevant exposures. Currently, comparisons between exposure and uptake of MPs and chemical additives in controlled laboratory studies are further complicated by the absence of measured versus nominal concentrations [16, 100]. Improved quantification of different exposure pathways, such as respiration, direct uptake or indirect uptake via prey, would elucidate their relative importance in bioaccumulation. Such research should also consider that MP uptake by marine organisms can be non-random [101] and highly selective [102, 103], including active rejection of MPs [58, 104]. Taking these caveats into account will result in improved estimates of bioaccumulation, subsequent trophic transfer and potential biomagnification of MPs at higher trophic levels [75].

### Evidence for biomagnification

For this study, biomagnification was defined as the increase in concentration of MPs (or chemical additives) due to trophic transfer from lower to higher trophic levels [24, 25]. The findings on bioaccumulation for different trophic levels do not support *in situ* biomagnification of either MPs or associated additives within a general marine food web. More specifically, there is no evidence based on current, published findings for an increase in average bioaccumulation of MPs and associated additives from lower to higher trophic levels across a general marine food web. In fact, trophic level 2.4 (secondary consumers) exhibited by far the highest average MP bioaccumulation, and trophic level 2 (primary consumers) showed the highest values of MP body burden across the general marine food web. These findings, based on a broad overview, do not negate the notion that trends of MP biomagnification may differ when taking a targeted approach based on smaller geographic scales, on species-specific food chains, or on future projections of MP contamination. Additionally, the lack of evidence for *in situ* biomagnification of chemical additives as a result of MP uptake is primarily due to a lack of suitable data to support or refute such biomagnification. Such lack of data does not equate to evidence for or against biomagnification, a concept previously addressed for the MP literature [105], but rather that it remains uncertain based on current, published findings. This highlights the need for more careful inference of potential effects and ecological risks of marine MP contamination based on available evidence. Further, whether leaching of chemical additives from MPs into organisms occurs is currently unclear and requires further investigation for assessments of potential bioaccumulation and biomagnification. In laboratory experiments, trophic transfer has been reported from the mussel *Mytilus edulis* to the crab *Carcinus maenas* [4, 73]; however, it is unclear whether this resulted in biomagnification as MP presence in either prey or predator was not quantified. Importantly, the immediate consumption of contaminated mussels disregards bioaccumulation kinetics of MPs in prey and predator-prey interactions that would occur in the field [75, 91]. If trophic transfer of MPs is occurring *in situ*, results of this study imply that MPs ingested via prey items are not being completely retained within the next tropic level. Rather, MPs may become entangled in biological material during digestion by the predator and simply pass through as egested material. One line of evidence for trophic transfer in the field would be to document contaminated prey items from within the digestive tract of a consumer species, a feat not achieved so far for MPs. We are aware of only one study which found plastic particles (size not reported) in post-hatchling sea turtle stomachs recovered from fish [106]. Finally, bioaccumulation of chemical additives associated with MP ingestion in the field has only been reported from a single trophic level [54, 62], while trophic transfer has not been examined in controlled laboratory exposures, precluding analysis of potential biomagnification.

Rather than biomagnification through trophic transfer, results of this study corroborate previous studies that MP bioaccumulation is strongly linked with feeding strategies of marine species [28, 29]. Field studies support this finding, with MP body burden being higher in pelagic fish species compared to demersal species irrespective of trophic level [50]. MP bioaccumulation in fish larvae from the English Channel [53] were also higher compared to adult fish from the Arctic [52], despite similar levels of MP contamination in surrounding waters. This likely reflects their feeding strategies with fish larvae filter-feeding continuously and unselectively on suspended particulate matter [53, 107], and adult *Triglops nybelini* and *Boreogadus saida* being selective predators that feed with a striking manner [36]. Similarly, omnivorous juveniles of the fish *Girella laevifrons* were shown to have a higher MP body burden (specific quantity not reported) compared to other intertidal fish species categorised as grazing herbivores or selective carnivores [29]. Higher MP contamination has been previously reported in selective predators compared to deposit and filter feeders, although Bour et al. [28] suggest caution in these results due to limited sample sizes and the exclusion of fibres. Further, exposure to 50 MP ml^-1^ resulted in higher MP body burdens in the filter feeding mussel *Mytilus edulis* [56] compared to the selective-feeding omnivorous shrimp *Palaemonetes pugi* [64], despite a shorter exposure time. Comparative studies examining MP body burden in organisms with varying feeding strategies following uniform exposures will aid quantifying the role of feeding strategies in influencing bioaccumulation of MPs.

The rationale behind assessing whether MP concentrations increased from lower to higher trophic levels stems from the classical concepts of bioaccumulation and biomagnification which is primarily applied to dissolved chemicals [20]. For physical items such as MPs, these end points may not completely suitable as chemicals and physical items would not interact with a marine organism in similar ways. Rather, physical MPs generally only come into contact with body cavities designed to pass material (i.e. gills or gastrointestinal tract). Translocation into other organs may occur via phagocytosis, albeit this is size dependent favouring smaller size classifications [108]. Conversely, chemicals are readily dissolved and the potential pathways for uptake by the marine organism are greater, including into organs other than gills and gastrointestinal tracts. Therefore, whether the concepts of bioaccumulation and biomagnification are suitable for assessing the ecological risks of MP contamination in marine environments needs further and more detailed consideration.

## Conclusions

Bioaccumulation and biomagnification of MPs, and associated chemical additives, in marine environments are often inferred in the literature on marine MP contamination. This review demonstrates that MP contamination occurs across all five main trophic levels in a general marine food web. Moreover, bioaccumulation of MPs occurs in numerous individual marine species across four main trophic levels representing consumers. The relative importance of different exposure pathways contributing to MP bioaccumulation, however, is not necessarily clear and needs further examination. While chemical additives have been detected in a few marine species collected *in situ*, results from laboratory exposures indicate that environmental exposure to chemical additives *per se* affects bioaccumulation more strongly than exposure to chemical additives associated with MPs. In contrast to MP bioaccumulation, this meta-analysis of *in situ* studies does not support biomagnification of MPs from lower to higher trophic levels in a general marine food web, even though trophic transfer of MPs has been reported in a few laboratory studies. Indeed, MP bioaccumulation appears to be more strongly linked with feeding strategies, rather than trophic levels, of marine species. Finally, bioaccumulation and biomagnification are two critical concepts used in ecological risk assessments to determine the extent of pollutant transport within food webs. This review highlights the need for targeted field-based and experimental studies to elucidate the possible routes of uptake of MPs (and associated chemicals) and provide confidence in the use of these endpoints in the MP literature.

## Supporting information

S1 File (DOCX)

## Abbreviations

ABS: Acrylonitrile butadiene styrene
ASA: Acrylonitrile styrene acrylate
BBP: Benzyl butyl phthalate
CP: Cellophane
DBP: Dibutyl phthalate
DEP: Diethyl phthalate
DEHA: Diethylhexyl adipate
DEHP: Bis(2-ethylhexyl) phthalate
DMP: Dimethyl phthalate
DnOP: Di-n-octylphthalate
EVA: Ethylene-vinyl acetate
FTIR: Fourier transform infrared spectroscopy
HBCD: Hexabromocyclododecane
HDPE: High Density Polyethylene
LDPE: Low Density Polyethylene
MDPE: Medium Density Polyethylene
PA: Polyamide
PAA: Polyacrylic Acid
PAH: Polycyclic aromatic hydrocarbons
PAN: Polyacrylonitrile
PBDE: Polybrominated diphenyl ether
PC: Polycarbonate
PCB: Polychlorinated biphenyl
PE: Polyethylene
PES: Polyester
PET: Polyethylene Terephthalate
PLA: Polylactic Acid
PMMA: Poly(methyl methacrylate)
PP: Polypropylene
PS: Polystyrene
PTFE: Polytetrafluoroethylene
PUR: Polyurethane
PVA: Poly(vinyl alcohol)
PVC: Polyvinyl Chloride
VI: Viscose
