# Supplementary material for: Bioaccumulation and biomagnification of microplastics in marine organisms: A review and meta-analysis of current data
Source: PLoS One. 2020 Oct 16;15(10):e0240792. doi: 10.1371/journal.pone.0240792 (PMC7567360; doi:10.1371/journal.pone.0240792)
Supplement: S1 File — (DOCX) [file pone.0240792.s001.docx]

Bioacccumulation and biomagnification of microplastics in marine organisms: A review and meta-analysis of current data

*Supporting Information*

Michaela E. Miller^1,2,3*^, Mark Hamann^3^, and Frederieke J. Kroon^1,2^

^1^Australian Institute of Marine Science (AIMS), Townsville, Queensland 4810, Australia

^2^AIMS@JCU, Division of Research and Innovation, James Cook University, Townsville, Queensland 4811, Australia

^3^College of Science and Engineering, James Cook University, Townsville, Queensland 4811, Australia

*Corresponding author

E-mail: [m.miller@aims.gov.au](mailto:m.miller@aims.gov.au) (MM)

Table of Contents

[Tables 3](#_Toc42285203)

[S1 Table. PRISMA Checklist for systematic review and meta-analysis. 3](#_Toc42285204)

[S2 Table. Full-text articles excluded from (a) and included in (b) the present study 6](#_Toc42285205)

[(a) Publications excluded 6](#_Toc42285206)

[(b) Publications included 9](#_Toc42285207)

[S3 Table. Bioaccumulation of (a) microplastics (MPs) for marine organisms collected in situ and (b) relevant data calculations. 11](#_Toc42285208)

[(a) MP contamination for marine organisms *in situ* 11](#_Toc42285209)

[(b) MP contamination calculations 26](#_Toc42285210)

[S4 Table. Bioaccumulation of chemical additives associated with microplastic (MP) uptake for marine organisms collected in situ. 28](#_Toc42285211)

[S5 Table. Experimental details of microplastic laboratory exposures conducted with marine organisms 29](#_Toc42285212)

[S6 Table. Trophic transfer of microplastics (MPs) for marine organisms. 32](#_Toc42285213)

[S7 Table. Experimental details of chemical additive laboratory exposure conducted with marine organisms 33](#_Toc42285214)

[S8 Table. Number of field and laboratory studies investigating microplastic (MP) uptake in marine organisms for each trophic level 35](#_Toc42285215)

[Figures 36](#_Toc42285216)

[S1 Fig. Flow diagram outlining the standardisation of microplastic (MP) contamination data for marine organisms. 36](#_Toc42285217)

[References 37](#_Toc42285218)

# Abbreviations

| **ABS** | Acrylonitrile butadiene styrene |
| --- | --- |
| **ASA** | Acrylonitrile styrene acrylate |
| **BBP** | Benzyl butyl phthalate |
| **CP** | Cellophane |
| **DBP** | Dibutyl phthalate |
| **DEP** | Diethyl phthalate |
| **DEHA** | Diethylhexyl adipate |
| **DEHP** | Bis(2-ethylhexyl) phthalate |
| **DMP** | Dimethyl phthalate |
| **DnOP** | Di-n-octylphthalate |
| **EVA** | Ethylene-vinyl acetate |
| **FTIR** | Fourier transform infrared spectroscopy |
| **HBCD** | Hexabromocyclododecane |
| **HDPE** | High Density Polyethylene |
| **LDPE** | Low Density Polyethylene |
| **LDPVC** | Low Density Polyvinyl Chloride |
| **MDPE** | Medium Density Polyethylene |
| **PA** | Polyamide |
| **PAA** | Polyacrylic Acid |
| **PAH** | Polycyclic aromatic hydrocarbons |
| **PAN** | Polyacrylonitrile |
| **PBDE** | Polybrominated diphenyl ether |
| **PC** | Polycarbonate |
| **PCB** | Polychlorinated biphenyl |
| **PE** | Polyethylene |
| **PES** | Polyester |
| **PET** | Polyethylene Terephthalate |
| **PLA** | Polylactic Acid |
| **PMMA** | Poly(methyl methacrylate) |
| **PP** | Polypropylene |
| **PS** | Polystyrene |
| **PTFE** | Polytetrafluoroethylene |
| **PUR** | Polyurethane |
| **PVA** | Poly(vinyl alcohol) |
| **PVC** | Polyvinyl Chloride |
| **VI** | Viscose |

# Tables

## S1 Table. PRISMA Checklist for systematic review and meta-analysis.

| **Section/topic** | **#** | **Checklist item** | **Reported on page #** |
| --- | --- | --- | --- |
| **TITLE** | | |  |
| Title | 1 | Identify the report as a systematic review, meta-analysis, or both. | 1 |
| **ABSTRACT** | | |  |
| Structured summary | 2 | Provide a structured summary including, as applicable: background; objectives; data sources; study eligibility criteria, participants, and interventions; study appraisal and synthesis methods; results; limitations; conclusions and implications of key findings; systematic review registration number. | 2 |
| **INTRODUCTION** | | |  |
| Rationale | 3 | Describe the rationale for the review in the context of what is already known. | 5 |
| Objectives | 4 | Provide an explicit statement of questions being addressed with reference to participants, interventions, comparisons, outcomes, and study design (PICOS). | 6 |
| **METHODS** | | |  |
| Protocol and registration | 5 | Indicate if a review protocol exists, if and where it can be accessed (e.g., Web address), and, if available, provide registration information including registration number. | N/A |
| Eligibility criteria | 6 | Specify study characteristics (e.g., PICOS, length of follow-up) and report characteristics (e.g., years considered, language, publication status) used as criteria for eligibility, giving rationale. | 7-8 |
| Information sources | 7 | Describe all information sources (e.g., databases with dates of coverage, contact with study authors to identify additional studies) in the search and date last searched. | 7-8 |
| Search | 8 | Present full electronic search strategy for at least one database, including any limits used, such that it could be repeated. | 7-8 |
| Study selection | 9 | State the process for selecting studies (i.e., screening, eligibility, included in systematic review, and, if applicable, included in the meta-analysis). | 7-8 |
| Data collection process | 10 | Describe method of data extraction from reports (e.g., piloted forms, independently, in duplicate) and any processes for obtaining and confirming data from investigators. | 7-8 |
| Data items | 11 | List and define all variables for which data were sought (e.g., PICOS, funding sources) and any assumptions and simplifications made. | 7-9 |
| Risk of bias in individual studies | 12 | Describe methods used for assessing risk of bias of individual studies (including specification of whether this was done at the study or outcome level), and how this information is to be used in any data synthesis. | 7 |
| Summary measures | 13 | State the principal summary measures (e.g., risk ratio, difference in means). | N/A |
| Synthesis of results | 14 | Describe the methods of handling data and combining results of studies, if done, including measures of consistency (e.g., I^2^) for each meta-analysis. | 9 |

| Risk of bias across studies | 15 | Specify any assessment of risk of bias that may affect the cumulative evidence (e.g., publication bias, selective reporting within studies). | 27 |
| --- | --- | --- | --- |
| Additional analyses | 16 | Describe methods of additional analyses (e.g., sensitivity or subgroup analyses, meta-regression), if done, indicating which were pre-specified. | N/A |
| **RESULTS** | | |  |
| Study selection | 17 | Give numbers of studies screened, assessed for eligibility, and included in the review, with reasons for exclusions at each stage, ideally with a flow diagram. | 12 |
| Study characteristics | 18 | For each study, present characteristics for which data were extracted (e.g., study size, PICOS, follow-up period) and provide the citations. | 12 |
| Risk of bias within studies | 19 | Present data on risk of bias of each study and, if available, any outcome level assessment (see item 12). | N/A |
| Results of individual studies | 20 | For all outcomes considered (benefits or harms), present, for each study: (a) simple summary data for each intervention group (b) effect estimates and confidence intervals, ideally with a forest plot. | 12 |
| Synthesis of results | 21 | Present results of each meta-analysis done, including confidence intervals and measures of consistency. | N/A |
| Risk of bias across studies | 22 | Present results of any assessment of risk of bias across studies (see Item 15). | N/A |
| Additional analysis | 23 | Give results of additional analyses, if done (e.g., sensitivity or subgroup analyses, meta-regression [see Item 16]). | N/A |
| **DISCUSSION** | | |  |
| Summary of evidence | 24 | Summarize the main findings including the strength of evidence for each main outcome; consider their relevance to key groups (e.g., healthcare providers, users, and policy makers). | 26 |
| Limitations | 25 | Discuss limitations at study and outcome level (e.g., risk of bias), and at review-level (e.g., incomplete retrieval of identified research, reporting bias). | 27 |
| Conclusions | 26 | Provide a general interpretation of the results in the context of other evidence, and implications for future research. | 31-32 |
| **FUNDING** | | |  |
| Funding | 27 | Describe sources of funding for the systematic review and other support (e.g., supply of data); role of funders for the systematic review. | 32 |

*From:*  Moher D, Liberati A, Tetzlaff J, Altman DG, The PRISMA Group (2009). Preferred Reporting Items for Systematic Reviews and Meta-Analyses: The PRISMA Statement. PLoS Med 6(7): e1000097. doi:10.1371/journal.pmed1000097. For more information, visit: **www.prisma-statement.org**.

S2 Table. Full-text articles excluded from (a) and included in (b) the present study. Reasons for exclusion, primarily around inability to standardise contamination data given the information presented, are given. Main categories around field or laboratory-based studies for included papers have been provided.

### Publications excluded

| Reference | Reason |
| --- | --- |
| Akhbarizadeh et al. [1] | Inability to convert units |
| Allen et al. [2] | Inability to convert units |
| Asmonaite et al. [3] | Does not mention ingestion |
| Barboza et al. [4] | No quantification |
| Barboza et al. [5] | No quantification |
| Barboza et al. [6] | No quantification |
| Beiras and Tato [7] | No quantification |
| Beiras et al. [8] | No quantification |
| Bessa et al. [9] | Not enough information |
| Besseling et al. [10] | No quantification |
| Bjorndal et al. [11] | No size measurements |
| Bordbar et al. [12] | Percentage only |
| Bour et al. [13] | Percentage only |
| Boyle and Limpus [14] | No size measurements |
| Brate et al. [15] | Not enough information |
| Browne et al. [16] | No quantification |
| Budimir et al. [17] | Not enough information |
| Bussolaro et al. [18] | Nanoplastics only |
| Cannon et al. [19] | Percentage only |
| Carreras-Colom et al. [20] | Percentage only |
| Caruso et al. [21] | No quantification |
| Chagnon et al. [22] | Not microplastics |
| Chapron et al. [23] | No quantification |
| Cole and Galloway [24] | No quantification |
| Cole et al. [25] | No quantification |
| Compa et al. [26] | Not enough information |
| Courtene-Jones et al. [27] | Inability to convert units |
| Courtene-Jones et al. [28] | Inability to convert units |
| Courtene-Jones et al. [29] | No trophic level |
| Cunha et al. [30] | No quantification |
| Dantas et al. [31] | Percentage only |
| Davarpanah and Guilhermino [32] | No quantification |
| Davison and Asch [33] | No species-specific information |
| Dawson et al. [34] | No quantification |
| Dawson et al. [35] | Inability to convert units |
| de Orte et al. [36] | Inability to convert units |
| de Sa et al. [37] | Inability to convert units |
| Detree and Gallardo-Escarate [38] | No quantification |
| Ding et al. [39] | Range only |
| Ding et al. [40] | MPs not from entire sample size |
| Duncan et al. [41] | No species-specific information |
| Egbeocha et al. [42] | Review paper |
| Espinosa et al. [43] | No quantification |
| Fang et al. [44] | Range only |
| Fernandez and Albentosa [45] | Inability to convert units |
| Ferreira et al. [46] | Percentage only |
| Ferreira et al. [47] | No quantification |
| Ferreira et al. [48] | No species-specific information |
| Floren and Shugart [49] | Focus on marine birds |
| Fonte et al. [50] | No quantification |
| Fossi et al. [51] | MPs not investigated |
| Fossi et al. [52] | Quantity not given |
| Fossi et al. [53] | Quantity not given |
| Franzellitti et al. [54] | No quantification |
| Galgani et al. [55] | No quantification |
| Gardon et al. [56] | No quantification |
| Garrido et al. [57] | No quantification |
| Gaspar et al. [58] | No quantification |
| Gassel and Rochman [59] | Quantity not given |
| Gebhardt and Forster [60] | No quantification |
| Goncalves et al. [61] | No quantification |
| Granby et al. [62] | No quantification |
| Green [63] | No quantification |
| Green et al. [64] | No quantification |
| Green et al. [65] | No quantification |
| Green et al. [66] | No quantification |
| Guebert-Bartholo et al. [67] | Categories only |
| Gutow et al. [68] | No quantification |
| Gutow et al. [69] | No quantification |
| Guven et al. [70] | No quantification |
| Hall et al. [71] | Inability to convert units |
| Halstead et al. [72] | Percentage only |
| Hankins et al. [73] | Inability to convert units |
| Hermabessiere et al. [74] | Method development only |
| Hermsen et al. [75] | No species-specific information |
| Horn et al. [76] | Not enough information |
| Jacob et al. [77] | No quantification |
| Jamieson et al. [78] | No trophic level |
| Jeong et al. [79] | No quantification |
| Jung et al. [80] | No species-specific information |
| Jung et al. [81] | Method development only |
| Karami et al. [82] | Not enough information |
| Khan and Prezant [83] | Inability to convert units |
| Khan et al. [84] | No quantification |
| Kolandhasamy et al. [85] | Not enough information |
| Kuhn et al. [86] | Method development only |
| Kumar et al. [87] | Percentage only |
| La Beur et al. [88] | Not enough information |
| Leung and Chan [89] | No quantification |
| Li et al. [90] | No quantification |
| Li et al. [91] | Range only |
| Liboiron et al. [92] | Not only microplastics |
| Liboiron et al. [93] | Not only microplastics |
| Lo and Chan [94] | No quantification |
| Long et al. [95] | No quantification |
| Luan et al. [96] | No quantification |
| Luis et al. [97] | No quantification |
| M'Rabet et al. [98] | No quantification |
| Macali et al. [99] | Quantity not given |
| Mao et al. [100] | No quantification |
| Martinez-Gomez et al. [101] | No quantification |
| Mathalon and Hill [102] | No ingestion (fecal casts) |
| Mecozzi et al. [103] | Method development only |
| Messinetti et al. [104] | Inability to convert units |
| Messinetti et al. [105] | No quantification |
| Miranda and de Carvalho-Souza [106] | Range only |
| Naidoo et al. [107] | Percentage only |
| Naidu [108] | Quantity not given |
| Naidu et al. [109] | Quantity not given |
| Naji et al. [110] | Not enough information |
| Nel and Froneman [111] | No ingestion (tube structures) |
| Nobre et al. [112] | No quantification |
| Okubo et al. [113] | No quantification |
| Oliviero et al. [114] | No quantification |
| Ory et al. [115] | Percentage only |
| Peda et al. [116] | No quantification |
| Peters et al. [117] | No species-specific information |
| Pham et al. [118] | Not only microplastics |
| Phuong et al. [119] | Method development only |
| Pozo et al. [120] | Percentage only |
| Prata et al. [121] | No quantification |
| Rebolledo et al. [122] | Not only microplastics |
| Reichert et al. [123] | Area investigated not mentioned |
| Reichert et al. [124] | No quantification |
| Remy et al. [125] | Not microplastics |
| Renzi et al. [126] | Not only microplastics |
| Renzi et al. [127] | Not enough information |
| Ribeiro et al. [128] | No quantification |
| Rist et al. [129] | No quantification |
| Rivera-Hernandez et al. [130] | No quantification |
| Roch and Brinker [131] | MPs not from entire sample size |
| Rochman et al. [132] | MPs not investigated |
| Rochman et al. [133] | Not only microplastics |
| Romeo et al. [134] | Not only microplastics |
| Rosas-Luis [135] | Not only microplastics |
| Santana et al. [136] | Percentage only |
| Santana et al. [137] | No quantification |
| Santana et al. [138] | Inability to convert units |
| Savoca et al. [139] | Percentage only |
| Schuyler et al. [140] | No size measurements |
| Schuyler et al. [141] | Review paper |
| Seuront [142] | No quantification |
| Silva et al. [143] | No quantification |
| Sjollema et al. [144] | No quantification |
| Smith [145] | Not only microplastics |
| Sun et al. [146] | Inability to convert units |
| Sun et al. [147] | Inability to convert units |
| Sun et al. [148] | No species-specific information |
| Sun et al. [149] | No quantification |
| Sundbaek et al. [150] | Inability to convert units |
| Sussarellu et al. [151] | Inability to convert units |
| Syakti et al. [152] | Inability to convert units |
| Syberg et al. [153] | No quantification |
| Tang et al. [154] | No quantification |
| Thiagarajan et al. [155] | No quantification |
| Thushari et al. [156] | Inability to convert units |
| Tosetto et al. [157] | No quantification |
| Van Cauwenberghe and Janssen [158] | Inability to convert units |
| van Franeker et al. [159] | Not only microplastics |
| von Moos et al. [160] | No quantification |
| Wang et al. [161] | Method development only |
| Wang et al. [162] | No quantification |
| Wang et al. [163] | Range only |
| Watts et al. [164] | No quantification |
| Watts et al. [165] | No quantification |
| Welden and Cowie [166] | Percentage only |
| Welden and Cowie [167] | No quantification |
| Wesch et al. [168] | Not only microplastics |
| White et al. [169] | No species-specific information |
| Wieczorek et al. [170] | Not enough information |
| Wieczorek et al. [171] | No quantification |
| Wilcox et al. [172] | Not only microplastics |
| Wojcik-Fudalewska et al. [173] | Not microplastics |
| Woods et al. [174] | Inability to convert units |
| Wright et al. [175] | No quantification |
| Yu et al. [176] | Inability to convert units |
| Zhang et al. [177] | No quantification |
| Zhu et al. [178] | No species-specific information |
| Zhu et al. [179] | No quantification |

### Publications included

| **Reference** | Category |
| --- | --- |
| **Abbasi et al. [180]** | Field MP Uptake |
| **Alomar and Deudero [181]** | Field MP Uptake |
| **Alomar et al. [182]** | Field MP Uptake |
| **Arias et al. [183]** | Field MP Uptake |
| **Avio et al. [184]** | Field MP Uptake |
| **Avio et al. [185]** | Field MP Uptake |
| **Avio et al. [186]** | Lab Chemical |
| **Azad et al. [187]** | Field MP Uptake |
| **Baalkhuyur et al. [188]** | Field MP Uptake |
| **Bellas et al. [189]** | Field MP Uptake |
| **Bernardini et al. [190]** | Field MP Uptake |
| **Besseling et al. [191]** | Field MP Uptake |
| **Besseling et al. [192]** | Lab MP Uptake |
| **Birnstiel et al. [193]** | Field MP Uptake |
| **Boerger et al. [194]** | Field MP Uptake |
| **Bonello et al. [195]** | Field MP Uptake |
| **Bour et al. [196]** | Lab MP Uptake |
| **Brate et al. [197]** | Field MP Uptake |
| **Brennecke et al. [198]** | Lab MP Uptake |
| **Capolupo et al. [199]** | Lab MP Uptake |
| **Cardozo et al. [200]** | Field MP Uptake |
| **Caron et al. [201]** | Field MP Uptake |
| **Catarino et al. [202]** | Field MP Uptake |
| **Catarino et al. [203]** | Field MP Uptake |
| **Cheung et al. [204]** | Field MP Uptake |
| **Cho et al. [205]** | Field MP Uptake |
| **Chua et al. [206]** | Lab MP Uptake & Lab Chemical |
| **Cole et al. [25]** | Lab Trophic Transfer |
| **Cole et al. [207]** | Lab MP Uptake |
| **Collard et al. [208]** | Field MP Uptake |
| **Collard et al. [209]** | Field MP Uptake |
| **Collicutt et al. [210]** | Field MP Uptake |
| **Courtene-Jones et al. [29]** | Field MP Uptake |
| **Critchell and Hoogenboom [211]** | Lab MP Uptake |
| **Davidson and Dudas [212]** | Field MP Uptake |
| **Desforges et al. [213]** | Field MP Uptake |
| **Devriese et al. [214]** | Field MP Uptake |
| **Devriese et al. [215]** | Lab Chemical |
| **Digka et al. [216]** | Field MP Uptake |
| **Donohue et al. [217]** | Field MP Uptake |
| **Duncan et al. [41]** | Field MP Uptake |
| **Farrell and Nelson [218]** | Lab Trophic Transfer |
| **Ferreira et al. [219]** | Field MP Uptake |
| **Foekema et al. [220]** | Field MP Uptake |
| **Garnier et al. [221]** | Field MP Uptake |
| **Giani et al. [222]** | Field MP Uptake |
| **Goldstein and Goodwin [223]** | Field MP Uptake |
| **Goss et al. [224]** | Field MP Uptake |
| **Gray and Weinstein [225]** | Lab MP Uptake |
| **Guven et al. [226]** | Field MP Uptake |
| **Hamer et al. [227]** | Lab MP Uptake |
| **Hermabessiere et al. [228]** | Field MP Uptake & Field Chemical |
| **Hernandez-Gonzalez et al. [229]** | Field MP Uptake |
| **Herrera et al. [230]** | Field MP Uptake |
| **Jabeen et al. [231]** | Field MP Uptake |
| **Jeong et al. [232]** | Lab MP Uptake |
| **Jovanovic et al. [233]** | Lab MP Uptake |
| **Kaposi et al. [234]** | Lab MP Uptake |
| **Karami et al. [235]** | Field MP Uptake |
| **Karlsson et al. [236]** | Field MP Uptake |
| **Kosore et al. [237]** | Field MP Uptake |
| **Kroon et al. [238]** | Field MP Uptake |
| **Kuhn et al. [239]** | Field MP Uptake |
| **Li et al. [240]** | Field MP Uptake |
| **Li et al. [241]** | Field MP Uptake |
| **Li et al. [242]** | Field MP Uptake |
| **Lourenço et al. [243]** | Field MP Uptake |
| **Lusher et al. [244]** | Field MP Uptake |
| **Lusher et al. [245]** | Field MP Uptake |
| **Lusher et al. [246]** | Field MP Uptake |
| **Magara et al. [247]** | Lab Chemical |
| **Mancuso et al. [248]** | Field MP Uptake |
| **Markic et al. [249]** | Field MP Uptake |
| **Mazurais et al. [250]** | Lab MP Uptake |
| **McGoran et al. [251]** | Field MP Uptake |
| **Mohsen et al. [252]** | Field MP Uptake |
| **Morgana et al. [253]** | Field MP Uptake |
| **Murphy et al. [254]** | Field MP Uptake |
| **Nadal et al. [255]** | Field MP Uptake |
| **Naidoo et al. [256]** | Field MP Uptake |
| **Nelms et al. [257]** | Field MP Uptake |
| **Nelms et al. [258]** | Field MP Uptake |
| **Neves et al. [259]** | Field MP Uptake |
| **O'Donovan et al. [260]** | Lab Chemical |
| **Ory et al. [261]** | Lab MP Uptake |
| **Paul-Pont et al. [262]** | Lab Chemical |
| **Pegado et al. [263]** | Field MP Uptake |
| **Pellini et al. [264]** | Field MP Uptake |
| **Perez-Venegas et al. [265]** | Field MP Uptake |
| **Peters et al. [266]** | Field MP Uptake |
| **Phuong et al. [267]** | Field MP Uptake |
| **Pittura et al. [268]** | Lab Chemical |
| **Porter et al. [269]** | Lab MP Uptake |
| **Procter et al. [270]** | Lab MP Uptake |
| **Qu et al. [271]** | Lab MP Uptake |
| **Romeo et al. [272]** | Field MP Uptake |
| **Rummel et al. [273]** | Field MP Uptake |
| **Setala et al. [274]** | Lab MP Uptake |
| **Silva et al. [275]** | Field MP Uptake |
| **Steer et al. [276]** | Field MP Uptake |
| **Su et al. [277]** | Field MP Uptake |
| **Sun et al. [278]** | Field MP Uptake |
| **Tanaka and Takada [279]** | Field MP Uptake |
| **Taylor et al. [280]** | Field MP Uptake |
| **Teng et al. [281]** | Field MP Uptake |
| **Vandermeersch et al. [282]** | Field MP Uptake |
| **Vendel et al. [283]** | Field MP Uptake |
| **Vered et al. [284]** | Field MP Uptake & Field Chemical |
| **Vroom et al. [285]** | Lab MP Uptake |
| **Wagner et al. [286]** | Field MP Uptake |
| **Waite et al. [287]** | Field MP Uptake |
| **Wang et al. [288]** | Field MP Uptake |
| **Watts et al. [289]** | Lab Trophic Transfer |
| **Xiong et al. [290]** | Field MP Uptake |
| **Xu et al. [291]** | Lab MP Uptake |
| **Zhao et al. [292]** | Field MP Uptake |
| **Zhu et al. [293]** | Field MP Uptake |

S3 Table. Bioaccumulation of (a) microplastics (MPs) for marine organisms collected in situ and (b) relevant calculations for standardisation of MP contamination data. Microplastic concentrations per individual (MPs individual^-1^, i.e. body-burden) were estimated for each species, with concentrations representative of MPs per number of total organisms in the sample size for a particular species, rather than taken from only the number of organisms that exhibited MP contamination. Also included are data calculations transforming MPs gram weight^-1^ to MPs individual^-1^, for *in situ* marine organisms exhibiting MP contamination.

### MP contamination for marine organisms *in situ*

| **Trophic Level** | **Class** | **Family** | **Common Name** | ***Species*** | **Location** | **Area Investigated** | **n** | **MP Individual^-1^** | | **Reference** |
| --- | --- | --- | --- | --- | --- | --- | --- | --- | --- | --- |
| **1** | Magnoliopsida | Hydrocharitaceae | Seagrass | *Thalassia testudinum* | Belize | WO | 16 | 4.56 | | Goss et al. [224] |
| **2** | Actinopterygii | Acanthuridae | Surgeonfish | *Acanthurus lineatus* | South Pacific | GI tract | 24 | 0.25 | | Markic et al. [249] |
|  |  |  |  | *Acanthurus sohal* | Red Sea | GI tract | 3 | 0 | | Baalkhuyur et al. [188] |
|  |  |  |  | *Ctenochaetus striatus* | South Pacific | GI tract | 56 | 0.30 | | Markic et al. [249] |
|  |  | Gobiidae | Mudskipper | *Boleophthalmus pectinirostris* | China | GI tract | 9 | 5.30 | | Su et al. [277] |
|  |  | Hemiramphidae | Halfbeak | *Hyporhamphus unifasciatus* | Brazil | GI tract | 209 | 0.14 | | Vendel et al. [283] |
|  |  | Kyphosidae | Chub | *Kyphosus sandwicensis* | South Pacific | GI tract | 39 | 4.72 | | Markic et al. [249] |
|  |  | Mugilidae | Mullet | *Mugil curema* | Brazil | GI tract | 186 | 0.01 | | Vendel et al. [283] |
|  |  |  |  | *Mugil curvidens* | Brazil | GI tract | 9 | 0 | | Vendel et al. [283] |
|  |  |  |  | *Mugil hospes* | Brazil | GI tract | 28 | 0 | | Vendel et al. [283] |
|  |  |  |  | *Mugil incilis* | Brazil | GI tract | 5 | 0 | | Vendel et al. [283] |
|  |  |  |  | *Mugil liza* | Brazil | GI tract | 2 | 0 | | Vendel et al. [283] |
|  |  | Scaridae | Parrotfish | *Nicholsina usta* | Brazil | GI tract | 2 | 0 | | Vendel et al. [283] |
|  |  |  |  | *Scarus niger* | South Pacific | GI tract | 30 | 0.27 | | Markic et al. [249] |
|  |  |  |  | *Scarus oviceps* | South Pacific | GI tract | 45 | 0.31 | | Markic et al. [249] |
|  |  |  |  | *Scarus psittacus* | South Pacific | GI tract | 30 | 0.17 | | Markic et al. [249] |
|  |  | Siganidae | Rabbitfish | *Siganus luridus* | Turkey | GI tract | 15 | 3.13 | | Guven et al. [226] |
|  |  |  |  | *Siganus punctatus* | South Pacific | GI tract | 29 | 0.24 | | Markic et al. [249] |
|  |  |  |  | *Siganus spp.* | French Polynesia | GI tract | 33 | 0.15 | | Garnier et al. [221] |
|  | Bivalvia | Acrcidae | Clam | *Senilia senilis* | Atlantic Ocean | Soft tissue | 20 | 1.00 | | Lourenço et al. [243] |
|  |  |  |  | *Scapharca subcrenata* | China | Soft tissue | 6 | 46.52 | | Li et al. [240] |
|  |  |  |  | *Tegillarca granosa* | China | Soft tissue | 18 | 5.16 | | Li et al. [240] |
|  |  | Cardiidae | Clam | *Cerastoderma edule* | Atlantic Ocean | Soft tissue | 10 | 4.30 | | Lourenço et al. [243] |
|  |  |  |  |  | France | Soft tissue | 50 | 5.72 | | Hermabessiere et al. [228] |
|  |  | Mytilidae | Mussel | *Modiolus modiolus* | UK | Soft tissue | 6 | 3.50 | | Catarino et al. [203] |
|  |  |  |  | *Mytilus edulis* | China | Soft tissue | 1100 | 3.94 | | Li et al. [241] |
|  |  |  |  |  | Europe | Whole animal | 125 | 0.73 | | Vandermeersch et al. [282] |
|  |  |  |  |  | France | Soft tissue | 50 | 5.88 | | Hermabessiere et al. [228] |
|  |  |  |  |  | France | Soft tissue | 120 | 0.60 | | Phuong et al. [267] |
|  |  |  |  |  | North Sea | Soft tissue | 17 | 1.23 | | Karlsson et al. [236] |
|  |  |  |  |  | South Korea | Soft tissue | 60 | 0.68 | | Cho et al. [205] |
|  |  |  |  |  | UK | Soft tissue | 18 | 4.20 | | Catarino et al. [202] |
|  |  |  |  |  | UK | Soft tissue | 162 | 6.69 | | Li et al. [242] |
|  |  |  |  |  | USA | GI tract | 35 | 0.37 | | Zhao et al. [292] |
|  |  |  |  | *Mytilus spp.* | Norway | Soft tissue | 332 | 1.50 | | Brate et al. [197] |
|  |  |  |  |  | UK | Soft tissue | 36 | 3.20 | | Catarino et al. [203] |
|  |  | Nuculidae | Clam | *Acila mirabilis* | China | Soft tissue | 20 | 5.50 | | Wang et al. [288] |
|  |  | Ostreidae | Oyster | *Alectryonella plicatula* | China | Soft tissue | 18 | 10.12 | | Li et al. [240] |
|  |  |  |  | *Crassostrea gigas* | France | Soft tissue | 60 | 2.10 | | Phuong et al. [267] |
|  |  |  |  |  | Italy | Soft tissue | 100 | 0.18 | | Bonello et al. [195] |
|  |  |  |  |  | South Korea | Soft tissue | 60 | 0.77 | | Cho et al. [205] |
|  |  |  |  | *Crassostrea spp.* | China | Soft tissue | 306 | 3.98 | | Teng et al. [281] |
|  |  |  |  | *Crassostrea virginica* | USA | Soft tissue | 90 | 16.47 | | Waite et al. [287] |
|  |  | Pectinidae | Scallop | *Mizuhopecten yessoensis* | China | Soft tissue | 6 | 57.04 | | Li et al. [240] |
|  |  |  |  |  | South Korea | Soft tissue | 60 | 1.21 | | Cho et al. [205] |
|  |  | Pharidae | Clam | *Sinonovacula constricta* | China | Soft tissue | 6 | 15.06 | | Li et al. [240] |
|  |  | Semelidae | Clam | *Scrobicularia plana* | Atlantic Ocean | Soft tissue | 10 | 3.30 | | Lourenço et al. [243] |
|  |  | Veneridae | Clam | *Cyclina sinensis* | China | Soft tissue | 30 | 4.76 | | Li et al. [240] |
|  |  |  |  | *Meretrix lusoria* | China | Soft tissue | 18 | 9.79 | | Li et al. [240] |
|  |  |  |  | *Pelecyora isocardia* | Atlantic Ocean | Soft tissue | 20 | 1.50 | | Lourenço et al. [243] |
|  |  |  |  | *Ruditapes philippinarum* | Canada | Soft tissue | 54 | 9.00 ^a^ | | Davidson and Dudas [212] |
|  |  |  |  |  | China | Soft tissue | 24 | 5.53 | | Li et al. [240] |
|  |  |  |  |  | South Korea | Soft tissue | 60 | 1.15 | | Cho et al. [205] |
|  | Gastropoda | Littorinidae | Snail | *Littorina littorea* | North Sea | Soft tissue | 28 | 1.42 | | Karlsson et al. [236] |
|  | Hexanauplia | Calanidae | Copepod | *Neocalanus cristatus* | Pacific Ocean | Whole animal | 960 | 0.03 | | Desforges et al. [213] |
|  |  |  |  | *Copepoda spp.* | Indian Ocean | WO | 110 | 0.33 | | Kosore et al. [237] |
|  | Malacostraca | - | Isopod | *Isopoda spp.* | North Sea | Soft tissue | 16 | 0.96 | | Karlsson et al. [236] |
|  | Polychaeta | Nereidae | Worm | *Hediste diversicolor* | Atlantic Ocean | Soft tissue | 10 | 2.70 | | Lourenço et al. [243] |
|  |  | Spionidae | Worm | *Scolelepis squamata* | Atlantic Ocean | Soft tissue | 8 | 0.60 | | Lourenço et al. [243] |
| **2.1** | Actinopterygii | Engraulidae | Anchovy | *Cetengraulis edentulus* | Brazil | GI tract | 5 | 0 | | Vendel et al. [283] |
|  |  | Kyphosidae | Chub | *Girella tricuspidata* | South Pacific | GI tract | 20 | 4.15 | | Markic et al. [249] |
| **2.2** | Actinopterygii | Acanthuridae | Unicornfish | *Naso unicornis* | South Pacific | GI tract | 30 | 0.23 | | Markic et al. [249] |
|  |  |  |  |  | Red Sea | GI tract | 2 | 0 | | Baalkhuyur et al. [188] |
|  |  | Mugilidae | Mullet | *Ellochelon vaigiensis* | South Pacific | GI tract | 33 | 2.06 | | Markic et al. [249] |
|  | Ascidiacea | Pyuridae | Ascidian | *Herdmania momus* | Isreal | Soft tissue | 15 | 1.78 | | Vered et al. [284] |
| **2.3** | Actinopterygii | Acanthuridae | Unicornfish | *Naso lituratus* | South Pacific | GI tract | 28 | 0.25 | | Markic et al. [249] |
|  |  | Poeciliidae | Guppy | *Poecilia vivipara* | Brazil | GI tract | 75 | 0.09 | | Vendel et al. [283] |
| **2.32** | Malacostraca | Euphausiidae | Krill | *Euphausia pacifica* | Pacific Ocean | Whole animal | 413 | 0.06 | | Desforges et al. [213] |
| **2.37** | Holothuroidea | Stichopodidae | Sea Cucumber | *Apostichopus japonicus* | China | GI tract | 200 | 10.15 | | Mohsen et al. [252] |
|  |  | - | Sea Cucumber | *Holothurian spp.* | Indian Ocean | All internal cavities | 2 | 3.00 | | Taylor et al. [280] |
| **2.38** | Reptilia | Cheloniidae | Green Sea Turtle | *Chelonia mydas* | Australia | GI tract | 2 | 3.5 | | Caron et al. [201] |
|  |  |  |  |  | Global | GI tract | 51 | 1.09 | | Duncan et al. [294] |
| **2.4** | Actinopterygii | Gerreidae | Mojarras | *Diapterus auratus* | Brazil | GI tract | 29 | 0.97 | | Vendel et al. [283] |
| **2.48** | Bivalvia | Mytilidae | Mussel | *Perna perna* | Brazil | Soft tissue | 10 | 31.20 | | Birnstiel et al. [193] |
| **2.5** | Actinopterygii | Mugilidae | Mullet | *Liza haematocheila* | China | GI tract | 18 | 3.30 | | Jabeen et al. [231] |
|  |  |  |  |  | China | GI tract | 17 | 1.20 | | Su et al. [277] |
|  |  |  |  | *Mugil cephalus* | China | GI tract | 60 | 2.25 | | Cheung et al. [204] |
|  |  |  |  |  | China | GI tract | 18 | 3.10 | | Jabeen et al. [231] |
|  |  |  |  |  | South Africa | GI tract | 70 | 3.80 | | Naidoo et al. [256] |
|  |  |  |  |  | South Pacific | GI tract | 22 | 0.27 | | Markic et al. [249] |
| **2.58** | Maxillopoda | Lepadidae | Barnacle | *Lepas spp.* | Pacific Ocean | GI tract | 385 | 1.35 | | Goldstein and Goodwin [223] |
| **2.6** | Actinopterygii | Clupeidae | Sardine | *Sardinella albella* | Thailand | Stomach | 14 | 2.30 | | Azad et al. [187] |
| **2.69** | Bivalvia | Mytilidae | Mussel | *Mytilus galloprovincialis* | China | Soft tissue | 18 | 4.48 | | Li et al. [240] |
|  |  |  |  |  | Europe | Whole animal | 300 | 0.76 | | Vandermeersch et al. [282] |
|  |  |  |  |  | Ionian Sea | Soft tissue | 80 | 0.85 | | Digka et al. [216] |
|  |  |  |  |  | Italy | Soft tissue | 26 | 1.77 | | Avio et al. [185] |
| **2.7** | Actinopterygii | Mugilidae | Mullet | *Planiliza subviridis* | Malaysia | Flesh Viscera and gills | 30 | 0.53 | | Karami et al. [235] |
|  |  | Pomacanthidae | Angelfish | *Pygoplites diacanthus* | Red Sea | GI tract | 5 | 0 | | Baalkhuyur et al. [188] |
|  |  | Pomacentridae | Damselfish | *Abudefduf sexfasciatus* | Red Sea | GI tract | 5 | 0.20 | | Baalkhuyur et al. [188] |
|  |  | Scombridae | Mackerel | *Rastrelliger brachysoma* | Thailand | Stomach | 3 | 1.00 | | Azad et al. [187] |
| **2.71** | Malacostraca | Panopeidae | Mud crab | *Panopeus herbstii* | USA | Soft tissue | 90 | 21.99 | | Waite et al. [287] |
| **2.8** | Actinopterygii | Acanthuridae | Surgeonfish | *Acanthurus gahhm* | Red Sea | GI tract | 10 | 0.10 | | Baalkhuyur et al. [188] |
|  |  | Clupeidae | Shad | *Anodontostoma chacunda* | Thailand | Stomach | 14 | 2.00 | | Azad et al. [187] |
|  |  | Mugilidae | Mullet | *Liza aurata* | Turkey | GI tract | 39 | 3.26 | | Guven et al. [226] |
|  |  | Pomacentridae | Damselfish | *Dascyllus trimaculatus* | Red Sea | GI tract | 2 | 0 | | Baalkhuyur et al. [188] |
|  |  | Sparidae | Bogue | *Boops boops* | Mediterranean | GI tract | 337 | 3.75 | | Nadal et al. [255] |
|  |  |  |  |  | Portugal | Stomach | 32 | 0.09 | | Neves et al. [259] |
| **2.9** | Actinopterygii | Clupeidae | Sardine | *Sardinella gibbosa* | Thailand | Stomach | 3 | 0.30 | | Azad et al. [187] |
|  |  |  |  | *Sardinella jussieu* | Thailand | Stomach | 8 | 1.30 | | Azad et al. [187] |
|  |  | Engraulidae | Anchovy | *Anchoa januaria* | Brazil | GI tract | 194 | 0.15 | | Vendel et al. [283] |
|  |  | Leiognathidae | Ponyfish | *Eubleekeria splendens* | Thailand | Stomach | 10 | 1.00 | | Azad et al. [187] |
|  |  |  |  | *Photopectoralis bindus* | China | GI tract | 18 | 4.10 | | Jabeen et al. [231] |
| **2.92** | Malacostraca | Penaeidae | Prawn | *Penaeus semisulcatus* | Persian Gulf | WO | 12 | 7.80 | | Abbasi et al. [180] |
| **3** | Actinopterygii | Belonidae | Needlefish | *Strongylura marina* | Brazil | GI tract | 4 | 0 | | Vendel et al. [283] |
|  |  | Engraulidae | Anchovy | *Coilia nasus* | China | GI tract | 18 | 4.00 | | Jabeen et al. [231] |
|  |  |  |  |  | China | GI tract | 36 | 0.69 | | Su et al. [277] |
|  |  | Gerreidae | Mojarras | *Diapterus rhombeus* | Brazil | GI tract | 31 | 0.03 | | Vendel et al. [283] |
|  |  | Hemiramphidae | Halfbeak | *Hyporhamphus roberti roberti* | Brazil | GI tract | 35 | 0.03 | | Vendel et al. [283] |
|  |  | Myctophidae | Lanternfish | *Benthosema glaciale* | Atlantic Ocean | GI tract | 27 | 0.33 | | Lusher et al. [246] |
|  |  |  |  | *Myctophidae spp.* | Global | Stomach | 13 | 0.38 | | Wagner et al. [286] |
|  |  | Sternoptychidae | Hatchetfish | *Maurolicus muelleri* | Atlantic Ocean | GI tract | 282 | 0.03 | | Lusher et al. [246] |
|  | Anthozoa | Actiniidae | Anenome | *Actinia equina* | North Sea | Soft tissue | 7 | 0.41 | | Karlsson et al. [236] |
|  | Gastropoda | Turritellidae | Snail | *Turritellidae spp.* | North Sea | Soft tissue | 10 | 0.53 | | Karlsson et al. [236] |
|  | Polychaeta | Glyceridae | Worm | *Glycera alba* | Atlantic Ocean | Soft tissue | 1 | 3.00 | | Lourenço et al. [243] |
|  |  | Onuphidae | Worm | *Diopatra neapolitana* | Atlantic Ocean | Soft tissue | 4 | 1.00 | | Lourenço et al. [243] |
| **3.07** | Malacostraca | Varunidae | Crab | *Hemigrapsus sanguineus* | North Sea | Soft tissue | 9 | 0.99 | | Karlsson et al. [236] |
| **3.1** | Actinopterygii | Ammodytidae | Sandlance | *Ammodytes personatus* | China | GI tract | 50 | 0.54 | | Sun et al. [278] |
|  |  | Cepolidae | Bandfish | *Cepola macrophthalma* | UK | GI tract | 62 | 2.25 | | Lusher et al. [244] |
|  |  | Clupeidae | Piquitinga | *Lile piquitinga* | Brazil | GI tract | 2 | 0 | | Vendel et al. [283] |
|  |  |  | Sardine | *Sardina pilchardus* | Ionian Sea | GI tract | 36 | 1.80 | | Digka et al. [216] |
|  |  |  |  |  | Mediterranean | GI tract | 99 | 1.78 | | Avio et al. [184] |
|  |  |  |  |  | North Sea | Stomach | 3 | 3.33 | | Collard et al. [208] |
|  |  |  |  |  | Turkey | GI tract | 7 | 2.14 | | Guven et al. [226] |
|  |  |  | Sprat | *Rhinosardinia bahiensis* | Brazil | GI tract | 179 | 0.19 | | Vendel et al. [283] |
|  |  | Engraulidae | Anchovy | *Anchoviella commersonii* | China | GI tract | 30 | 0.40 | | Sun et al. [278] |
|  |  |  |  | *Engraulis encrasicolus* | Mediterranean | Livers | 10 | 0.90 | | Collard et al. [209] |
|  |  |  |  |  | North Sea | Stomach | 3 | 4.30 | | Collard et al. [208] |
|  |  |  |  | *Engraulis japonicus* | China | GI tract | 80 | 0.39 | | Sun et al. [278] |
|  |  |  |  |  | Japan | GI tract | 64 | 2.30 | | Tanaka and Takada [279] |
|  |  | Gadidae | Cod | *Boreogadus saida* | Artic | Stomach | 72 | 0.03 | | Kuhn et al. [239] |
|  |  |  |  |  | Artic | GI tract | 85 | 0.22 | | Morgana et al. [253] |
|  |  | Lateolabracidae | Seabass | *Lateolabrax japonicus* | China | GI tract | 18 | 2.10 | | Jabeen et al. [231] |
|  |  | Monacanthidae | Filefish | *Meuschenia scaber* | South Pacific | GI tract | 19 | 0.74 | | Markic et al. [249] |
|  |  | Mullidae | Mullet | *Mullus barbatus* | Ionian Sea | GI tract | 25 | 1.50 | | Digka et al. [216] |
|  |  |  |  |  | Mediterranean | GI tract | 11 | 1.57 | | Avio et al. [184] |
|  |  |  |  |  | Mediterranean | GI tract | 132 | 0.21 | | Giani et al. [222] |
|  |  |  |  |  | Spain | GI tract | 128 | 1.75 | | Bellas et al. [189] |
|  |  |  |  |  | Turkey | GI tract | 207 | 1.39 | | Guven et al. [226] |
|  |  | Myctophidae | Lanternfish | *Benthosema pterotum* | Red Sea | GI tract | 10 | 0.10 | | Baalkhuyur et al. [188] |
|  |  |  |  | *Symbolophorus californiensis* | Pacific Ocean | Stomach | 74 | 7.20 | | Boerger et al. [194] |
|  |  | Pholidae | Gunnel | *Pholis fangi* | China | GI tract | 79 | 0.48 | | Sun et al. [278] |
|  |  | Phosichthyidae | Lightfish | *Vinciguerria mabahiss* | Red Sea | GI tract | 10 | 0 | | Baalkhuyur et al. [188] |
|  |  | Sciaenidae | Croaker | *Micropogonias furnieri* | Argentina | GI tract | 20 | 12.10 | | Arias et al. [183] |
|  |  |  |  |  | Brazil | GI tract | 6 | 0 | | Pegado et al. [263] |
|  |  |  |  |  | Brazil | GI tract | 1 | 0 | | Vendel et al. [283] |
|  |  |  | Weakfish | *Cynoscion leiarchus* | Brazil | GI tract | 2 | 1.00 | | Pegado et al. [263] |
|  |  |  |  |  | Brazil | GI tract | 9 | 0 | | Vendel et al. [283] |
|  |  | Sternoptychidae | Dragonfish | *Maurolicus mucronatus* | Red Sea | GI tract | 10 | 0.10 | | Baalkhuyur et al. [188] |
| **3.19** | Malacostraca | Crangonidae | Shrimp | *Crangon affinis* | China | Soft tissue | 10 | 29.40 | | Wang et al. [288] |
|  |  |  |  | *Crangon crangon* | North Sea | Whole animal | 165 | 1.23 | | Devriese et al. [214] |
|  |  |  |  |  | UK | GI tract | 116 | 1.00 | | McGoran et al. [251] |
|  | Reptilia | Cheloniidae | Hawksbill Sea Turtle | *Eretmochelys imbricata* | Pacific | GI tract | 1 | 2 | | Duncan et al. [294] |
| **3.2** | Actinopterygii | Alepocephalidae | Slickhead | *Xenodermichthys copei* | Atlantic Ocean | GI tract | 5 | 1.20 | | Lusher et al. [246] |
|  |  | Atherinopsidae | Silverside | *Atherinella brasiliensis* | Brazil | GI tract | 405 | 0.04 | | Vendel et al. [283] |
|  |  | Callionymidae | Dragonet | *Callionymus planus* | China | GI tract | 18 | 4.80 | | Jabeen et al. [231] |
|  |  | Carangidae | Mackerel | *Trachurus novaezelandiae* | South Pacific | GI tract | 31 | 0.03 | | Markic et al. [249] |
|  |  | Engraulidae | Anchovy | *Coilia mystus* | China | GI tract | 9 | 0.33 | | Su et al. [277] |
|  |  | Gerreidae | Mojarras | *Eucinostomus argenteus* | Brazil | GI tract | 98 | 0.01 | | Vendel et al. [283] |
|  |  | Hemiramphidae | Halfbeak | *Hyporhamphus ihi* | South Pacific | GI tract | 24 | 0 | | Markic et al. [249] |
|  |  | Myctophidae | Lancetfish | *Notoscopelus kroyeri* | Atlantic Ocean | GI tract | 417 | 0.16 | | Lusher et al. [246] |
|  |  |  | Lanternfish | *Hygophum reinhardtii* | Pacific Ocean | Stomach | 47 | 1.30 | | Boerger et al. [194] |
|  |  |  |  | *Loweina interrupta* | Pacific Ocean | Stomach | 27 | 1.00 | | Boerger et al. [194] |
|  |  | Myliobatidae | Stingray | *Rhinoptera bonasus* | Brazil | GI tract | 1 | 0 | | Pegado et al. [263] |
|  |  | Paralepididae | Barracudina | *Arctozenus risso* | Atlantic Ocean | GI tract | 14 | 0.29 | | Lusher et al. [246] |
|  |  | Pleuronectidae | Flounder | *Glyptocephalus cynoglossus* | UK | GI tract | 23 | 1.00 | | McGoran et al. [251] |
|  |  |  |  | *Pleuronectes platessa* | Scotland | GI tract | 62 | 0.89 | | Murphy et al. [254] |
|  |  |  |  | *Pleuronectes platessa* | UK | GI tract | 99 | 2.52 | | McGoran et al. [251] |
|  |  | Scombridae | Mackerel | *Rastrelliger kanagurta* | Malaysia | Flesh Viscera and gills | 30 | 0.10 | | Karami et al. [235] |
|  |  | Soleidae | Sole | *Solea solea* | Adriatic Sea | GI tract | 533 | 8.57 | | Pellini et al. [264] |
|  |  |  |  |  | UK | GI tract | 18 | 1.00 | | McGoran et al. [251] |
|  |  | Zoarcidae | Eelpout | *Enchelyopus elongatus* | China | GI tract | 20 | 0.80 | | Sun et al. [278] |
|  | Elasmobranchii | Narcinidae | Stingray | *Narcine brasiliensis* | Brazil | GI tract | 6 | 0.50 | | Pegado et al. [263] |
| **3.3** | Actinopterygii | Achiridae | Sole | *Achirus declivis* | Brazil | GI tract | 7 | 0.14 | | Vendel et al. [283] |
|  |  |  |  | *Trinectes paulistanus* | Brazil | GI tract | 3 | 0 | | Vendel et al. [283] |
|  |  | Argentinidae | Argentine | *Argentina silus* | Scotland | GI tract | 15 | 0.07 | | Murphy et al. [254] |
|  |  | Ariidae | Sea Catfish | *Sciades herzbergii* | Brazil | GI tract | 57 | 0.07 | | Vendel et al. [283] |
|  |  | Callionymidae | Dragonet | *Callionymus lyra* | UK | GI tract | 50 | 1.80 | | Lusher et al. [244] |
|  |  |  |  | *C. lyra* larvae | UK | GI tract | 86 | 0.02 | | Steer et al. [276] |
|  |  | Carangidae | Mackerel | *Trachurus picturatus* | Portugal | Stomach | 29 | 0.03 | | Neves et al. [259] |
|  |  | Chaetodontidae | Butterflyfish | *Chaetodon austriacus* | Red Sea | GI tract | 10 | 0.10 | | Baalkhuyur et al. [188] |
|  |  | Cottidae | Sculpin | *Triglops nybelini* | Artic | GI tract | 71 | 0.39 | | Morgana et al. [253] |
|  |  | Engraulidae | Anchovy | *Stolephorus waitei* | Malaysia | Flesh Viscera and gills | 30 | 0.03 | | Karami et al. [235] |
|  |  | Gobionellinae | Goby | *Ctenogobius boleosoma* | Brazil | GI tract | 27 | 0.04 | | Vendel et al. [283] |
|  |  | Leiognathidae | Ponyfish | *Aurigequula fasciata* | Thailand | Stomach | 3 | 1.30 | | Azad et al. [187] |
|  |  |  |  | *Leiognathus berbis* | Thailand | Stomach | 8 | 0.90 | | Azad et al. [187] |
|  |  | Pleuronectidae | Flounder | *Platichthys flesus* | North Sea | GI tract | 36 | 0.06 | | Rummel et al. [273] |
|  |  |  |  |  | Scotland | GI tract | 47 | 1.02 | | Murphy et al. [254] |
|  |  |  |  |  | UK | GI tract | 126 | 3.10 | | McGoran et al. [251] |
|  |  | Sciaenidae | Croaker | *Johnius belangerii* | Malaysia | Flesh Viscera and gills | 30 | 0.53 | | Karami et al. [235] |
|  |  | Sciaenidae | Croaker | *Stellifer brasiliensis* | Brazil | GI tract | 1 | 0 | | Vendel et al. [283] |
|  |  | Scombridae | Mackerel | *Scomberomorus brasiliensis* | Brazil | GI tract | 1 | 0 | | Pegado et al. [263] |
|  |  | Sillaginidae | Whiting | *Sillago sihama* | China | GI tract | 18 | 2.80 | | Jabeen et al. [231] |
|  |  |  |  |  | Persian Gulf | WO | 17 | 14.10 | | Abbasi et al. [180] |
|  |  | Soleidae | Sole | *Buglossidium luteum* | UK | GI tract | 50 | 1.25 | | Lusher et al. [244] |
|  |  | Soleidae | Sole | *Microchirus variegatus* | UK | GI tract | 51 | 1.50 | | Lusher et al. [244] |
|  |  |  |  | *M. variegatus* larvae | UK | GI tract | 16 | 0.19 | | Steer et al. [276] |
|  |  | Sparidae | Porgy | *Spondyliosoma cantharus* | Italy | GI tract | 5 | 3.65 | | Avio et al. [185] |
|  |  | Stromateidae | Pomfret | *Pampus argenteus* | China | GI tract | 18 | 3.00 | | Jabeen et al. [231] |
|  |  |  |  |  | China | GI tract | 9 | 1.10 | | Su et al. [277] |
|  |  |  |  |  | China | GI tract | 10 | 0.20 | | Sun et al. [278] |
| **3.38** | Reptilia | Cheloniidae | Olive Ridley Sea Turtle | *Lepidochelys olivacea* | Pacific | GI tract | 1 | 1 | | Duncan et al. [294] |
| **3.39** | Reptilia | Cheloniidae | Flatback Sea Turtle | *Natator depressus* | Pacific | GI tract | 4 | 1.5 | | Duncan et al. [294] |
| **3.4** | Actinopterygii | Atherinopsidae | Silverside | *Atherinella blackburni* | Brazil | GI tract | 2 | 0 | | Vendel et al. [283] |
|  |  | Carangidae | Scad | *Alepes melanoptera* | Thailand | Stomach | 8 | 1.30 | | Azad et al. [187] |
|  |  |  |  | *Decapterus maruadsi* | China | GI tract | 78 | 0.41 | | Sun et al. [278] |
|  |  |  |  |  | South Pacific | GI tract | 25 | 1.56 | | Markic et al. [249] |
|  |  | Cheilodactylidae | Morwong | *Nemadactylus macropterus* | South Pacific | GI tract | 23 | 0.30 | | Markic et al. [249] |
|  |  | Clupeidae | Herring | *Clupea harengus* | North Sea | Stomach | 3 | 4.00 | | Collard et al. [208] |
|  |  |  |  |  | North Sea | GI tract | 566 | 0.03 | | Foekema et al. [220] |
|  |  | Engraulidae | Anchovy | *Anchoa marinii* | Brazil | GI tract | 22 | 0.04 | | Vendel et al. [283] |
|  |  |  |  | *Anchoa tricolor* | Brazil | GI tract | 6 | 0 | | Vendel et al. [283] |
|  |  |  |  | *Anchovia clupeoides* | Brazil | GI tract | 10 | 0.10 | | Vendel et al. [283] |
|  |  | Gerreidae | Mojarras | *Eugerres brasilianus* | Brazil | GI tract | 64 | 0.04 | | Vendel et al. [283] |
|  |  |  |  | *Eucinostomus melanopterus* | Brazil | GI tract | 21 | 0 | | Vendel et al. [283] |
|  |  | Gobiidae | Goby | *Ctenogobius stigmaticus* | Brazil | GI tract | 3 | 0 | | Vendel et al. [283] |
|  |  | Haemulidae | Grunt | *Orthopristis chrysoptera* | Gulf of Mexico | Stomach | 157 | 0.54 | | Peters et al. [266] |
|  |  | Hemiramphidae | Halfbeak | *Hyporhamphus intermedius* | China | GI tract | 18 | 3.70 | | Jabeen et al. [231] |
|  |  | Malacanthidae | Tilefish | *Branchiostegus japonicus* | China | GI tract | 18 | 4.60 | | Jabeen et al. [231] |
|  |  | Monacanthidae | Filefish | *Thamnaconus septentrionalis* | China | GI tract | 18 | 7.20 | | Jabeen et al. [231] |
|  |  |  |  |  | China | GI tract | 9 | 0.67 | | Su et al. [277] |
|  |  | Myctophidae | Lanternfish | *Myctophum aurolanternatum* | Pacific Ocean | Stomach | 462 | 6.00 | | Boerger et al. [194] |
|  |  | Oxudercidae | Goby | *Tridentiger barbatus* | China | GI tract | 8 | 4.50 | | Su et al. [277] |
|  |  | Pleuronectidae | Dab | *Limanda limanda* | North Sea | GI tract | 89 | 0.05 | | Rummel et al. [273] |
|  |  |  |  |  | Scotland | GI tract | 19 | 0.68 | | Murphy et al. [254] |
|  |  |  |  |  | UK | GI tract | 308 | 3.25 | | McGoran et al. [251] |
|  |  | Pleuronectidae | Flounder | *Cleisthenes herzensteini* | China | GI tract | 36 | 0.44 | | Sun et al. [278] |
|  |  | Pristigasteridae | Tardoore | *Opisthopterus tardoore* | Thailand | Stomach | 3 | 2.00 | | Azad et al. [187] |
|  |  | Sciaenidae | Croaker | *Paralonchurus brasiliensis* | Brazil | GI tract | 6 | 0 | | Pegado et al. [263] |
|  |  | Scombridae | Mackerel | *Scomber japonicus* | China | GI tract | 9 | 0.78 | | Su et al. [277] |
|  |  |  |  |  | Portugal | Stomach | 35 | 0.57 | | Neves et al. [259] |
|  |  |  |  |  | Turkey | GI tract | 7 | 6.71 | | Guven et al. [226] |
|  |  | Scorpaeniformes | Poacher | *Agonus cataphractus* | UK | GI tract | 3 | 0 | | McGoran et al. [251] |
|  |  | Serranidae | Seabass | *Serranus cabrilla* | Turkey | GI tract | 6 | 1.50 | | Guven et al. [226] |
|  |  | Sparidae | Seabream | *Lithognathus mormyrus* | Turkey | GI tract | 46 | 0.63 | | Guven et al. [226] |
|  |  | Tetradontidae | Pufferfish | *Sphoeroides testudineus* | Brazil | GI tract | 55 | 0.04 | | Vendel et al. [283] |
| **3.5** | Actinopterygii | Achiridae | Sole | *Achirus lineatus* | Brazil | GI tract | 10 | 0.20 | | Vendel et al. [283] |
|  |  | Ariidae | Sea Catfish | *Bagre marinus* | Brazil | GI tract | 4 | 7.75 | | Pegado et al. [263] |
|  |  |  |  | *Cathorops spixii* | Brazil | GI tract | 2 | 1.00 | | Vendel et al. [283] |
|  |  | Carangidae | Scad | *Alepes apercna* | Thailand | Stomach | 3 | 2.00 | | Azad et al. [187] |
|  |  |  |  | *Alepes kleinii* | Thailand | Stomach | 4 | 0.80 | | Azad et al. [187] |
|  |  |  |  | *Decapterus macrosoma* | South Pacific | GI tract | 25 | 0.32 | | Markic et al. [249] |
|  |  | Cynoglossidae | Tonguefish | *Cynoglossus abbreviatus* | China | GI tract | 18 | 6.90 | | Jabeen et al. [231] |
|  |  |  |  |  | Persian Gulf | WO | 11 | 12.00 | | Abbasi et al. [180] |
|  |  | Drepaneidae | Sicklefish | *Drepane longimana* | Thailand | Stomach | 3 | 0.30 | | Azad et al. [187] |
|  |  | Elopidae | Ladyfish | *Elops saurus* | Brazil | GI tract | 1 | 0 | | Vendel et al. [283] |
|  |  | Gobiidae | Goby | *Gobionellus oceanicus* | Brazil | GI tract | 6 | 0 | | Vendel et al. [283] |
|  |  |  |  | *Microgobius meeki* | Brazil | GI tract | 1 | 0 | | Vendel et al. [283] |
|  |  | Haemulidae | Grunt | *Genyatremus luteus* | Brazil | GI tract | 8 | 0 | | Pegado et al. [263] |
|  |  | Holocentridae | Soldierfish | *Myripristis spp.* | French Polynesia | GI tract | 33 | 0.27 | | Garnier et al. [221] |
|  |  | Labridae | Wrasse | *Thalassoma rueppellii* | Red Sea | GI tract | 12 | 0.08 | | Baalkhuyur et al. [188] |
|  |  | Macrouridae | Grenadier | *Coryphaenoides rupestris* | Scotland | GI tract | 5 | 0 | | Murphy et al. [254] |
|  |  | Mullidae | Goatfish | *Upeneus pori* | Turkey | GI tract | 78 | 0.69 | | Guven et al. [226] |
|  |  |  | Mullet | *Mullus surmuletus* | Mediterranean | GI tract | 417 | 0.50 | | Alomar et al. [182] |
|  |  |  |  |  | Portugal | Stomach | 4 | 1.75 | | Neves et al. [259] |
|  |  |  |  |  | Turkey | GI tract | 51 | 1.18 | | Guven et al. [226] |
|  |  | Muraenesocidae | Pike Conger | *Cynoponticus savanna* | Brazil | GI tract | 1 | 0 | | Pegado et al. [263] |
|  |  | Nemichthyidae | Eel | *Nemichthys scolopaceus* | Atlantic Ocean | GI tract | 1 | 1.00 | | Lusher et al. [246] |
|  |  | Nemipteridae | Bream | *Parascolopsis eriomma* | Red Sea | GI tract | 5 | 0.60 | | Baalkhuyur et al. [188] |
|  |  | Paralichthyidae | Flounder | *Citharichthys macrops* | Brazil | GI tract | 7 | 0 | | Vendel et al. [283] |
|  |  |  |  | *Etropus crossotus* | Brazil | GI tract | 1 | 0 | | Vendel et al. [283] |
|  |  | Pholidae | Gunnel | *Pholis gunnellus* | UK | GI tract | 1 | 0 | | McGoran et al. [251] |
|  |  | Sciaenidae | Croaker | *Bairdiella ronchus* | Brazil | GI tract | 4 | 0 | | Pegado et al. [263] |
|  |  |  |  | *Dendrophysa russelii* | Thailand | Stomach | 3 | 0.30 | | Azad et al. [187] |
|  |  |  |  | *Johnius carouna* | Thailand | Stomach | 20 | 3.80 | | Azad et al. [187] |
|  |  |  |  | *Menticirrhus americanus* | Gulf of Mexico | Stomach | 150 | 0.57 | | Peters et al. [266] |
|  |  |  |  |  | Brazil | GI tract | 1 | 0 | | Pegado et al. [263] |
|  |  |  |  |  | Brazil | GI tract | 1 | 0 | | Vendel et al. [283] |
|  |  | Sparidae | Pandora | *Pagellus erythrinus* | Ionian Sea | GI tract | 19 | 1.90 | | Digka et al. [216] |
|  |  |  |  | *Pagellus erythrinus* | Turkey | GI tract | 54 | 0.63 | | Guven et al. [226] |
|  |  |  | Porgy | *Dentex macrophthalmus* | Portugal | Stomach | 1 | 1.00 | | Neves et al. [259] |
|  |  | Terapontidae | Terapon | *Terapon theraps* | Thailand | Stomach | 5 | 0.80 | | Azad et al. [187] |
|  |  | Tetradontidae | Pufferfish | *Sphoeroides greeleyi* | Brazil | GI tract | 31 | 0 | | Vendel et al. [283] |
|  | Elasmobranchii | Dasyatidae | Stingray | *Dasyatis zugei* | Thailand | Stomach | 3 | 0.30 | | Azad et al. [187] |
| **3.53** | Reptilia | Cheloniidae | Kemp’s Ridley Sea Turtle | *Lepidochelys kempii* | Atlantic | GI tract | 10 | | 0.5 | Duncan et al. [294] |
| **3.54** | Reptilia | Cheloniidae | Loggerhead Sea Turtle | *Caretta caretta* | Global | GI tract | 33 | | 2.52 | Duncan et al. [294] |
| **3.6** | Actinopterygii | Anguillidae | Eel | *Anguilla anguilla* | UK | GI tract | 2 | 2.00 | | McGoran et al. [251] |
|  |  |  |  | *A. anguilla* larvae | UK | GI tract | 1 | 1.00 | | Steer et al. [276] |
|  |  | Carangidae | Jack | *Caranx hippos* | Brazil | GI tract | 3 | 30.67 | | Pegado et al. [263] |
|  |  | Cottidae | Sculpin | *Taurulus bubalis* | UK | GI tract | 3 | 2.00 | | McGoran et al. [251] |
|  |  | Cynoglossidae | Tonguefish | *Cynoglossus robustus* | China | GI tract | 9 | 0.67 | | Su et al. [277] |
|  |  |  |  | *Symphurus tessellatus* | Brazil | GI tract | 26 | 0.04 | | Vendel et al. [283] |
|  |  | Diodontidae | Porcupinefish | *Chilomycterus spinosus spinosus* | Brazil | GI tract | 1 | 0 | | Vendel et al. [283] |
|  |  | Engraulidae | Anchovy | *Setipinna taty* | China | GI tract | 20 | 0.35 | | Sun et al. [278] |
|  |  | Gempylidae | Snoek | *Thyrsites atun* | South Pacific | GI tract | 28 | 0.61 | | Markic et al. [249] |
|  |  | Haemulidae | Grunt | *Anisotremus surinamensis* | Brazil | GI tract | 1 | 0 | | Pegado et al. [263] |
|  |  |  |  |  | Brazil | GI tract | 1 | 0 | | Vendel et al. [283] |
|  |  |  |  | *Anisotremus virginicus* | Brazil | GI tract | 1 | 0 | | Pegado et al. [263] |
|  |  |  |  | *Conodon nobilis* | Brazil | GI tract | 8 | 0 | | Pegado et al. [263] |
|  |  |  |  | *Haemulopsis corvinaeformis* | Brazil | Stomach | 44 | 1.25 | | Silva et al. [275] |
|  |  |  |  | *Orthopristis ruber* | Brazil | GI tract | 2 | 0 | | Pegado et al. [263] |
|  |  |  |  | *Pomadasys corvinaeformis* | Brazil | GI tract | 4 | 0 | | Vendel et al. [283] |
|  |  | Holocentridae | Squirrelfish | *Neoniphon sammara* | Red Sea | GI tract | 5 | 0.20 | | Baalkhuyur et al. [188] |
|  |  |  |  | *Sargocentron spiniferum* | Red Sea | GI tract | 5 | 0 | | Baalkhuyur et al. [188] |
|  |  | Mullidae | Goatfish | *Upeneus moluccensis* | Turkey | GI tract | 18 | 0.78 | | Guven et al. [226] |
|  |  | Oxudercidae | Goby | *Synechogobius ommaturus* | China | GI tract | 17 | 3.70 | | Su et al. [277] |
|  |  | Paralichthyidae | Flounder | *Citharichthys spilopterus* | Brazil | GI tract | 12 | 0 | | Vendel et al. [283] |
|  |  | Platycephalidae | Flathead | *Platycephalus indicus* | Persian Gulf | WO | 12 | 21.80 | | Abbasi et al. [180] |
|  |  | Priacanthidae | Glasseye | *Heteropriacanthus cruentatus* | South Pacific | GI tract | 10 | 0.30 | | Markic et al. [249] |
|  |  | Sciaenidae | Croaker | *Collichthys lucidus* | China | GI tract | 18 | 6.20 | | Jabeen et al. [231] |
|  |  |  |  |  | China | GI tract | 26 | 1.20 | | Su et al. [277] |
|  |  | Scombridae | Mackerel | *Scomber scombrus* | North Sea | GI tract | 51 | 0.03 | | Rummel et al. [273] |
|  |  |  |  |  | Portugal | Stomach | 12 | 0.46 | | Neves et al. [259] |
|  |  |  |  |  | UK | GI tract | 31 | 0.58 | | Nelms et al. [257] |
|  |  | Sparidae | Porgy | *Diplodus annularis* | Turkey | GI tract | 48 | 1.96 | | Guven et al. [226] |
|  |  |  |  | *Pagrus auratus* | South Pacific | GI tract | 22 | 0.05 | | Markic et al. [249] |
|  |  | Tetraodontidae | Pufferfish | *Colomesus psittacus* | Brazil | GI tract | 2 | 0 | | Pegado et al. [263] |
|  |  |  |  |  | Brazil | GI tract | 5 | 0 | | Vendel et al. [283] |
|  |  | Terapontidae | Terapon | *Pelates quadrilineatus* | Turkey | GI tract | 135 | 1.48 | | Guven et al. [226] |
|  | Elasmobranchii | Triakidae | Houndshark | *Mustelus canis* | Brazil | GI tract | 2 | 0 | | Pegado et al. [263] |
|  |  |  |  | *Mustelus higmani* | Brazil | GI tract | 3 | 0 | | Pegado et al. [263] |
| **3.7** | Actinopterygii | Apogonidae | Perch | *Apogon lineatus* | China | GI tract | 20 | 0.40 | | Sun et al. [278] |
|  |  | Batrachoididae | Toadfish | *Batrachoides surinamensis* | Brazil | GI tract | 1 | 0 | | Pegado et al. [263] |
|  |  | Carangidae | Mackerel | *Trachurus trachurus* | North Sea | GI tract | 100 | 0.01 | | Foekema et al. [220] |
|  |  |  |  |  | Portugal | Stomach | 44 | 0.07 | | Neves et al. [259] |
|  |  |  |  |  | Scotland | GI tract | 5 | 0 | | Murphy et al. [254] |
|  |  |  |  |  | UK | GI tract | 56 | 1.50 | | Lusher et al. [244] |
|  |  |  | Moonfish | *Selene setapinnis* | Brazil | GI tract | 1 | 0 | | Pegado et al. [263] |
|  |  |  | Scad | *Alepes vari* | Thailand | Stomach | 3 | 1.70 | | Azad et al. [187] |
|  |  | Engraulidae | Anchovy | *Lycengraulis grossidens* | Brazil | GI tract | 146 | 0.18 | | Vendel et al. [283] |
|  |  | Gadidae | Cod | *Trisopterus minutus* | UK | GI tract | 50 | 2.00 | | Lusher et al. [244] |
|  |  |  |  |  | UK | GI tract | 1 | 0 | | McGoran et al. [251] |
|  |  |  |  | *T. minutus* larvae | UK | GI tract | 53 | 0.02 | | Steer et al. [276] |
|  |  |  | Pout | *Trisopterus luscus* | UK | GI tract | 7 | 1.00 | | McGoran et al. [251] |
|  |  | Gobiidae | Goby | *Bathygobius soporator* | Brazil | GI tract | 6 | 0 | | Vendel et al. [283] |
|  |  | Haemulidae | Grunt | *Haemulon steindachneri* | Brazil | GI tract | 5 | 0 | | Pegado et al. [263] |
|  |  | Lethrinidae | Bream | *Gnathodentex aureolineatus* | South Pacific | GI tract | 29 | 0.07 | | Markic et al. [249] |
|  |  | Polynemidae | Threadfin | *Polydactylus oligodon* | Brazil | GI tract | 1 | 3.00 | | Pegado et al. [263] |
|  |  |  |  | *Polydactylus virginicus* | Brazil | GI tract | 13 | 0 | | Pegado et al. [263] |
|  |  |  |  |  | Brazil | GI tract | 14 | 0 | | Vendel et al. [283] |
|  |  | Sciaenidae | Croaker | *Johnius borneensis* | Thailand | Stomach | 3 | 1.00 | | Azad et al. [187] |
|  |  |  |  | *Larimichthys crocea* | China | GI tract | 18 | 4.60 | | Jabeen et al. [231] |
|  |  |  |  | *Larimichthys polyactis* | China | GI tract | 30 | 0.97 | | Sun et al. [278] |
|  |  | Scomberesocidae | Saury | *Cololabis saira* | Pacific Ocean | Stomach | 54 | 3.20 | | Boerger et al. [194] |
|  |  | Sebastidae | Rockfish | *Sebastiscus marmoratus* | China | GI tract | 18 | 4.20 | | Jabeen et al. [231] |
|  |  | Serranidae | Grouper | *Epinephelus areolatus* | Red Sea | GI tract | 5 | 0.20 | | Baalkhuyur et al. [188] |
|  |  | Sparidae | Seabream | *Sparus aurata* | Turkey | GI tract | 110 | 0.87 | | Guven et al. [226] |
|  |  | Triglidae | Gurnard | *Chelidonichthys kumu* | China | GI tract | 177 | 0.45 | | Sun et al. [278] |
|  |  |  |  |  | South Pacific | GI tract | 27 | 0.07 | | Markic et al. [249] |
|  |  |  |  | *Trigla lyra* | Portugal | Stomach | 31 | 0.26 | | Neves et al. [259] |
| **3.8** | Actinopterygii | Carangidae | Mackerel | *Trachurus mediterraneus* | Turkey | GI tract | 98 | 1.77 | | Guven et al. [226] |
|  |  |  | Leatherjacket | *Oligoplites saliens* | Brazil | GI tract | 2 | 0 | | Vendel et al. [283] |
|  |  | Haemulidae | Grunt | *Haemulon plumierii* | Brazil | GI tract | 13 | 0 | | Pegado et al. [263] |
|  |  |  |  | *Pomadasys incisus* | Turkey | GI tract | 29 | 0.79 | | Guven et al. [226] |
|  |  | Hexagrammidae | Greenling | *Hexagrammos otakii* | China | GI tract | 40 | 0.38 | | Sun et al. [278] |
|  |  | Lethrinidae | Bream | *Gymnocranius grandoculis* | Red Sea | GI tract | 10 | 0.20 | | Baalkhuyur et al. [188] |
|  |  | Lethrinidae | Emperor | *Lethrinus microdon* | Red Sea | GI tract | 10 | 0.20 | | Baalkhuyur et al. [188] |
|  |  | Lutjanidae | Snapper | *Lutjanus alexandrei* | Brazil | GI tract | 1 | 0 | | Vendel et al. [283] |
|  |  |  |  | *Lutjanus synagris* | Brazil | GI tract | 2 | 0 | | Vendel et al. [283] |
|  |  |  |  |  | Brazil | GI tract | 2 | 0.50 | | Pegado et al. [263] |
|  |  |  |  | *Pristipomoides multidens* | Red Sea | GI tract | 10 | 0.20 | | Baalkhuyur et al. [188] |
|  |  | Nemipteridae | Bream | *Nemipterus randalli* | Turkey | GI tract | 135 | 1.31 | | Guven et al. [226] |
|  |  | Sciaenidae | Weakfish | *Cynoscion jamaicensis* | Brazil | GI tract | 3 | 0 | | Pegado et al. [263] |
|  |  |  | Croaker | *Sciaena umbra* | Turkey | GI tract | 1 | 3.00 | | Guven et al. [226] |
|  |  | Serranidae | Grouper | *Epinephelus merra* | French Polynesia | GI tract | 33 | 0.39 | | Garnier et al. [221] |
|  |  |  | Soapfish | *Rypticus randalli* | Brazil | GI tract | 1 | 0 | | Vendel et al. [283] |
|  |  | Sparidae | Seabream | *Pagellus acarne* | Portugal | Stomach | 1 | 1.00 | | Neves et al. [259] |
|  |  | Sparidae | Seabream | *Pagellus acarne* | Turkey | GI tract | 52 | 1.63 | | Guven et al. [226] |
|  |  | Triglidae | Gurnard | *Chelidonichthys cuculus* | UK | GI tract | 55 | 2.00 | | Lusher et al. [244] |
|  |  |  |  |  | UK | GI tract | 6 | 0 | | McGoran et al. [251] |
|  |  |  |  | *Prionotus punctatus* | Brazil | GI tract | 1 | 0 | | Vendel et al. [283] |
|  | Elasmobranchii | Rajidae | Ray | *Raja asterias* | Portugal | Stomach | 7 | 0.57 | | Neves et al. [259] |
|  |  |  |  | *Raja clavata* | UK | GI tract | 7 | 1.00 | | McGoran et al. [251] |
|  |  | Scyliorhinidae | Dogfish | *Scyliorhinus canicula* | Portugal | Stomach | 20 | 0.40 | | Neves et al. [259] |
|  |  |  |  |  | Spain | GI tract | 72 | 1.20 | | Bellas et al. [189] |
|  |  |  |  |  | UK | GI tract | 8 | 1.50 | | McGoran et al. [251] |
| **3.9** | Actinopterygii | Butidae | Goby | *Oxyeleotris marmorata* | China | GI tract | 18 | 4.20 | | Jabeen et al. [231] |
|  |  | Carangidae | Scad | *Megalaspis cordyla* | Thailand | Stomach | 29 | 1.60 | | Azad et al. [187] |
|  |  | Exocoetidae | Flying fish | *Cheilopogon pitcairnensis* | South Pacific | GI tract | 21 | 0.10 | | Markic et al. [249] |
|  |  | Lethrinidae | Emporer | *Lethrinus obsoletus* | South Pacific | GI tract | 30 | 0.17 | | Markic et al. [249] |
|  |  | Lutjanidae | Snapper | *Lipocheilus carnolabrum* | Red Sea | GI tract | 7 | 0.29 | | Baalkhuyur et al. [188] |
|  |  |  |  | *Lutjanus analis* | Brazil | GI tract | 3 | 0.33 | | Pegado et al. [263] |
|  |  |  |  | *Lutjanus kasmira* | Red Sea | GI tract | 12 | 0.17 | | Baalkhuyur et al. [188] |
|  |  | Sciaenidae | Drum | *Ctenosciaena gracilicirrhus* | Brazil | GI tract | 11 | 0 | | Pegado et al. [263] |
|  |  |  | Weakfish | *Macrodon ancylodon* | Brazil | GI tract | 13 | 0.15 | | Pegado et al. [263] |
|  |  | Scombridae | Mackerel | *Scomber colias* | Canary Islands | GI tract | 120 | 2.17 | | Herrera et al. [230] |
|  |  | Scorpaeniformes | Sculpin | *Myoxocephalus scorpius* | UK | GI tract | 5 | 0 | | McGoran et al. [251] |
|  |  | Sparidae | Porgy | *Pagrus pagrus* | Turkey | GI tract | 9 | 1.44 | | Guven et al. [226] |
|  |  | Terapontidae | Terapon | *Terapon jarbua* | China | GI tract | 18 | 2.00 | | Jabeen et al. [231] |
|  | Elasmobranchii | Sphyrnidae | Bonnethead Shark | *Sphyrna tiburo* | Brazil | GI tract | 2 | 9.00 | | Pegado et al. [263] |
| **4** | Actinopterygii | Achiridae | Sole | *Achirus achirus* | Brazil | GI tract | 3 | 0 | | Vendel et al. [283] |
|  |  | Ariidae | Sea Catfish | *Bagre bagre* | Brazil | GI tract | 7 | 9.14 | | Pegado et al. [263] |
|  |  |  |  | *Notarius grandicassis* | Brazil | GI tract | 4 | 0 | | Pegado et al. [263] |
|  |  | Carangidae | Trevally | *Caranx papuensis* | South Pacific | GI tract | 32 | 1.03 | | Markic et al. [249] |
|  |  | Centrolophidae | Rudderfish | *Psenopsis anomala* | China | GI tract | 18 | 1.10 | | Jabeen et al. [231] |
|  |  |  |  |  | China | GI tract | 10 | 0.20 | | Sun et al. [278] |
|  |  | Clupeidae | Shad | *Alosa fallax* | Portugal | Stomach | 1 | 1.00 | | Neves et al. [259] |
|  |  | Exocoetidae | Flying fish | *Cheilopogon simus* | French Polynesia | GI tract | 34 | 0.24 | | Garnier et al. [221] |
|  |  | Gadidae | Haddock | *Melanogrammus aeglefinus* | North Sea | GI tract | 97 | 0.08 | | Foekema et al. [220] |
|  |  |  |  |  | UK | GI tract | 6 | 5.83 | | McGoran et al. [251] |
|  |  | Haemulidae | Grunt | *Plectorhinchus gaterinus* | Red Sea | GI tract | 6 | 0.33 | | Baalkhuyur et al. [188] |
|  |  | Lethrinidae | Emporer | *Lethrinus amboinensis* | South Pacific | GI tract | 26 | 0.38 | | Markic et al. [249] |
|  |  | Ophichthidae | Eel | *Ophichthus cylindroideus* | Brazil | GI tract | 1 | 0 | | Pegado et al. [263] |
|  |  |  |  |  | Brazil | GI tract | 1 | 0 | | Vendel et al. [283] |
|  |  | Pleuronectidae | Halibut | *Hippoglossus hippoglossus* | Scotland | GI tract | 14 | 0 | | Murphy et al. [254] |
|  |  | Priacanthidae | Bigeye | *Priacanthus arenatus* | Brazil | GI tract | 122 | 1.72 | | Cardozo et al. [200] |
|  |  | Rachycentridae | Cobia | *Rachycentron canadum* | Brazil | GI tract | 1 | 0 | | Pegado et al. [263] |
|  |  | Sciaenidae | Croaker | *Micropogonias undulatus* | Gulf of Mexico | Stomach | 383 | 0.87 | | Peters et al. [266] |
|  |  |  | Weakfish | *Cynoscion microlepidotus* | Brazil | GI tract | 16 | 0.25 | | Pegado et al. [263] |
|  |  |  |  | *Cynoscion virescens* | Brazil | GI tract | 7 | 0.43 | | Pegado et al. [263] |
|  |  | Scophthalmidae | Flatfish | *Phrynorhombus norvegicus* | UK | GI tract | 1 | 0 | | McGoran et al. [251] |
|  |  | Serranidae | Grouper | *Epinephelus chlorostigma* | Red Sea | GI tract | 3 | 0.33 | | Baalkhuyur et al. [188] |
|  |  |  |  | *Epinephelus epistictus* | Red Sea | GI tract | 5 | 0.20 | | Baalkhuyur et al. [188] |
|  |  |  |  | *Epinephelus radiatus* | Red Sea | GI tract | 7 | 0.14 | | Baalkhuyur et al. [188] |
|  |  | Stomiidae | Dragonfish | *Stomias boa boa* | Atlantic Ocean | GI tract | 5 | 0.80 | | Lusher et al. [246] |
|  |  |  | Snaggletooth | *Astronesthes indopacificus* | Pacific Ocean | Stomach | 7 | 1.00 | | Boerger et al. [194] |
|  |  | Triglidae | Gurnard | *Chelidonichthys lucerna* | Mediterranean | GI tract | 3 | 1.00 | | Avio et al. [184] |
|  |  |  |  |  | Turkey | GI tract | 24 | 0.75 | | Guven et al. [226] |
| **4.1** | Actinopterygii | Aploactinidae | Velvetfish | *Erisphex pottii* | China | GI tract | 120 | 0.32 | | Sun et al. [278] |
|  |  | Bramidae | Pomfret | *Brama brama* | Portugal | Stomach | 3 | 0.67 | | Neves et al. [259] |
|  |  | Carangidae | Jack | *Caranx crysos* | Turkey | GI tract | 1 | 5.00 | | Guven et al. [226] |
|  |  |  |  |  | Brazil | GI tract | 3 | 0 | | Pegado et al. [263] |
|  |  | Carangidae | Queenfish | *Scomberoides tol* | Thailand | Stomach | 3 | 2.20 | | Azad et al. [187] |
|  |  | Gadidae | Cod | *Gadus morhua* | North Sea | GI tract | 80 | 0.14 | | Foekema et al. [220] |
|  |  |  |  |  | North Sea | GI tract | 81 | 0.01 | | Rummel et al. [273] |
|  |  |  |  |  | UK | GI tract | 3 | 0 | | McGoran et al. [251] |
|  |  |  | Whiting | *Micromesistius poutassou* | Scotland | GI tract | 20 | 0 | | Murphy et al. [254] |
|  |  |  |  |  | UK | GI tract | 27 | 2.00 | | Lusher et al. [244] |
|  |  | Lophiidae | Goosefish | *Lophius litulon* | China | GI tract | 20 | 0.25 | | Sun et al. [278] |
|  |  | Lutjanidae | Snapper | *Lutjanus gibbus* | South Pacific | GI tract | 29 | 0.41 | | Markic et al. [249] |
|  |  | Muraenidae | Eel | *Gymnothorax ocellatus* | Brazil | GI tract | 1 | 0 | | Pegado et al. [263] |
|  |  | Pleuronectidae | Flounder | *Hippoglossoides platessoides* | UK | GI tract | 104 | 2.67 | | McGoran et al. [251] |
|  |  | Sciaenidae | Weakfish | *Cynoscion acoupa* | Brazil | GI tract | 552 | 1.91 | | Ferreira et al. [219] |
|  |  | Serranidae | Grouper | *Epinephelus itajara* | Brazil | GI tract | 2 | 0 | | Pegado et al. [263] |
|  |  | Sparidae | Porgy | *Dentex gibbosus* | Turkey | GI tract | 14 | 0.29 | | Guven et al. [226] |
|  |  | Trichiuridae | Hairtail | *Eupleurogrammus muticus* | China | GI tract | 15 | 0.33 | | Sun et al. [278] |
| **4.2** | Actinopterygii | Carangidae | Jack | *Caranx latus* | Brazil | GI tract | 57 | 0 | | Vendel et al. [283] |
|  |  |  |  | *Seriola lalandi* | South Pacific | GI tract | 15 | 0.27 | | Markic et al. [249] |
|  |  | Centrolophidae | Warehou | *Schedophilus velaini* | South Pacific | GI tract | 14 | 1.43 | | Markic et al. [249] |
|  |  | Centropomidae | Snook | *Centropomus ensiferus* | Brazil | GI tract | 1 | 0 | | Vendel et al. [283] |
|  |  |  |  | *Centropomus undecimalis* | Brazil | GI tract | 2 | 0 | | Vendel et al. [283] |
|  |  | Gadidae | Cod | *Gadus macrocephalus* | China | GI tract | 40 | 0.43 | | Sun et al. [278] |
|  |  | Lutjanidae | Snapper | *Pristipomoides typus* | Red Sea | GI tract | 5 | 0 | | Baalkhuyur et al. [188] |
|  |  | Sciaenidae | Weakfish | *Cynoscion arenarius* | Gulf of Mexico | Stomach | 139 | 0.79 | | Peters et al. [266] |
|  |  | Synodontidae | Lizardfish | *Harpadon nehereus* | China | GI tract | 18 | 3.80 | | Jabeen et al. [231] |
|  |  |  |  |  | China | GI tract | 18 | 2.50 | | Su et al. [277] |
|  | Elasmobranchii | Pentanchidae | CatShark | *Galeus melastomus* | Mediterranean | Stomach | 21 | 0.34 | | Alomar and Deudero [181] |
| **4.24** | Reptilia | Cheloniidae | Leatherback Sea Turtle | *Dermochelys coriacea* | Atlantic | GI tract | 2 | 1.5 | | Duncan et al. [294] |
| **4.25** | Actinopterygii | Serranidae | Trout | *Plectropomus spp.** | Australia | GI tract | 20 | 5.80 | | Kroon et al. [238] |
| **4.27** | Mammalia | Balaenopteridae | Humpback Whale | *Megaptera novaeangliae* | Netherlands | GI tract | 1 | 16.00 | | Besseling et al. [191] |
| **4.3** | Actinopterygii | Ariidae | SeaCatfish | *Cathorops agassizii* | Brazil | GI tract | 1 | 0 | | Vendel et al. [283] |
|  |  | Carangidae | Leatherjacket | *Oligoplites palometa* | Brazil | GI tract | 8 | 0 | | Vendel et al. [283] |
|  |  |  |  | *Oligoplites saurus* | Brazil | GI tract | 22 | 0.05 | | Vendel et al. [283] |
|  |  |  | Moonfish | *Selene vomer* | Brazil | GI tract | 2 | 1.00 | | Pegado et al. [263] |
|  |  |  | Moonfish | *Selene vomer* | Brazil | GI tract | 2 | 0 | | Vendel et al. [283] |
|  |  | Gadidae | Pollack | *Pollachius pollachius* | Scotland | GI tract | 5 | 0 | | Murphy et al. [254] |
|  |  | Liparidae | Snailfish | *Liparis tanakae* | China | GI tract | 245 | 0.36 | | Sun et al. [278] |
|  |  | Phycidae | Forkbeard | *Phycis phycis* | Italy | GI tract | 7 | 3.38 | | Avio et al. [295] |
|  |  | Sciaenidae | Croaker | *Argyrosomus regius* | Portugal | Stomach | 5 | 0.80 | | Neves et al. [259] |
|  |  |  |  |  | Turkey | GI tract | 51 | 1.84 | | Guven et al. [226] |
|  |  | Scombridae | Mackerel | *Scomberomorus guttatus* | Thailand | Stomach | 5 | 0.60 | | Azad et al. [187] |
|  |  |  | Tuna | *Thunnus alalunga* | Mediterranean | Stomach | 31 | 0.13 | | Romeo et al. [272] |
|  |  | Scophthalmidae | Megrim | *Lepidorhombus whiffiagonis* | Scotland | GI tract | 10 | 0.10 | | Murphy et al. [254] |
| **4.36** | Mammalia | Delphinidae | Risso's dolphin | *Grampus griseus* | UK | GI tract | 1 | 9.00 | | Nelms et al. [296] |
| **4.37** | Mammalia | Delphinidae | White-beaked dolphin | *Lagenorhynchus albirostris* | UK | GI tract | 1 | 3.00 | | Nelms et al. [296] |
| **4.4** | Actinopterygii | Coryphaenidae | Dolphinfish | *Coryphaena hippurus* | South Pacific | GI tract | 10 | 0.40 | | Markic et al. [249] |
|  |  | Gadidae | Whiting | *Merlangius merlangus* | North Sea | GI tract | 105 | 0.10 | | Foekema et al. [220] |
|  |  |  |  |  | UK | GI tract | 50 | 1.75 | | Lusher et al. [244] |
|  |  |  |  |  | UK | GI tract | 29 | 4.67 | | McGoran et al. [251] |
|  |  | Lotidae | Ling | *Molva molva* | Scotland | GI tract | 5 | 0 | | Murphy et al. [254] |
|  |  | Lutjanidae | Snapper | *Lutjanus jocu* | Brazil | GI tract | 4 | 0 | | Vendel et al. [283] |
|  |  | Merlucciidae | Hake | *Merluccius merluccius* | Mediterranean | GI tract | 3 | 1.33 | | Avio et al. [184] |
|  |  |  |  |  | Mediterranean | GI tract | 97 | 0.38 | | Giani et al. [222] |
|  |  |  |  |  | Mediterranean | Stomach | 67 | 0.46 | | Mancuso et al. [248] |
|  |  |  |  |  | Portugal | Stomach | 12 | 0.35 | | Neves et al. [259] |
|  |  |  |  |  | Spain | GI tract | 12 | 1.00 | | Bellas et al. [189] |
|  |  | Muraenesocidae | Pike Cogner | *Muraenesox cinereus* | China | GI tract | 18 | 2.40 | | Jabeen et al. [231] |
|  |  | Salmonidae | Salmon | *Oncorhynchus tshawytscha* | Canada | GI tract | 74 | 1.15 | | Collicutt et al. [210] |
|  |  | Scombridae | Tuna | *Katsuwonus pelamis* | South Pacific | GI tract | 26 | 0.35 | | Markic et al. [249] |
|  |  |  |  | *Thunnus albacares* | South Pacific | GI tract | 68 | 0.68 | | Markic et al. [249] |
|  |  | Sparidae | Pinfish | *Lagodon rhomboides* | Gulf of Mexico | Stomach | 449 | 0.96 | | Peters et al. [266] |
|  |  | Sphyrnidae | Barracudina | *Sphyraena forsteri* | South Pacific | GI tract | 12 | 0.25 | | Markic et al. [249] |
|  |  | Synodontidae | Lizardfish | *Saurida tumbil* | Persian Gulf | WO | 4 | 13.50 | | Abbasi et al. [180] |
|  |  | Trichiuridae | Hairtail | *Trichiurus lepturus* | Brazil | GI tract | 5 | 0.40 | | Pegado et al. [263] |
|  |  | Uranoscopidae | Stargazer | *Uranoscopus scaber* | Italy | GI tract | 7 | 3.20 | | Avio et al. [185] |
|  | Chondrichthyes | Squalidae | Dogfish | *Squalus acanthias* | Mediterranean | GI tract | 9 | 1.25 | | Avio et al. [184] |
|  | Elasmobranchii | Carcharhinidae | Shark | *Prionace glauca* | Mediterranean | Stomach | 139 | 0.20 | | Bernardini et al. [190] |
|  | Mammalia | Phocoenidae | Finless porpoise | *Neophocaena phocaenoides* | China | GI tract | 7 | 19.14 | | Xiong et al. [290] |
| **4.44** | Mammalia | Kogiidae | Pygmy sperm whale | *Kogia breviceps* | UK | GI tract | 1 | 4.00 | | Nelms et al. [296] |
| **4.47** | Mammalia | Delphinidae | Striped dolphin | *Stenella coeruleoalba* | UK | GI tract | 1 | 7.00 | | Nelms et al. [296] |
| **4.48** | Mammalia | Phocoenidae | Harbour porpoise | *Phocoena phocoena* | UK | GI tract | 21 | 5.24 | | Nelms et al. [296] |
| **4.49** | Mammalia | Phocidae | Grey seal | *Halichoerus grypus* | UK | Scat | 31 | 0.84 | | Nelms et al. [257] |
|  |  |  |  |  | UK | GI tract | 3 | 6.00 | | Nelms et al. [296] |
| **4.5** | Actinopterygii | Belonidae | Needlefish | *Strongylura timucu* | Brazil | GI tract | 2 | 0 | | Vendel et al. [283] |
|  |  | Carangidae | Queenfish | *Scomberoides tala* | Thailand | Stomach | 3 | 0.70 | | Azad et al. [187] |
|  |  | Clupeidae | Herring | *Opisthonema oglinum* | Brazil | GI tract | 56 | 0.23 | | Vendel et al. [283] |
|  |  | Ephippidae | Spadefish | *Chaetodipterus faber* | Brazil | GI tract | 6 | 0.17 | | Vendel et al. [283] |
|  |  |  |  |  | Brazil | GI tract | 5 | 0 | | Pegado et al. [263] |
|  |  |  |  |  | Gulf of Mexico | Stomach | 103 | 1.38 | | Peters et al. [266] |
|  |  | Lophiidae | Anglerfish | *Lophius piscatorius* | Portugal | Stomach | 2 | 0.50 | | Neves et al. [259] |
|  |  | Ophichthidae | Eel | *Ophichthus ophis* | Brazil | GI tract | 1 | 0 | | Pegado et al. [263] |
|  |  | Pomatomidae | Bluefish | *Pomatomus saltatrix* | Brazil | GI tract | 2 | 0 | | Pegado et al. [263] |
|  |  | Scombridae | Mackerel | *Scomberomorus commerson* | Thailand | Stomach | 4 | 4.30 | | Azad et al. [187] |
|  |  |  | Tuna | *Thunnus thynnus* | Mediterranean | Stomach | 36 | 0.44 | | Romeo et al. [272] |
|  |  | Serranidae | Hind | *Cephalopholis argus* | Red Sea | GI tract | 4 | 0 | | Baalkhuyur et al. [188] |
|  |  | Stromateidae | Butterfish | *Peprilus paru* | Brazil | GI tract | 2 | 0 | | Pegado et al. [263] |
|  |  | Synodontidae | Lizardfish | *Saurida undosquamis* | Turkey | GI tract | 99 | 1.22 | | Guven et al. [226] |
|  |  | Trichiuridae | Scabbardfish | *Aphanopus carbo* | Scotland | GI tract | 5 | 0 | | Murphy et al. [254] |
|  |  | Xiphiidae | Swordfish | *Xiphias gladius* | Mediterranean | Stomach | 56 | 0.16 | | Romeo et al. [272] |
|  |  | Zeidae | John Dory | *Zeus faber* | Portugal | Stomach | 1 | 1.00 | | Neves et al. [259] |
|  |  |  |  |  | UK | GI tract | 42 | 2.70 | | Lusher et al. [244] |
|  | Mammalia | Delphinidae | Atlantic white-sided dolphin | *Lagenorhynchus acutus* | UK | GI tract | 1 | 8.00 | | Nelms et al. [296] |
|  |  |  | Bottlenose dolphin | *Tursiops truncatus* | UK | GI tract | 1 | 6.00 | | Nelms et al. [296] |
|  |  |  | Common Dolphin | *Delphinus delphis* | Spain | Stomach | 35 | 11.74 | | Hernandez-Gonzalez et al. [229] |
|  |  |  |  |  | UK | GI tract | 16 | 5.69 | | Nelms et al. [296] |
|  |  |  | Humpback Dolphin | *Sousa chinensis* | China | GI tract | 3 | 25.67 | | Zhu et al. [293] |
|  |  | Otariidae | Fur seal | *Arctocephalus australis* | Chile | Scat | 51 | 24.86 | | Perez-Venegas et al. [265] |
|  |  |  |  | *Callorhinus ursinus* | USA | Scat | 44 | 13.27 | | Donohue et al. [217] |
|  |  | Phocidae | Harbour seal | *Phoca vitulina* | UK | GI tract | 4 | 4.25 | | Nelms et al. [296] |
|  |  | Ziphiidae | Beaked whale | *Mesoplodon mirus* | Ireland | GI tract | 3 | 29.33 | | Lusher et al. [245] |

Data has been grouped into trophic levels based on the well-established FishBase [297] and SeaLifeBase [298] databases and organised by class and family, with sample sizes (n) given.

WO: Whole organism

^a^Wild caught bivalves only.

* *Plectropomus spp.* refers to *P.* *leopardus* (trophic level = 4.4) and *P. maculatus* (trophic level = 4.11) examined by Kroon et al. [238]. Trophic level given is an average of the two species (trophic level = 4.25).

### Calculations for standardisation of MP contamination data

| **Trophic Level** | **Class** | **Family** | **Common Name** | ***Species*** | **Average Reported (MPs g^-1^)** | **Soft Tissue Weight**  **(g individual^-1^)** | **Calculated Abundance**  **(MPs individual^-1^)** | **Reference** |
| --- | --- | --- | --- | --- | --- | --- | --- | --- |
| **2** | Bivalvia | Arcidae | Clam | *Scapharca subcrenata* | 10.50 | 4.43 | 46.52 | Li et al. [240] |
|  |  |  |  | *Tegillarca granosa* | 4.00 | 1.29 | 5.16 | Li et al. [240] |
|  |  | Mytilidae | Mussel | *Mytilus edulis* | 2.20 | 1.79 | 3.94 | Li et al. [241] |
|  |  |  |  |  | 0.13 | 5.74 | 0.73 | Vandermeersch et al. [282] |
|  |  | Ostreidae | Oyster | *Alectryonella plicatula* | 5.50 | 1.84 | 10.12 | Li et al. [240] |
|  |  |  |  | *Crassostrea gigas* | 0.08 | 2.25 | 0.18 | Bonello et al. [195] |
|  |  | Pectinidae | Scallop | *Mizuhopecten yessoensis* | 2.30 | 24.80 | 57.04 | Li et al. [240] |
|  |  | Pharidae | Clam | *Sinonovacula constricta* | 2.00 | 7.53 | 15.06 | Li et al. [240] |
|  |  | Veneridae | Clam | *Cyclina sinensis* | 4.00 | 1.19 | 4.76 | Li et al. [240] |
|  |  |  |  | *Meretrix lusoria* | 4.20 | 2.33 | 9.79 | Li et al. [240] |
|  |  |  |  | *Ruditapes philippinarum* | 0.90 | 10.00 | 9.00 | Davidson and Dudas [212] |
|  |  |  |  |  | 2.50 | 2.21 | 5.53 | Li et al. [240] |
| **2.69** | Bivalvia | Mytilidae | Mussel | *Mytilus galloprovincialis* | 2.50 | 1.79 | 4.48 | Li et al. [240] |
|  |  |  |  |  | 0.15 | 5.15 | 0.76 | Vandermeersch et al. [282] |

Data presented here is included in S3 Table (above).

S4 Table. Bioaccumulation of chemical additives associated with microplastic (MP) uptake for marine organisms collected *in situ*. Chemical additive concentrations per individual (**ng g^-1^**; i.e. body burden) were based on reported and quantified concentrations of additives in the tissues of each species, with concentrations representative of additives per number of total organisms in the sample size for a particular species, rather than taken from only the number of organisms that exhibited additive contamination.

| **Trophic Level** | **Class** | **Family** | **Common Name** | ***Species Name*** | **Location** | **Area Investigated** | **n** | **MP**  **Individual^-1^** | **Chemical Additives** | **Concentration Reported**  **(ng g^-1^)** | **Reference** |
| --- | --- | --- | --- | --- | --- | --- | --- | --- | --- | --- | --- |
| **2** | Bivalvia | Cardiidae | Clam | *Cerastoderma edule* | France | Soft tissue | 50 | 5.72 | ∑PAH | 0.06 | Hermabessiere et al. [228] |
|  |  |  |  |  |  |  |  |  | ∑PBDE | 0.70 | Hermabessiere et al. [228] |
|  |  |  |  |  |  |  |  |  | ∑PCB | 0 | Hermabessiere et al. [228] |
|  |  |  |  |  |  |  |  |  | ∑Phthalates | 52.36 | Hermabessiere et al. [228] |
|  |  | Mytilidae | Mussel | *Mytilus edulis* | France | Soft tissue | 50 | 5.88 | ∑PAH | 5.48 | Hermabessiere et al. [228] |
|  |  |  |  |  |  |  |  |  | ∑PBDE | 0.07 | Hermabessiere et al. [228] |
|  |  |  |  |  |  |  |  |  | ∑PCB | 1 | Hermabessiere et al. [228] |
|  |  |  |  |  |  |  |  |  | ∑Phthalates | 26.36 | Hermabessiere et al. [228] |
| **2.2** | Ascidiacea | Pyuridae | Ascidian | *Microcosmus exasperatus* | Israel | Soft tissue | 15 | 1.78 | DBP | 1,643 – 2,224 | Vered et al. [284] |
|  |  |  |  |  |  |  |  |  | DEHP | 4,851 – 4,988 | Vered et al. [284] |
|  |  |  |  |  |  |  |  |  | DnOP | 0 | Vered et al. [284] |

Sampling locations and sizes (n) have been included. Data has been grouped into trophic levels based on the well-established SeaLifeBase database [298]. See above for abbreviations related to chemical additives used.

S5 Table. Experimental details of microplastic (MP) laboratory exposures conducted with marine organisms. Contamination and retention, following specific exposures to MPs under controlled laboratory conditions, are presented for each species, and organised by trophic levels.

|  |  |  |  | Exposure Details | | | | | | | |  | |  | | |  |  | | |  | |  | |
| --- | --- | --- | --- | --- | --- | --- | --- | --- | --- | --- | --- | --- | --- | --- | --- | --- | --- | --- | --- | --- | --- | --- | --- | --- |
|  | Class | *Species* | n | Polymer | Size (µm) | Colour | Shape | Time | Dose | Conc.  (MPs ml^-1^) | Depuration  Period | | Area | | Ingestion Sampling Frequency | Egestion Sampling Frequency | | | MPs Ind^-1^ | Retention | | Reference | |  |
| 2 | Bivalvia | *Mytilus edulis* | 16 | PP | 23 x 3000 | NM | Fi | 1 h | Once | 0.1 ^1^ | - | | ST | | - | - | | | 0.5 | - | | Porter et al. [269] | |  |
|  |  |  | 16 | PP | 23 x 3000 | NM | Fi | 1 h | Once | 0.1 ^2^ | - | | ST | | - | - | | | 6.5 | - | | Porter et al. [269] | |  |
|  |  |  | 9 | PS | 7 - 30 | NM | S | 1 h | Once | 50 ^3^ | - | | ST | | - | - | | | 340 | - | | Porter et al. [269] | |  |
|  |  |  | 9 | PS | 7 - 30 | NM | S | 1 h | Once | 50 ^2^ | - | | ST | | - | - | | | 105,000 | - | | Porter et al. [269] | |  |
|  |  |  | 9 | PE | 9 - 11 | NM | S | 1 h | Once | 50 ^2^ | - | | ST | | - | - | | | 130,000 | - | | Porter et al. [269] | |  |
|  |  |  | 9 | PS | 7 - 30 | NM | S | 1 h | Once | 50 ^1^ | - | | ST | | - | - | | | 150,000 | - | | Porter et al. [269] | |  |
|  |  |  | 9 | PE | 9 - 11 | NM | S | 1 h | Once | 50 ^1^ | - | | ST | | - | - | | | 200,000 | - | | Porter et al. [269] | |  |
|  |  |  | 5 | Mix | 100 - 3500 | Black | Fi | 5 d | Daily | 100 | - | | ST | | Daily | - | | | 5.25 | - | | Qu et al. [271] | |  |
|  |  |  | 5 | Mix | 100 | Black | Fi | 5 d | Daily | 1,000 | - | | ST | | Daily | - | | | 11.2 | - | | Qu et al. [271] | |  |
|  |  |  | 5 | PVC | 20 - 500 | Pink | Fr | 5 d | Daily | 100 | - | | ST | | Daily | - | | | 1.5 | - | | Qu et al. [271] | |  |
|  |  |  | 5 | PVC | 20 - 500 | Pink | Fr | 5 d | Daily | 1,000 | - | | ST | | Daily | - | | | 3 | - | | Qu et al. [271] | |  |
|  |  |  | 5 | PS | 10 | Green | S | 5 d | Daily | 100 | - | | ST | | Daily | - | | | 2.8 | - | | Qu et al. [271] | |  |
|  |  |  | 5 | PS | 10 | Green | S | 5 d | Daily | 1,000 | - | | ST | | Daily | - | | | 12 | - | | Qu et al. [271] | |  |
|  | Branchipoda | *Evadne nordmanni* | 7 - 10 | PS | 10 | Flu | S | 3 h | Once | 2,000 | - | | GI | | - | - | | | 0 | - | | Setala et al. [299] | |  |
|  | Echinoidea | *Tripneustes gratilla* | 25 | PE | 10 - 45 | Green (Flu) | S | 5 d | Once | 300 | 420 h | | STO | | Daily | 30 min intervals | | | 1.8 | < 2 d | | Kaposi et al. [234] | |  |
|  | Eurotatoria | *Brachionus koreanus* | 50 | PS | 0.05 - 6 | Flu | S | 24 h | Once | 0.00001 ^a^ | - | | GI | | - | - | | | 0 | - | | Jeong et al. [232] | |  |
|  | Hexanauplia | *Acartia longiremis* | 9 | PS | 30 | Flu | Fr | 24 h | Once | 0.028 | - | | GI | | - | - | | | 0 | - | | Vroom et al. [285] | |  |
|  |  | *Calanus helgolandicus* | 50 | PS | 20 | Yellow (Flu) | S | 24 h | Once | 75 | - | | Tank | | - | - | | | 3,278 | - | | Cole et al. [207] | |  |
|  |  |  | 50 | PA | 10 x 30 | NM | Fi | 6 h | Once | 80 | - | | Tank | | - | - | | | 104,100 | - | | Procter et al. [270] | |  |
|  | Malacostraca | *Allorchestes compressa* | 15 | PE | 11 - 700 | NM | Fr | 72 h | Once | 0.1 ^a^ | 36 h | | WO | | - | 0, 12, 24, 36 h | | | 18.8 | < 0.5 MPs ind^-1^ at 36 h | | Chua et al. [206] | |  |
| 2.2 | Bivalvia | *Atactodea striata* | NM | PS | 63 - 250 | NM | S | 10 d | Daily | 1 | 7 d | | Faeces | | - | - | | | 3.5 | NM | | Xu et al. [291] | |  |
|  |  | *Ennucula tenuis* | 6 | PE | 125 - 500 | NM | Fr | 4 w | Once | 1 – 25 ^c^ | - | | ST | | - | - | | | 0.83 | - | | Bour et al. [196] | |  |
|  |  | *Limecola balthica* | 54 | PS | 10 | Flu | S | 24 h | Once | 5 - 250 | - | | GI | | - | - | | | 8.1 | - | | Setala et al. [274] | |  |
|  |  | *Mytilus trossulus* | 54 | PS | 10 | Flu | S | 24 h | Once | 5 - 250 | - | | GI | | - | - | | | 27.3 | - | | Setala et al. [274] | |  |
| 2.5 | Malacostraca | *Idotea emarginata* | 24 | PS | < 100 | Blue (Flu) | Fr | 72 h | Once | 0.02 – 0.35 ^b^ | - | | GI | | - | - | | | 41.67 | - | | Hamer et al. [227] | |  |
|  |  |  | 24 | PS | 10 | Green (Flu) | S | 72 h | Once | 0.012 – 0.12 ^b^ | - | | GI | | - | - | | | 20.8 | - | | Hamer et al. [227] | |  |
| 2.69 | Bivalvia | *Mytilus galloprovincialis* | 150 | PS | 3 | Flu | S | 24 h | Once | 10,000 | 8 d | | GI | | - | 12 h intervals | | | 14.2 | 8 h to 8 d | | Capolupo et al. [199] | |  |
| 2.7 | Actinopterygii | *Acanthochromis polyacanthus* | 112 | PET | 1 - 2 | NM | S | 7 d | 2x Daily | 0.05 - 0.13 ^d^ | - | | GI | | - | - | | | 2,102* | > 6 w | | Critchell and Hoogenboom [211] | |  |
| 2.71 | Malacostraca | *Palaemonetes pugio* | 20 | PP | 93 | White | Fi | 3 h | Once | 50 | 96 h | | GI | | - | 0, 24, 48, 72, 96 h | | | 2.3 | NM | | Gray and Weinstein [225] | |  |
|  |  |  | 20 | PP | 34 | White | Fi | 3 h | Once | 50 | 96 h | | GI | | - | 0, 24, 48, 72, 96 h | | | 10 | NM | | Gray and Weinstein [225] | |  |
|  |  |  | 20 | PP | 93 | White | Fr | 3 h | Once | 50 | 96 h | | GI | | - | 0, 24, 48, 72, 96 h | | | 22 | NM | | Gray and Weinstein [225] | |  |
|  |  |  | 20 | PP | 34 | White | Fr | 3 h | Once | 50 | 96 h | | GI | | - | 0, 24, 48, 72, 96 h | | | 23 | NM | | Gray and Weinstein [225] | |  |
|  |  |  | 20 | PE | 116 | Flu | S | 3 h | Once | 50 | 96 h | | GI | | - | 0, 24, 48, 72, 96 h | | | 3 | 27.6 ± 8.57 h | | Gray and Weinstein [225] | |  |
|  |  |  | 20 | PE | 35 | Flu | S | 3 h | Once | 50 | 96 h | | GI | | - | 0, 24, 48, 72, 96 h | | | 3.5 | NM | | Gray and Weinstein [225] | |  |
|  |  |  | 20 | PE | 165 | Flu | S | 3 h | Once | 50 | 96 h | | GI | | - | 0, 24, 48, 72, 96 h | | | 5 | NM | | Gray and Weinstein [225] | |  |
|  |  |  | 20 | PE | 83 | Flu | S | 3 h | Once | 50 | 96 h | | GI | | - | 0, 24, 48, 72, 96 h | | | 7.5 | NM | | Gray and Weinstein [225] | |  |
|  |  |  | 20 | PE | 59 | Flu | S | 3 h | Once | 50 | 96 h | | GI | | - | 0, 24, 48, 72, 96 h | | | 8 | NM | | Gray and Weinstein [225] | |  |
|  |  |  | 20 | PS | 30 | Opaque | S | 3 h | Once | 50 | 96 h | | GI | | - | 0, 24, 48, 72, 96 h | | | 10 | 60.6 ± 28.5 h | | Gray and Weinstein [225] | |  |
|  |  |  | 20 | PS | 45 | Opaque | S | 3 h | Once | 50 | 96 h | | GI | | - | 0, 24, 48, 72, 96 h | | | 28.8 | 75.9 ± 13.3 h | | Gray and Weinstein [225] | |  |
| 2.8 | Bivalvia | *Abra nitida* | 2 | PE | 125 - 500 | NM | Fr | 4 w | Once | 1 – 15 ^c^ | - | | ST | | - | - | | | 1.5 | - | | Bour et al. [196] | |  |
| 2.87 | Malacostraca | *Minuca rapax* | 7 | PS | 180 - 250 | NM | Fr | 2 mths | Once | 1,000 ^c^ | - | | STO | | - | - | | | 0.14 | - | | Brennecke et al. [198] | |  |
|  |  |  | 27 | PS | 180 - 250 | NM | Fr | 2 mths | Once | 108 ^c^ | - | | STO | | - | - | | | 0.37 | - | | Brennecke et al. [198] | |  |
| 3 | Polychaeta | *Arenicola marina* | 80 | PS | 400 - 1300 | Clear | Cr | 28 d | Once | 0.001 – 0.1 ^a^ | - | | GI, Faeces | | - | - | | | 1.36 | - | | Besseling et al. [192] | |  |
| 3.5 | Actinopterygii | *Dicentrarchus labrax* | 120 | PE | 10 - 45 | Flu | S | 36 d | Daily | 1.2 ^e^ | 2 d | | WO | | 14, 20, 34 d | - | | | 1.4 | < 2 d | | Mazurais et al. [250] | |  |
|  |  |  | 120 | PE | 10 - 45 | Flu | S | 36 d | Daily | 1.2 ^e^ | 2 d | | WO | | 14, 20, 34 d | - | | | 3.3 | < 2 d | | Mazurais et al. [250] | |  |
|  |  | *Seriolella violacea* | 33 | PA | 1200 | Clear | Fr | 5 min | Once | 6.7·10^-5^ | 10 w | | Video | | - | Daily | | | 0.5 | 4.4 ± 0.9 d | | Ory et al. [261] | |  |
|  |  |  | 33 | PA | 1200 | Yellow | Fr | 5 min | Once | 6.7·10^-5^ | 10 w | | Video | | - | Daily | | | 0.6 | 4.4 ± 0.9 d | | Ory et al. [261] | |  |
|  |  |  | 33 | PA | 1200 | Blue | Fr | 5 min | Once | 6.7·10^-5^ | 10 w | | Video | | - | Daily | | | 0.8 | 4.4 ± 0.9 d | | Ory et al. [261] | |  |
|  |  |  | 33 | PA | 1200 | Black | Fr | 5 min | Once | 6.7·10^-5^ | 10 w | | Video | | - | Daily | | | 1.1 | 4.4 ± 0.9 d | | Ory et al. [261] | |  |
| 3.7 | Actinopterygii | *Sparus aurata* | 15 | PVC | 75.6 | Opaque | Fr | 45 d | Daily | 3,330 ^c^ | 30 d | | INT | | - | 0, 30 d | | | 0.07 | > 30 d | | Jovanovic et al. [233] | |  |
|  |  |  | 15 | PVC | 75.6 | Opaque | Fr | 45 d | Daily | 3,330 ^c^ | 30 d | | STO | | - | - | | | 0 | - | | Jovanovic et al. [233] | |  |
|  |  |  | 15 | HDPE | 23.4 | Opaque | Fr | 45 d | Daily | 3,330 ^c^ | 30 d | | INT | | - | 0, 30 d | | | 1.67 | > 30 d | | Jovanovic et al. [233] | |  |
|  |  |  | 15 | HDPE | 23.4 | Opaque | Fr | 45 d | Daily | 3,330 ^c^ | 30 d | | STO | | - | 0, 30 d | | | 1.8 | > 30 d | | Jovanovic et al. [233] | |  |
|  |  |  | 15 | PS | 51 | Opaque | Fr | 45 d | Daily | 3,330 ^c^ | 30 d | | INT | | - | 0, 30 d | | | 1.8 | > 30 d | | Jovanovic et al. [233] | |  |
|  |  |  | 15 | PS | 51 | Opaque | Fr | 45 d | Daily | 3,330 ^c^ | 30 d | | STO | | - | 0, 30 d | | | 2.07 | > 30 d | | Jovanovic et al. [233] | |  |
|  |  |  | 15 | PA | 11.7 | Opaque | Fr | 45 d | Daily | 3,330 ^c^ | 30 d | | STO | | - | 0, 30 d | | | 2.13 | > 30 d | | Jovanovic et al. [233] | |  |
|  |  |  | 15 | MDPE | 54.5 | Opaque | Fr | 45 d | Daily | 3,330 ^c^ | 30 d | | STO | | - | 0, 30 d | | | 2.47 | > 30 d | | Jovanovic et al. [233] | |  |
|  |  |  | 15 | LDPVC | 87.6 | Opaque | Fr | 45 d | Daily | 3,330 ^c^ | 30 d | | STO | | - | 0, 30 d | | | 5.4 | > 30 d | | Jovanovic et al. [233] | |  |
|  |  |  | 15 | LDPVC | 87.6 | Opaque | Fr | 45 d | Daily | 3,330 ^c^ | 30 d | | INT | | - | 0, 30 d | | | 9.27 | > 30 d | | Jovanovic et al. [233] | |  |
|  |  |  | 15 | MDPE | 54.6 | Opaque | Fr | 45 d | Daily | 3,330 ^c^ | 30 d | | INT | | - | 0, 30 d | | | 15.73 | > 30 d | | Jovanovic et al. [233] | |  |
|  |  |  | 15 | PA | 11.7 | Opaque | Fr | 45 d | Daily | 3,330 ^c^ | 30 d | | INT | | - | 0, 30 d | | | 34.27 | > 30 d | | Jovanovic et al. [233] | |  |

Sample sizes (n) have been included for convenience. MP shapes are reported as spheres (S), fibres (Fi), crystals (Cr), and fragments (Fr). Immediate ingestion (sampling at end of exposure duration) is reported in MP individual^-1^. Exposure and retention times are reported in minutes (min), hours (h), days (d), weeks (w), or months (mths). ‘Mix’ is representative of PES, PA and PP. Trophic levels have been verified using the well-established FishBase and SeaLifeBase databases. Values varying for Porter et al. [269] result from dosing either ^1^alongside marine snow, ^2^incorporated in marine snow, or ^3^alone. A dash (-) indicates that this information was not a component of the study; when it was but not reported, NM is used.

WO: Whole organism; NM: Not Mentioned; Flu: Fluorescent; ST: soft tissue; STO: stomach; INT: intestine; GI: gastrointestinal tract

^a^g ml^-1^; ^b^MPs g weight^-1^; ^c^mg kg^-1^; ^d^mg l^-1^; ^e^mg

*Values are the upper limit of ingestion rates reported. Full ingestion details for each treatment [211] or timeframe [174] were not reported.

S6 Table. Trophic transfer of microplastics (MPs) for marine organisms. Information on bioaccumulation and retention, following specific exposures to MPs under controlled laboratory conditions, are presented for each species and across two different trophic levels.

| **Trophic Levels** | **Experimental Design** | **Post-Feed Sampling** | ***C. maenas* Ingestion Reported** | **Retention** | **Reference** |
| --- | --- | --- | --- | --- | --- |
| 2 🡪 3.5 | 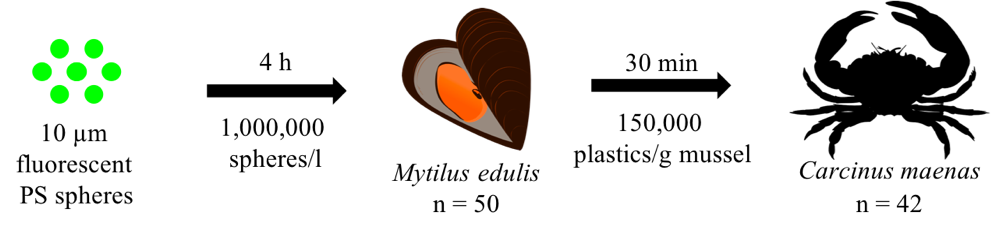 | 24 h | 100% ingested MPs | 14 days | Watts et al. [289] |
| 2 🡪 3.5 | 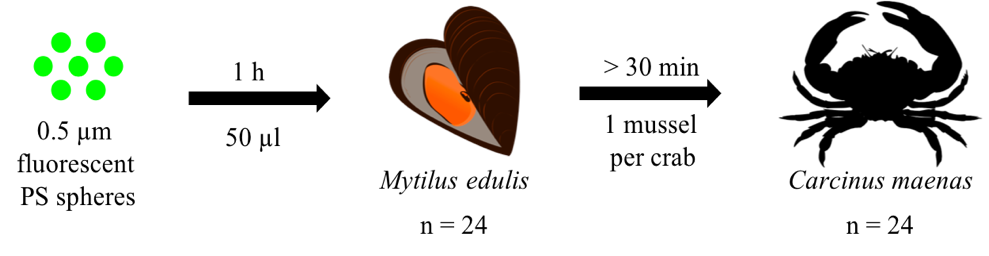 | 1 h | 1,025 ± 556 MPs ml^-1^ | < 21 days | Farrell and Nelson [218] |

Sample sizes (n) have been included in the experimental design. Trophic levels have been verified using the well-established SeaLifeBase database. Mussel and crab images acquired from: clipart-library.com

S7 Table. Experimental details of chemical additive laboratory exposures conducted with marine organisms. Bioaccumulation, following specific exposures to chemical additives under controlled laboratory conditions, are presented for each species, and across trophic levels.

|  |  |  |  |  | **Exposure Details** | | | | | | | | | | | |  | | |  | | |  | | |  | | |  | | |  |
| --- | --- | --- | --- | --- | --- | --- | --- | --- | --- | --- | --- | --- | --- | --- | --- | --- | --- | --- | --- | --- | --- | --- | --- | --- | --- | --- | --- | --- | --- | --- | --- | --- |
|  | **Class** | **Common Name** | ***Species Name*** | **n** | **Chemicals** | | | **Conc.**  **(ng g^-1^)** | **Dispersal Method** | | **Duration** | | **Dose** | | **Sampling Frequency** | | | **AI** | | | **Conc. Reported**  **(ng g^-1^)** | | | **Depuration Period** | | | **Retention** | | | **Reference** | | |
| **2** | Bivalvia | Mussel | *Mytilus edulis* | 8 | PAH: | Fluoranthene | 100 ^i^ | | | Alone | | 96 h | | Once | | - | | | DG | | | 80,460 | | | - | | | - | | | Magara et al. [247] | |
|  |  |  |  |  |  |  |  | | | Alongside PE | | 96 h | | Once | | - | | | DG | | | 41,500 | | | - | | | - | | | Magara et al. [247] | |
|  |  |  |  |  |  |  |  | | | On PE | | 96 h | | Once | | - | | | DG | | | 2,710 | | | - | | | - | | | Magara et al. [247] | |
|  |  |  | *Mytilus spp.* | 24 | PAH: | Fluoranthene | 30 ^i^ | | | Alone | | 7 d | | Once | | - | | | DG | | | 117,100 | | | 7 d | | | NM | | | Paul-Pont et al. [262] | |
|  |  |  |  |  |  |  |  | | | Alongside PS | | 7 d | | Once | | - | | | DG | | | 89,200 | | | 7 d | | | NM | | | Paul-Pont et al. [262] | |
|  |  | Clam | *Scrobicularia plana* | 170 | PAH: | Benzo[a]pyrene | 16870 | | | On LDPE | | 14 d | | Every  72 h | | - | | | T | | | 7.3 | | | - | | | - | | | O'Donovan et al. [260] | |
|  | Malacostraca | Amphipod | *Allorchestes compressa* | 5 | PBDE: | ∑PBDEs | 5 ^ii^ | | | Alongside MPs | | 72 h | | Once | | - | | | WO | | | 50 - 275 | | | 48 h | | | NM | | | Chua et al. [206] | |
|  |  |  |  |  |  |  |  | | | Alone | | 72 h | | Once | | - | | | WO | | | 2,000 – 2,250 | | | 48 h | | | NM | | | Chua et al. [206] | |
|  |  |  |  |  |  |  |  | | | On MPs | | 72 h | | Once | | - | | | WO | | | 2 - 13 | | | 48 h | | | NM | | | Chua et al. [206] | |
|  |  |  |  |  |  |  | 50 ^ii^ | | | Alongside MPs | | 72 h | | Once | | - | | | WO | | | 175 – 1,275 | | | 48 h | | | NM | | | Chua et al. [206] | |
|  |  |  |  |  |  |  |  | | | Alone | | 72 h | | Once | | - | | | WO | | | 3,500 – 7,900 | | | 48 h | | | NM | | | Chua et al. [206] | |
|  |  |  |  |  |  |  |  | | | On MPs | | 72 h | | Once | | - | | | WO | | | 0 - 70 | | | 48 h | | | NM | | | Chua et al. [206] | |
| **2.69** | Bivalvia | Mussel | *Mytilus galloprovincialis* | 180 | PAH: | Benzo[a]pyrene | 15000 | | | On LDPE | | 28 d | | Daily | | 7, 14, 28 d | | | DG | | | 30 | | | - | | | - | | | Pittura et al. [268] | |
|  |  |  |  |  |  |  | 0.15 ^i^ | | | Alone | | 28 d | | Daily | | 7, 14, 28 d | | | DG | | | 35 | | | - | | | - | | | Pittura et al. [268] | |
|  |  |  |  |  |  |  | 15000 | | | On LDPE | | 28 d | | Daily | | 7, 14, 28 d | | | Gills | | | 12 | | | - | | | - | | | Pittura et al. [268] | |
|  |  |  |  |  |  |  | 0.15 ^i^ | | | Alone | | 28 d | | Daily | | 7, 14, 28 d | | | Gills | | | 17 | | | - | | | - | | | Pittura et al. [268] | |
|  |  |  |  | 150 | PAH: | Pyrene | 200 - 260 | | | On PE and PS | | 7 d | | Daily | | - | | | DG | | | 470 | | | - | | | - | | | Avio et al. [186] | |
| **3.67** | Malacostraca | Lobster | *Nephrops norvegicus* | 7 | PCB: | ∑PCBs | 1350 ^ii^ | | | On PE | | 3 w | | M, W, F* | | - | | | T | | | 214 | | | - | | | - | | | Devriese et al. [215] | |
|  |  |  |  | 6 | PCB: | ∑PCBs | 1350 ^ii^ | | | On PS | | 3 w | | M, W, F* | | - | | | T | | | 154 | | | - | | | - | | | Devriese et al. [215] | |
|  |  |  |  | 8 | PCB: | ∑PCBs | 1350 ^ii^ | | | On PS (6 µm) | | 3 w | | M, W, F* | | - | | | T | | | 181 | | | - | | | - | | | Devriese et al. [215] | |
|  |  |  |  | 23 | PCB: | ∑PCBs | 1350 ^ii^ | | | Alone | | 3 w | | M, W, F* | | - | | | T | | | 229 – 2,940 | | | - | | | - | | | Devriese et al. [215] | |

Species have been organised by trophic level based on the well-established FishBase and SeaLifeBase databases. Exposure and retention times are reported in hours (h), days (d), weeks (w), or months (mths). Areas investigated (AI) for chemical additive contamination include digestive glands (DG), tissues (T) and whole organisms (WO). Sample sizes (n) have been given as well. Concentration reported is representative of contamination at end of exposure period and prior to the depuration period (if applicable).

NM: Not Mentioned; ^i^µg l^-1^; ^ii^ng; *Frequency of exposure to freshly dosed chemicals occurred on Monday, Wednesday and Friday of each week for the duration of the 3-week exposure [215].

S8 Table. Number of field and laboratory studies investigating microplastic (MP) uptake in marine organisms for each trophic level. Both total number of studies and number of studies utilising chemical confirmation of MPs (i.e. FTIR, Raman, polarised light microscopy) are presented for each trophic level from *in* *situ* studies. Laboratory exposure studies are also reported. This information was used when creating Fig 4 and 5 in the main manuscript. Please note these numbers will equate to a value larger than the reported number of studies included in this study due to studies investigating a multitude of trophic levels.

| Trophic  Level | Number of *in situ* Studies | Percent of *in situ* Studies  with Chemical Confirmation (n) | Number of Lab Exposure Studies |
| --- | --- | --- | --- |
| 1 | 1 | 0% (0) | 0 |
| 2 | 26 | 62% (16) | 12 |
| 2.1 | 2 | 50% (1) | 0 |
| 2.2 | 3 | 67% (2) | 0 |
| 2.3 | 7 | 57% (4) | 0 |
| 2.4 | 2 | 50% (1) | 0 |
| 2.5 | 6 | 83% (5) | 1 |
| 2.6 | 5 | 60% (3) | 1 |
| 2.7 | 4 | 50% (2) | 2 |
| 2.8 | 5 | 60% (3) | 2 |
| 2.9 | 4 | 25% (1) | 0 |
| 3 | 8 | 50% (4) | 1 |
| 3.1 | 23 | 65% (15) | 0 |
| 3.2 | 12 | 75% (9) | 0 |
| 3.3 | 19 | 74% (14) | 0 |
| 3.4 | 16 | 75% (12) | 0 |
| 3.5 | 18 | 56% (10) | 2 |
| 3.6 | 16 | 81% (13) | 0 |
| 3.7 | 16 | 75% (12) | 1 |
| 3.8 | 10 | 70% (7) | 0 |
| 3.9 | 8 | 75% (6) | 0 |
| 4 | 17 | 65% (11) | 0 |
| 4.1 | 12 | 83% (10) | 0 |
| 4.2 | 11 | 73% (8) | 0 |
| 4.3 | 11 | 73% (8) | 0 |
| 4.4 | 24 | 71% (17) | 0 |
| 4.5 | 17 | 59% (10) | 0 |

# Figures

**
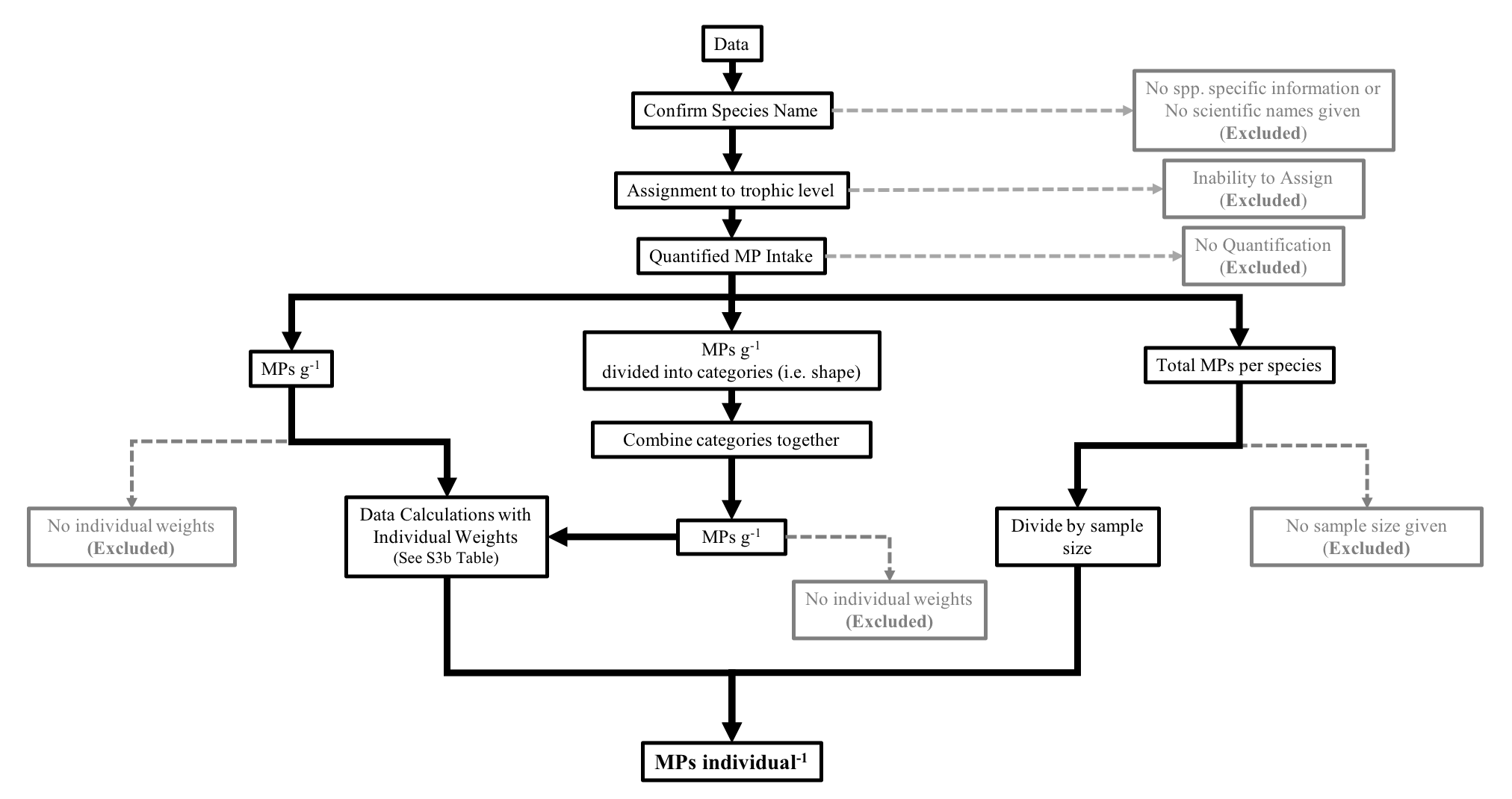
**

S1 Fig. Flow diagram outlining the standardisation of microplastic (MP) contamination data for marine organisms. Data on MPs were standardised to number of MPs per individual organism (MPs individual^-1^; i.e. body burden).

# References

1. Akhbarizadeh R, Moore F, Keshavarzi B. Investigating a probable relationship between microplastics and potentially toxic elements in fish muscles from northeast of Persian Gulf. Environ Pollut. 2018;232:154-63.

2. Allen AS, Seymour AC, Rittschof D. Chemoreception drives plastic consumption in a hard coral. Mar Pollut Bull. 2017;124(1):198-205.

3. Asmonaite G, Larsson K, Undeland I, Sturve J, Almroth BC. Size matters: Ingestion of relatively large microplastics contaminated with environmental pollutants posed little risk for fish health and fillet quality. Environ Sci Technol. 2018;52(24):14381-91.

4. Barboza LGA, Vieira LR, Branco V, Carvalho C, Guilherminol L. Microplastics increase mercury bioconcentration in gills and bioaccumulation in the liver, and cause oxidative stress and damage in *Dicentrarchus labrax* juveniles. Sci Rep. 2018;8:9.

5. Barboza LGA, Vieira LR, Branco V, Figueiredo N, Carvalho F, Carvalho C, et al. Microplastics cause neurotoxicity, oxidative damage and energy-related changes and interact with the bioaccumulation of mercury in the European seabass, Dicentrarchus labrax (Linnaeus, 1758). Aquat Toxicol. 2018;195:49-57.

6. Barboza LGA, Vieira LR, Guilhermino L. Single and combined effects of microplastics and mercury on juveniles of the European seabass (*Dicentrarchus labrax*): Changes in behavioural responses and reduction of swimming velocity and resistance time. Environ Pollut. 2018;236:1014-9.

7. Beiras R, Tato T. Microplastics do not increase toxicity of a hydrophobic organic chemical to marine plankton. Mar Pollut Bull. 2019;138:58-62.

8. Beiras R, Bellas J, Cachot J, Cormier B, Cousin X, Engwall M, et al. Ingestion and contact with polyethylene microplastics does not cause acute toxicity on marine zooplankton. J Hazard Mater. 2018;360:452-60.

9. Bessa F, Barria P, Neto JM, Frias J, Otero V, Sobral P, et al. Occurrence of microplastics in commercial fish from a natural estuarine environment. Mar Pollut Bull. 2018;128:575-84.

10. Besseling E, Foekema EM, van den Heuvel-Greve MJ, Koelmans AA. The effect of microplastic on the uptake of chemicals by the lugworm *Arenicola marina* (L.) under environmentally relevant exposure conditions. Environ Sci Technol. 2017;51(15):8795-804.

11. Bjorndal KA, Bolten AB, Lagueux CJ. Ingestion of marine debris by juvenile sea turtles in coastal Florida habitats. Mar Pollut Bull. 1994;28(3):154-8.

12. Bordbar L, Kapiris K, Kalogirou S, Anastasopoulou A. First evidence of ingested plastics by a high commercial shrimp species (*Plesionika narval*) in the eastern Mediterranean. Mar Pollut Bull. 2018;136:472-6.

13. Bour A, Avio CG, Gorbi S, Regoli F, Hylland K. Presence of microplastics in benthic and epibenthic organisms: Influence of habitat, feeding mode and trophic level. Environ Pollut. 2018;243:1217-25.

14. Boyle M, Limpus C. The stomach contents of post-hatchling green and loggerhead sea turtles in the southwest Pacific: an insight into habitat association. Mar Biol. 2008;155(2):233-41.

15. Brate ILN, Eidsvoll DP, Steindal CC, Thomas KV. Plastic ingestion by Atlantic cod (*Gadus morhua*) from the Norwegian coast. Mar Pollut Bull. 2016;112(1-2):105-10.

16. Browne MA, Dissanayake A, Galloway TS, Lowe DM, Thompson RC. Ingested microscopic plastic translocates to the circulatory system of the mussel, *Mytilus edulis* (L.). Environ Sci Technol. 2008;42(13):5026-31.

17. Budimir S, Setala O, Lehtiniemi M. Effective and easy to use extraction method shows low numbers of microplastics in offshore planktivorous fish from the northern Baltic Sea. Mar Pollut Bull. 2018;127:586-92.

18. Bussolaro D, Wright SL, Schnell S, Schirmer K, Bury NR, Arlt VM. Co-exposure to polystyrene plastic beads and polycyclic aromatic hydrocarbon contaminants in fish gill (RTgill-W1) and intestinal (RTgutGC) epithelial cells derived from rainbow trout (*Oncorhynchus mykiss*). Environ Pollut. 2019;248:706-14.

19. Cannon SME, Lavers JL, Figueiredo B. Plastic ingestion by fish in the Southern Hemisphere: A baseline study and review of methods. Mar Pollut Bull. 2016;107(1):286-91.

20. Carreras-Colom E, Constenla M, Soler-Membrives A, Cartes JE, Baeza M, Padros F, et al. Spatial occurrence and effects of microplastic ingestion on the deep-water shrimp Aristeus antennatus. Mar Pollut Bull. 2018;133:44-52.

21. Caruso G, Peda C, Cappello S, Leonardi M, La Ferla R, Lo Giudice A, et al. Effects of microplastics on trophic parameters, abundance and metabolic activities of seawater and fish gut bacteria in mesocosm conditions. Environ Sci Pollut Res. 2018;25(30):30067-83.

22. Chagnon C, Thiel M, Antunes J, Ferreira JL, Sobral P, Ory NC. Plastic ingestion and trophic transfer between Easter Island flying fish (*Cheilopogon rapanouiensis*) and yellowfin tuna (*Thunnus albacares*) from Rapa Nui (Easter Island). Environ Pollut. 2018;243:127-33.

23. Chapron L, Peru E, Engler A, Ghiglione JF, Meistertzheim AL, Pruski AM, et al. Macro- and microplastics affect cold-water corals growth, feeding and behaviour. Sci Rep. 2018;8:8.

24. Cole M, Galloway TS. Ingestion of nanoplastics and microplastics by Pacific oyster larvae. Environ Sci Technol. 2015;49(24):14625-32.

25. Cole M, Lindeque PK, Fileman E, Clark J, Lewis C, Halsband C, et al. Microplastics alter the properties and sinking rates of zooplankton faecal pellets. Environ Sci Technol. 2016;50(6):3239-46.

26. Compa M, Ventero A, Iglesias M, Deudero S. Ingestion of microplastics and natural fibres in *Sardina pilchardus* (Walbaum, 1792) and *Engraulis encrasicolus* (Linnaeus, 1758) along the Spanish Mediterranean coast. Mar Pollut Bull. 2018;128:89-96.

27. Courtene-Jones W, Quinn B, Gary SF, Mogg AOM, Narayanaswamy BE. Microplastic pollution identified in deep-sea water and ingested by benthic invertebrates in the Rockall Trough, North Atlantic Ocean. Environ Pollut. 2017;231:271-80.

28. Courtene-Jones W, Quinn B, Murphy F, Gary SF, Narayanaswamy BE. Optimisation of enzymatic digestion and validation of specimen preservation methods for the analysis of ingested microplastics. Anal Methods. 2017;9(9):1437-45.

29. Courtene-Jones W, Quinn B, Ewins C, Gary SF, Narayanaswamy BE. Consistent microplastic ingestion by deep-sea invertebrates over the last four decades (1976-2015), a study from the North East Atlantic. Environ Pollut. 2019;244:503-12.

30. Cunha C, Faria M, Nogueira N, Ferreira A, Cordeiro N. Marine vs freshwater microalgae exopolymers as biosolutions to microplastics pollution. Environ Pollut. 2019;249:372-80.

31. Dantas DV, Ribeiro CIR, Frischknecht CDA, Machado R, Farias EGG. Ingestion of plastic fragments by the Guri sea catfish *Genidens genidens* (Cuvier, 1829) in a subtropical coastal estuarine system. Environ Sci Pollut Res. 2019;26(8):8344-51.

32. Davarpanah E, Guilhermino L. Single and combined effects of microplastics and copper on the population growth of the marine microalgae *Tetraselmis chuii*. Estuar Coast Shelf Sci. 2015;167:269-75.

33. Davison P, Asch RG. Plastic ingestion by mesopelagic fishes in the north pacific subtropical gyre. Marine Ecology Progress Series. 2011;432:173-80.

34. Dawson AL, Kawaguchi S, King CK, Townsend KA, King R, Huston WM, et al. Turning microplastics into nanoplastics through digestive fragmentation by Antarctic krill. Nat Commun. 2018;9:8.

35. Dawson A, Huston W, Kawaguchi S, King C, Cropp R, Wild S, et al. Uptake and depuration kinetics influence microplastic bioaccumulation and toxicity in Antarctic krill (*Euphausia superba*). Environ Sci Technol. 2018;52(5):3195-201.

36. de Orte MR, Clowez S, Caldeira K. Response of bleached and symbiotic sea anemones to plastic microfiber exposure. Environ Pollut. 2019;249:512-7.

37. de Sa LC, Luis LG, Guilhermino L. Effects of microplastics on juveniles of the common goby (*Pomatoschistus microps*): Confusion with prey, reduction of the predatory performance and efficiency, and possible influence of developmental conditions. Environ Pollut. 2015;196:359-62.

38. Detree C, Gallardo-Escarate C. Single and repetitive microplastics exposures induce immune system modulation and homeostasis alteration in the edible mussel *Mytilus galloprovincialis*. Fish Shellfish Immunol. 2018;83:52-60.

39. Ding JF, Li JX, Sun CJ, He CF, Jiang FH, Gao FL, et al. Separation and identification of microplastics in digestive system of bivalves. Chin J Anal Chem. 2018;46(5):690-7.

40. Ding JF, Li JX, Sun CJ, Jiang FH, Ju P, Qu LY, et al. Detection of microplastics in local marine organisms using a multi-technology system. Anal Methods. 2019;11(1):78-87.

41. Duncan EM, Arrowsmith J, Bain C, Broderick AC, Lee J, Metcalfe K, et al. The true depth of the Mediterranean plastic problem: Extreme microplastic pollution on marine turtle nesting beaches in Cyprus. Mar Pollut Bull. 2018;136:334-40.

42. Egbeocha CO, Malek S, Emenike CU, Milow P. Feasting on microplastics: Ingestion by and effects on marine organisms. Aquat Biol. 2018;27:93-106.

43. Espinosa C, Beltran JMG, Esteban MA, Cuesta A. In vitro effects of virgin microplastics on fish head-kidney leucocyte activities. Environ Pollut. 2018;235:30-8.

44. Fang C, Zheng RH, Zhang YS, Hong FK, Mu JL, Chen MY, et al. Microplastic contamination in benthic organisms from the Arctic and sub-Arctic regions. Chemosphere. 2018;209:298-306.

45. Fernandez B, Albentosa M. Insights into the uptake, elimination and accumulation of microplastics in mussel. Environ Pollut. 2019;249:321-9.

46. Ferreira GVB, Barletta M, Lima ARA, Dantas DV, Justino AKS, Costa MF. Plastic debris contamination in the life cycle of Acoupa weakfish (*Cynoscion acoupa*) in a tropical estuary. ICES J Mar Sci. 2016;73(10):2695-707.

47. Ferreira P, Fonte E, Soares ME, Carvalho F, Guilhermino L. Effects of multi-stressors on juveniles of the marine fish *Pomatoschistus microps*: Gold nanoparticles, microplastics and temperature. Aquat Toxicol. 2016;170:89-103.

48. Ferreira GVB, Barletta M, Lima ARA. Use of estuarine resources by top predator fishes. How do ecological patterns affect rates of contamination by microplastics? Sci Total Environ. 2019;655:292-304.

49. Floren HP, Shugart GW. Plastic in Cassin's Auklets (*Ptychoramphus aleuticus*) from the 2014 stranding on the Northeast Pacific Coast. Mar Pollut Bull. 2017;117(1-2):496-8.

50. Fonte E, Ferreira P, Guilhermino L. Temperature rise and microplastics interact with the toxicity of the antibiotic cefalexin to juveniles of the common goby (*Pomatoschistus microps*): Post-exposure predatory behaviour, acetylcholinesterase activity and lipid peroxidation. Aquat Toxicol. 2016;180:173-85.

51. Fossi MC, Coppola D, Baini M, Giannetti M, Guerranti C, Marsili L, et al. Large filter feeding marine organisms as indicators of microplastic in the pelagic environment: The case studies of the Mediterranean basking shark (*Cetorhinus maximus*) and fin whale (*Balaenoptera physalus*). Mar Environ Res. 2014;100:17-24.

52. Fossi MC, Marsili L, Baini M, Giannetti M, Coppola D, Guerranti C, et al. Fin whales and microplastics: The Mediterranean Sea and the Sea of Cortez scenarios. Environ Pollut. 2016;209:68-78.

53. Fossi MC, Romeo T, Baini M, Panti C, Marsili L, Campani T, et al. Plastic debris occurrence, convergence areas and fin whales feeding ground in the mediterranean marine protected area pelagos sanctuary: A modeling approach. Front Mar Sci. 2017;4:15.

54. Franzellitti S, Capolupo M, Wathsala R, Valbonesi P, Fabbri E. The multixenobiotic resistance system as a possible protective response triggered by microplastic ingestion in Mediterranean mussels (*Mytilus galloprovincialis*): Larvae and adult stages. Comp Biochem Physiol C-Toxicol Pharmacol. 2019;219:50-8.

55. Galgani L, Engel A, Rossi C, Donati A, Loiselle SA. Polystyrene microplastics increase microbial release of marine chromophoric dissolved organic matter in microcosm experiments. Sci Rep. 2018;8:11.

56. Gardon T, Reisser C, Soyez C, Quillien V, Le Moullac G. Microplastics affect energy balance and gametogenesis in the pearl oyster *Pinctada margaritifera*. Environ Sci Technol. 2018;52(9):5277-86.

57. Garrido S, Linares M, Campillo JA, Albentosa M. Effect of microplastics on the toxicity of chlorpyrifos to the microalgae *Isochrysis galbana*, clone t-ISO. Ecotox Environ Safe. 2019;173:103-9.

58. Gaspar TR, Chi RJ, Parrow MW, Ringwood AH. Cellular bioreactivity of micro- and nano-plastic particles in oysters. Front Mar Sci. 2018;5:8.

59. Gassel M, Rochman CM. The complex issue of chemicals and microplastic pollution: A case study in North Pacific lanternfish. Environ Pollut. 2019;248:1000-9.

60. Gebhardt C, Forster S. Size-selective feeding of Arenicola marina promotes long-term burial of microplastic particles in marine sediments. Environ Pollut. 2018;242:1777-86.

61. Goncalves C, Martins M, Sobral P, Costa PM, Costa MH. An assessment of the ability to ingest and excrete microplastics by filter-feeders: A case study with the Mediterranean mussel. Environ Pollut. 2019;245:600-6.

62. Granby K, Rainieri S, Rasmussen RR, Kotterman MJJ, Sloth JJ, Cederberg TL, et al. The influence of microplastics and halogenated contaminants in feed on toxicokinetics and gene expression in European seabass (*Dicentrarchus labrax*). Environ Res. 2018;164:430-43.

63. Green DS. Effects of microplastics on European flat oysters, Ostrea edulis and their associated benthic communities. Environ Pollut. 2016;216:95-103.

64. Green DS, Boots B, Sigwart J, Jiang S, Rocha C. Effects of conventional and biodegradable microplastics on a marine ecosystem engineer (*Arenicola marina*) and sediment nutrient cycling. Environ Pollut. 2016;208:426-34.

65. Green DS, Boots B, O'Connor NE, Thompson R. Microplastics affect the ecological functioning of an important biogenic habitat. Environ Sci Technol. 2017;51(1):68-77.

66. Green DS, Colgan TJ, Thompson RC, Carolan JC. Exposure to microplastics reduces attachment strength and alters the haemolymph proteome of blue mussels (*Mytilus edulis*). Environ Pollut. 2019;246:423-34.

67. Guebert-Bartholo F, Barletta M, Costa M, Monteiro-Filho E. Using gut contents to assess foraging patterns of juvenile green turtles *Chelonia mydas* in the Paranaguá Estuary, Brazil. Endanger Species Res. 2011;13(2):131-43.

68. Gutow L, Eckerlebe A, Gimenez L, Saborowski R. Experimental evaluation of seaweeds as a vector for microplastics into marine food webs. Environ Sci Technol. 2016;50(2):915-23.

69. Gutow L, Bartl K, Saborowski R, Beermann J. Gastropod pedal mucus retains microplastics and promotes the uptake of particles by marine periwinkles. Environ Pollut. 2019;246:688-96.

70. Guven O, Bach L, Munk P, Dinh KV, Mariani P, Nielsen TG. Microplastic does not magnify the acute effect of PAH pyrene on predatory performance of a tropical fish (*Lates calcarifer*). Aquat Toxicol. 2018;198:287-93.

71. Hall NM, Berry KLE, Rintoul L, Hoogenboom MO. Microplastic ingestion by scleractinian corals. Mar Biol. 2015;162(3):725-32.

72. Halstead JE, Smith JA, Carter EA, Lay PA, Johnston EL. Assessment tools for microplastics and natural fibres ingested by fish in an urbanised estuary. Environ Pollut. 2018;234:552-61.

73. Hankins C, Duffy A, Drisco K. Scleractinian coral microplastic ingestion: Potential calcification effects, size limits, and retention. Mar Pollut Bull. 2018;135:587-93.

74. Hermabessiere L, Himber C, Boricaud B, Kazour M, Amara R, Cassone AL, et al. Optimization, performance, and application of a pyrolysis-GC/MS method for the identification of microplastics. Anal Bioanal Chem. 2018;410(25):6663-76.

75. Hermsen E, Pompe R, Besseling E, Koelmans AA. Detection of low numbers of microplastics in North Sea fish using strict quality assurance criteria. Mar Pollut Bull. 2017;122(1-2):253-8.

76. Horn D, Miller M, Anderson S, Steele C. Microplastics are ubiquitous on California beaches and enter the coastal food web through consumption by Pacific mole crabs. Mar Pollut Bull. 2019;139:231-7.

77. Jacob H, Gilson A, Lanctot C, Besson M, Metian M, Lecchini D. No effect of polystyrene microplastics on foraging activity and survival in a post-larvae coral-reef fish, *Acanthurus triostegus*. Bull Environ Contam Toxicol. 2019;102(4):457-61.

78. Jamieson AJ, Brooks LSR, Reid WDK, Piertney SB, Narayanaswamy BE, Linley TD. Microplastics and synthetic particles ingested by deep-sea amphipods in six of the deepest marine ecosystems on earth. R Soc Open Sci. 2019;6(2):11.

79. Jeong CB, Kang HM, Lee MC, Kim DH, Han J, Hwang DS, et al. Adverse effects of microplastics and oxidative stress-induced MAPK/ Nrf2 pathway-mediated defense mechanisms in the marine copepod *Paracyclopina nana*. Sci Rep. 2017;7:11.

80. Jung MR, Balazs GH, Work TM, Jones TT, Orski SV, Rodriguez CV, et al. Polymer identification of plastic debris ingested by pelagic-phase sea turtles in the central pacific. Environ Sci Technol. 2018;52(20):11535-44.

81. Jung MR, Horgen FD, Orski SV, Rodriguez CV, Beers KL, Balazs GH, et al. Validation of ATR FT-IR to identify polymers of plastic marine debris, including those ingested by marine organisms. Mar Pollut Bull. 2018;127:704-16.

82. Karami A, Golieskardi A, Choo CK, Larat V, Karbalaei S, Salamatinia B. Microplastic and mesoplastic contamination in canned sardines and sprats. Sci Total Environ. 2018;612:1380-6.

83. Khan MB, Prezant RS. Microplastic abundances in a mussel bed and ingestion by the ribbed marsh mussel geukensia demissa. Mar Pollut Bull. 2018;130:67-75.

84. Khan FR, Boyle D, Chang E, Bury NR. Do polyethylene microplastic beads alter the intestinal uptake of Ag in rainbow trout (*Oncorhynchus mykiss*)? Analysis of the MP vector effect using in vitro gut sacs. Environ Pollut. 2017;231:200-6.

85. Kolandhasamy P, Su L, Li JN, Qu XY, Jabeen K, Shi HH. Adherence of microplastics to soft tissue of mussels: A novel way to uptake microplastics beyond ingestion. Sci Total Environ. 2018;610:635-40.

86. Kuhn S, van Werven B, van Oyen A, Meijboom A, Rebolledo ELB, van Franeker JA. The use of potassium hydroxide (KOH) solution as a suitable approach to isolate plastics ingested by marine organisms. Mar Pollut Bull. 2017;115(1-2):86-90.

87. Kumar VE, Ravikumar G, Jeyasanta KI. Occurrence of microplastics in fishes from two landing sites in Tuticorin, South east coast of India. Mar Pollut Bull. 2018;135:889-94.

88. La Beur L, Henry LA, Kazanidis G, Hennige S, McDonald A, Shaver MP, et al. Baseline assessment of marine litter and microplastic ingestion by cold-water coral reef benthos at the east mingulay marine protected area (sea of the hebrides, western scotland). Front Mar Sci. 2019;6:13.

89. Leung J, Chan KYK. Microplastics reduced posterior segment regeneration rate of the polychaete Perinereis aibuhitensis. Mar Pollut Bull. 2018;129(2):782-6.

90. Li HX, Getzinger GJ, Ferguson PL, Orihuela B, Zhu M, Rittschof D. Effects of toxic leachate from commercial plastics on larval survival and settlement of the barnacle *Amphibalanus amphitrite*. Environ Sci Technol. 2016;50(2):924-31.

91. Li HX, Ma LS, Lin L, Ni ZX, Xu XR, Shi HH, et al. Microplastics in oysters Saccostrea cucullata along the Pearl River Estuary, China. Environ Pollut. 2018;236:619-25.

92. Liboiron M, Liboiron F, Wells E, Richard N, Zahara A, Mather C, et al. Low plastic ingestion rate in Atlantic cod (*Gadus morhua*) from newfoundland destined for human consumption collected through citizen science methods. Mar Pollut Bull. 2016;113(1-2):428-37.

93. Liboiron M, Melvin J, Richard N, Saturno J, Ammendolia J, Liboiron F, et al. Low incidence of plastic ingestion among three fish species significant for human consumption on the island of Newfoundland, Canada. Mar Pollut Bull. 2019;141:244-8.

94. Lo HKA, Chan KYK. Negative effects of microplastic exposure on growth and development of *Crepidula onyx*. Environ Pollut. 2018;233:588-95.

95. Long M, Paul-Pont I, Hegaret H, Moriceau B, Lambert C, Huvet A, et al. Interactions between polystyrene microplastics and marine phytoplankton lead to species-specific hetero-aggregation. Environ Pollut. 2017;228:454-63.

96. Luan LP, Wang X, Zheng H, Liu LQ, Luo XX, Li FM. Differential toxicity of functionalized polystyrene microplastics to clams (*Meretrix meretrix*) at three key development stages of life history. Mar Pollut Bull. 2019;139:346-54.

97. Luis LG, Ferreira P, Fonte E, Oliveira M, Guilhermino L. Does the presence of microplastics influence the acute toxicity of chromium(VI) to early juveniles of the common goby (*Pomatoschistus microps*)? A study with juveniles from two wild estuarine populations. Aquat Toxicol. 2015;164:163-74.

98. M'Rabet C, Pringault O, Zmerli-Triki H, Ben Gharbia H, Couet D, Yahia OKD. Impact of two plastic-derived chemicals, the Bisphenol A and the di-2-ethylhexyl phthalate, exposure on the marine toxic dinoflagellate *Alexandrium pacificum*. Mar Pollut Bull. 2018;126:241-9.

99. Macali A, Semenov A, Venuti V, Crupi V, D'Amico F, Rossi B, et al. Episodic records of jellyfish ingestion of plastic items reveal a novel pathway for trophic transference of marine litter. Sci Rep. 2018;8:5.

100. Mao YF, Ai HN, Chen Y, Zhang ZY, Zeng P, Kang L, et al. Phytoplankton response to polystyrene microplastics: Perspective from an entire growth period. Chemosphere. 2018;208:59-68.

101. Martinez-Gomez C, Leon VM, Calles S, Gomariz-Olcina M, Vethaak AD. The adverse effects of virgin microplastics on the fertilization and larval development of sea urchins. Mar Environ Res. 2017;130:69-76.

102. Mathalon A, Hill P. Microplastic fibers in the intertidal ecosystem surrounding Halifax Harbor, Nova Scotia. Mar Pollut Bull. 2014;81(1):69-79.

103. Mecozzi M, Pietroletti M, Monakhova YB. Ftir spectroscopy supported by statistical techniques for the structural characterization of plastic debris in the marine environment: Application to monitoring studies. Mar Pollut Bull. 2016;106(1-2):155-61.

104. Messinetti S, Mercurio S, Parolini M, Sugni M, Pennati R. Effects of polystyrene microplastics on early stages of two marine invertebrates with different feeding strategies. Environ Pollut. 2018;237:1080-7.

105. Messinetti S, Mercurio S, Scari G, Pennati A, Pennati R. Ingested microscopic plastics translocate from the gut cavity of juveniles of the ascidian *Ciona intestinalis*. Eur Zool J. 2019;86(1):189-95.

106. Miranda DD, de Carvalho-Souza GF. Are we eating plastic-ingesting fish? Mar Pollut Bull. 2016;103(1-2):109-14.

107. Naidoo T, Goordiyal K, Glassom D. Are nitric acid (HNO_3_) digestions efficient in isolating microplastics from juvenile fish? Water Air Soil Pollut. 2017;228(12):11.

108. Naidu SA. Preliminary study and first evidence of presence of microplastics and colorants in green mussel, *Perna viridis* (Linnaeus, 1758), from southeast coast of India. Mar Pollut Bull. 2019;140:416-22.

109. Naidu SA, Rao VR, Ramu K. Microplastics in the benthic invertebrates from the coastal waters of Kochi, Southeastern Arabian Sea. Environ Geochem Health. 2018;40(4):1377-83.

110. Naji A, Nuri M, Vethaak AD. Microplastics contamination in molluscs from the northern part of the persian gulf. Environ Pollut. 2018;235:113-20.

111. Nel HA, Froneman PW. Presence of microplastics in the tube structure of the reef-building polychaete *Gunnarea gaimardi* (Quatrefages 1848). Afr J Mar Sci. 2018;40(1):87-9.

112. Nobre CR, Santana MFM, Maluf A, Cortez FS, Cesar A, Pereira CDS, et al. Assessment of microplastic toxicity to embryonic development of the sea urchin *Lytechinus variegatus* (Echinodermata: Echinoidea). Mar Pollut Bull. 2015;92(1-2):99-104.

113. Okubo N, Takahashi S, Nakano Y. Microplastics disturb the anthozoan-algae symbiotic relationship. Mar Pollut Bull. 2018;135:83-9.

114. Oliviero M, Tato T, Schiavo S, Fernandez V, Manzo S, Beiras R. Leachates of micronized plastic toys provoke embryotoxic effects upon sea urchin *Paracentrotus lividus*. Environ Pollut. 2019;247:706-15.

115. Ory N, Chagnon C, Felix F, Fernandez C, Ferreira JL, Gallardo C, et al. Low prevalence of microplastic contamination in planktivorous fish species from the southeast Pacific Ocean. Mar Pollut Bull. 2018;127:211-6.

116. Peda C, Caccamo L, Fossi MC, Gai F, Andaloro F, Genovese L, et al. Intestinal alterations in European sea bass *Dicentrarchus labrax* (Linnaeus, 1758) exposed to microplastics: Preliminary results. Environ Pollut. 2016;212:251-6.

117. Peters CA, Hendrickson E, Minor EC, Schreiner K, Halbur J, Bratton SP. Pyr-GC/MS analysis of microplastics extracted from the stomach content of benthivore fish from the Texas Gulf Coast. Mar Pollut Bull. 2018;137:91-5.

118. Pham CK, Rodriguez Y, Dauphin A, Carrico R, Frias J, Vandeperre F, et al. Plastic ingestion in oceanic-stage loggerhead sea turtles (*Caretta caretta*) off the North Atlantic subtropical gyre. Mar Pollut Bull. 2017;121(1-2):222-9.

119. Phuong NN, Zalouk-Vergnoux A, Kamari A, Mouneyrac C, Amiard F, Poirier L, et al. Quantification and characterization of microplastics in blue mussels (*Mytilus edulis*): Protocol setup and preliminary data on the contamination of the French Atlantic coast. Environ Sci Pollut Res. 2018;25(7):6135-44.

120. Pozo K, Gomez V, Torres M, Vera L, Nunez D, Oyarzun P, et al. Presence and characterization of microplastics in fish of commercial importance from the Biobío region in central Chile. Mar Pollut Bull. 2019;140:315-9.

121. Prata JC, Lavorante B, Montenegro M, Guilhermino L. Influence of microplastics on the toxicity of the pharmaceuticals procainamide and doxycycline on the marine microalgae *Tetraselmis chuii*. Aquat Toxicol. 2018;197:143-52.

122. Rebolledo ELB, Van Franeker JA, Jansen OE, Brasseur S. Plastic ingestion by harbour seals (*Phoca vitulina)* in the Netherlands. Mar Pollut Bull. 2013;67(1-2):200-2.

123. Reichert J, Schellenberg J, Schubert P, Wilke T. Responses of reef building corals to microplastic exposure. Environ Pollut. 2018;237:955-60.

124. Reichert J, Arnold AL, Hoogenboom MO, Schubert P, Wilke T. Impacts of microplastics on growth and health of hermatypic corals are species-specific. Environ Pollut. 2019;254:9.

125. Remy F, Collard F, Gilbert B, Compere P, Eppe G, Lepoint G. When microplastic is not plastic: The ingestion of artificial cellulose fibers by macrofauna living in seagrass macrophytodetritus. Environ Sci Technol. 2015;49(18):11158-66.

126. Renzi M, Blaskovic A, Bernardi G, Russo GF. Plastic litter transfer from sediments towards marine trophic webs: A case study on holothurians. Mar Pollut Bull. 2018;135:376-85.

127. Renzi M, Guerranti C, Blaslovic A. Microplastic contents from maricultured and natural mussels. Mar Pollut Bull. 2018;131:248-51.

128. Ribeiro F, Garcia AR, Pereira BP, Fonseca M, Mestre NC, Fonseca TG, et al. Microplastics effects in *Scrobicularia plana*. Mar Pollut Bull. 2017;122(1-2):379-91.

129. Rist S, Steensgaard IM, Guven O, Nielsen TG, Jensen LH, Moller LF, et al. The fate of microplastics during uptake and depuration phases in a blue mussel exposure system. Environ Toxicol Chem. 2019;38(1):99-105.

130. Rivera-Hernandez JR, Fernandez B, Santos-Echeandia J, Garrido S, Morante M, Santos P, et al. Biodynamics of mercury in mussel tissues as a function of exposure pathway: Natural vs microplastic routes. Sci Total Environ. 2019;674:412-23.

131. Roch S, Brinker A. Rapid and efficient method for the detection of microplastic in the gastrointestinal tract of fishes. Environ Sci Technol. 2017;51(8):4522-30.

132. Rochman CM, Lewison RL, Eriksen M, Allen H, Cook A-M, Teh SJ. Polybrominated diphenyl ethers (PBDEs) in fish tissue may be an indicator of plastic contamination in marine habitats. Sci Total Environ. 2014;476:622-33.

133. Rochman CM, Tahir A, Williams SL, Baxa DV, Lam R, Miller JT, et al. Anthropogenic debris in seafood: Plastic debris and fibers from textiles in fish and bivalves sold for human consumption. Sci Rep. 2015;5:10.

134. Romeo T, Peda C, Fossi MC, Andaloro F, Battaglia P. First record of plastic debris in the stomach of mediterranean lanternfishes. Acta Adriat. 2016;57(1):115-23.

135. Rosas-Luis R. Description of plastic remains found in the stomach contents of the jumbo squid dosidicus gigas landed in ecuador during 2014. Mar Pollut Bull. 2016;113(1-2):302-5.

136. Santana MFM, Ascer LG, Custodio MR, Moreira FT, Turra A. Microplastic contamination in natural mussel beds from a brazilian urbanized coastal region: Rapid evaluation through bioassessment. Mar Pollut Bull. 2016;106(1-2):183-9.

137. Santana MFM, Moreira FT, Turra A. Trophic transference of microplastics under a low exposure scenario: Insights on the likelihood of particle cascading along marine food-webs. Mar Pollut Bull. 2017;121(1-2):154-9.

138. Santana MFM, Moreira FT, Pereira CDS, Abessa DMS, Turra A. Continuous exposure to microplastics does not cause physiological effects in the cultivated mussel Perna perna. Arch Environ Contam Toxicol. 2018;74(4):594-604.

139. Savoca S, Capillo G, Mancuso M, Bottari T, Crupi R, Branca C, et al. Microplastics occurrence in the Tyrrhenian waters and in the gastrointestinal tract of two congener species of seabreams. Environ Toxicol Pharmacol. 2019;67:35-41.

140. Schuyler Q, Hardesty BD, Wilcox C, Townsend K. To eat or not to eat? Debris selectivity by marine turtles. PLoS One. 2012;7(7).

141. Schuyler Q, Hardesty BD, Wilcox C, Townsend K. Global analysis of anthropogenic debris ingestion by sea turtles. Conservation biology. 2014;28(1):129-39.

142. Seuront L. Microplastic leachates impair behavioural vigilance and predator avoidance in a temperate intertidal gastropod. Biol Lett. 2018;14(11):5.

143. Silva P, Nobre CR, Resaffe P, Pereira CDS, Gusmao F. Leachate from microplastics impairs larval development in brown mussels. Water Res. 2016;106:364-70.

144. Sjollema SB, Redondo-Hasselerharm P, Leslie HA, Kraak MHS, Vethaak AD. Do plastic particles affect microalgal photosynthesis and growth? Aquat Toxicol. 2016;170:259-61.

145. Smith LE. Plastic ingestion by scyliorhinus canicula trawl captured in the north sea. Mar Pollut Bull. 2018;130:6-7.

146. Sun XX, Li QJ, Zhu ML, Liang JH, Zheng S, Zhao YF. Ingestion of microplastics by natural zooplankton groups in the northern South China Sea. Mar Pollut Bull. 2017;115(1-2):217-24.

147. Sun XX, Liang JH, Zhu ML, Zhao YF, Zhang B. Microplastics in seawater and zooplankton from the Yellow Sea. Environ Pollut. 2018;242:585-95.

148. Sun XX, Liu T, Zhu ML, Liang JH, Zhao YF, Zhang B. Retention and characteristics of microplastics in natural zooplankton taxa from the East China Sea. Sci Total Environ. 2018;640:232-42.

149. Sun XM, Chen BJ, Li QF, Liu N, Xia B, Zhu L, et al. Toxicities of polystyrene nano- and microplastics toward marine bacterium *Halomonas alkaliphila*. Sci Total Environ. 2018;642:1378-85.

150. Sundbaek KB, Koch IDW, Villaro CG, Rasmussen NS, Holdt SL, Hartmann NB. Sorption of fluorescent polystyrene microplastic particles to edible seaweed *Fucus vesiculosus*. J Appl Phycol. 2018;30(5):2923-7.

151. Sussarellu R, Suquet M, Thomas Y, Lambert C, Fabioux C, Pernet MEJ, et al. Oyster reproduction is affected by exposure to polystyrene microplastics. Proc Natl Acad Sci U S A. 2016;113(9):2430-5.

152. Syakti AD, Jaya JV, Rahman A, Hidayati NV, Raza'i TS, Idris F, et al. Bleaching and necrosis of staghorn coral (*Acropora formosa*) in laboratory assays: Immediate impact of LDPE microplastics. Chemosphere. 2019;228:528-35.

153. Syberg K, Nielsen A, Khan FR, Banta GT, Palmqvist A, Jepsen PM. Microplastic potentiates triclosan toxicity to the marine copepod *Acartia tonsa* (Dana). J Toxicol Env Health Part A. 2017;80(23-24):1369-71.

154. Tang J, Ni XZ, Zhou Z, Wang LG, Lin SJ. Acute microplastic exposure raises stress response and suppresses detoxification and immune capacities in the scleractinian coral *Pocillopora damicornis*. Environ Pollut. 2018;243:66-74.

155. Thiagarajan V, Iswarya V, Julian PA, Seenivasan R, Chandrasekaran N, Mukherjee A. Influence of differently functionalized polystyrene microplastics on the toxic effects of P25 TiO2 NPs towards marine algae *Chlorella* sp. Aquat Toxicol. 2019;207:208-16.

156. Thushari GGN, Senevirathna JDM, Yakupitiyage A, Chavanich S. Effects of microplastics on sessile invertebrates in the eastern coast of Thailand: An approach to coastal zone conservation. Mar Pollut Bull. 2017;124(1):349-55.

157. Tosetto L, Williamson JE, Brown C. Trophic transfer of microplastics does not affect fish personality. Anim Behav. 2017;123:159-67.

158. Van Cauwenberghe L, Janssen CR. Microplastics in bivalves cultured for human consumption. Environ Pollut. 2014;193:65-70.

159. van Franeker JA, Rebolledo ELB, Hesse E, Ijsseldijk LL, Kuhn S, Leopold M, et al. Plastic ingestion by harbour porpoises phocoena phocoena in the netherlands: Establishing a standardised method. Ambio. 2018;47(4):387-97.

160. von Moos N, Burkhardt-Holm P, Kohler A. Uptake and effects of microplastics on cells and tissue of the blue mussel *Mytilus edulis* L. After an experimental exposure. Environ Sci Technol. 2012;46(20):11327-35.

161. Wang ZM, Wagner J, Ghosal S, Bedi G, Wall S. SEM/EDS and opticalmicroscopy analyses of microplastics in ocean trawl and fish guts. Sci Total Environ. 2017;603:616-26.

162. Wang Y, Zhang D, Zhang MX, Mu JL, Ding GH, Mao Z, et al. Effects of ingested polystyrene microplastics on brine shrimp, *Artemia parthenogenetica*. Environ Pollut. 2019;244:715-22.

163. Wang J, Lu L, Wang MX, Jiang T, Liu XS, Ru SG. Typhoons increase the abundance of microplastics in the marine environment and cultured organisms: A case study in Sanggou Bay, China. Sci Total Environ. 2019;667:1-8.

164. Watts AJR, Urbina MA, Corr S, Lewis C, Galloway TS. Ingestion of plastic microfibers by the crab *Carcinus maenas* and its effect on food consumption and energy balance. Environ Sci Technol. 2015;49(24):14597-604.

165. Watts AJR, Urbina MA, Goodhead R, Moger J, Lewis C, Galloway TS. Effect of microplastic on the gills of the shore crab *Carcinus maenas*. Environ Sci Technol. 2016;50(10):5364-9.

166. Welden NAC, Cowie PR. Environment and gut morphology influence microplastic retention in langoustine, *Nephrops norvegicus*. Environ Pollut. 2016;214:859-65.

167. Welden NAC, Cowie PR. Long-term microplastic retention causes reduced body condition in the langoustine, *Nephrops norvegicus*. Environ Pollut. 2016;218:895-900.

168. Wesch C, Barthel AK, Braun U, Klein R, Paulus M. No microplastics in benthic eelpout (*Zoarces viviparus*): An urgent need for spectroscopic analyses in microplastic detection. Environ Res. 2016;148:36-8.

169. White EM, Clark S, Manire CA, Crawford B, Wang S, Locklin J, et al. Ingested micronizing plastic particle compositions and size distributions within stranded post-hatchling sea turtles. Environ Sci Technol. 2018;52(18):10307-16.

170. Wieczorek AM, Morrison L, Croot PL, Allcock AL, MacLoughlin E, Savard O, et al. Frequency of microplastics in mesopelagic fishes from the northwest atlantic. Front Mar Sci. 2018;5:9.

171. Wieczorek AM, Croot PL, Lombard F, Sheahan JN, Doyle TK. Microplastic ingestion by gelatinous zooplankton may lower efficiency of the biological pump. Environ Sci Technol. 2019;53(9):5387-95.

172. Wilcox C, Puckridge M, Schuyler QA, Townsend K, Hardesty BD. A quantitative analysis linking sea turtle mortality and plastic debris ingestion. Sci Rep. 2018;8:11.

173. Wojcik-Fudalewska D, Normant-Saremba M, Anastacio P. Occurrence of plastic debris in the stomach of the invasive crab eriocheir sinensis. Mar Pollut Bull. 2016;113(1-2):306-11.

174. Woods MN, Stack ME, Fields DM, Shaw SD, Matrai PA. Microplastic fiber uptake, ingestion, and egestion rates in the blue mussel (*Mytilus edulis)*. Mar Pollut Bull. 2018;137:638-45.

175. Wright SL, Rowe D, Thompson RC, Galloway TS. Microplastic ingestion decreases energy reserves in marine worms. Curr Biol. 2013;23(23):R1031-R3.

176. Yu P, Liu ZQ, Wu DL, Chen MH, Lv WW, Zhao YL. Accumulation of polystyrene microplastics in juvenile *Eriocheir sinensis* and oxidative stress effects in the liver. Aquat Toxicol. 2018;200:28-36.

177. Zhang C, Chen XH, Wang JT, Tan LJ. Toxic effects of microplastic on marine microalgae *Skeletonema costatum*: Interactions between microplastic and algae. Environ Pollut. 2017;220:1282-8.

178. Zhu L, Wang H, Chen BJ, Sun XM, Qu KM, Xia B. Microplastic ingestion in deep-sea fish from the South China Sea. Sci Total Environ. 2019;677:493-501.

179. Zhu ZL, Wang SC, Zhao FF, Wang SG, Liu FF, Liu GZ. Joint toxicity of microplastics with triclosan to marine microalgae *Skeletonema costatum*. Environ Pollut. 2019;246:509-17.

180. Abbasi S, Soltani N, Keshavarzi B, Moore F, Turner A, Hassanaghaei M. Microplastics in different tissues of fish and prawn from the Musa Estuary, Persian Gulf. Chemosphere. 2018;205:80-7.

181. Alomar C, Deudero S. Evidence of microplastic ingestion in the shark *Galeus melastomus* Rafinesque, 1810 in the continental shelf off the western Mediterranean Sea. Environ Pollut. 2017;223:223-9.

182. Alomar C, Sureda A, Capo X, Guijarro B, Tejada S, Deudero S. Microplastic ingestion by Mullus surmuletus Linnaeus, 1758 fish and its potential for causing oxidative stress. Environ Res. 2017;159:135-42.

183. Arias AH, Ronda AC, Oliva AL, Marcovecchio JE. Evidence of microplastic ingestion by fish from the Bahia Blanca Estuary in Argentina, South America. Bull Environ Contam Toxicol. 2019;102(6):750-6.

184. Avio CG, Gorbi S, Regoli F. Experimental development of a new protocol for extraction and characterization of microplastics in fish tissues: First observations in commercial species from Adriatic Sea. Mar Environ Res. 2015;111:18-26.

185. Avio CG, Cardelli LR, Gorbi S, Pellegrini D, Regoli F. Microplastics pollution after the removal of the Costa Concordia wreck: First evidences from a biomonitoring case study. Environ Pollut. 2017;227:207-14.

186. Avio CG, Gorbi S, Milan M, Benedetti M, Fattorini D, d'Errico G, et al. Pollutants bioavailability and toxicological risk from microplastics to marine mussels. Environ Pollut. 2015;198:211-22.

187. Azad SMO, Towatana P, Pradit S, Patricia BG, Hue HTT, Jualaong S. First evidence of existence of microplastics in stomach of some commercial fishes in the lower Gulf of Thailand. Appl Ecol Environ Res. 2018;16(6):7345-60.

188. Baalkhuyur FM, Bin Dohaish EJA, Elhalwagy MEA, Alikunhi NM, AlSuwailem AM, Rostad A, et al. Microplastic in the gastrointestinal tract of fishes along the Saudi Arabian Red Sea coast. Mar Pollut Bull. 2018;131:407-15.

189. Bellas J, Martinez-Armental J, Martinez-Camara A, Besada V, Martinez-Gomez C. Ingestion of microplastics by demersal fish from the Spanish Atlantic and Mediterranean coasts. Mar Pollut Bull. 2016;109(1):55-60.

190. Bernardini I, Garibaldi F, Canesi L, Fossi MC, Baini M. First data on plastic ingestion by blue sharks (Prionace glauca) from the Ligurian Sea (North-Western Mediterranean Sea). Mar Pollut Bull. 2018;135:303-10.

191. Besseling E, Foekema EM, Van Franeker JA, Leopold MF, Kuhn S, Rebolledo ELB, et al. Microplastic in a macro filter feeder: Humpback whale *Megaptera novaeangliae*. Mar Pollut Bull. 2015;95(1):248-52.

192. Besseling E, Wegner A, Foekema EM, van den Heuvel-Greve MJ, Koelmans AA. Effects of microplastic on fitness and PCB bioaccumulation by the lugworm *Arenicola marina* (L.). Environ Sci Technol. 2013;47(1):593-600.

193. Birnstiel S, Soares-Gomes A, da Gama BAP. Depuration reduces microplastic content in wild and farmed mussels. Mar Pollut Bull. 2019;140:241-7.

194. Boerger CM, Lattin GL, Moore SL, Moore CJ. Plastic ingestion by planktivorous fishes in the North Pacific Central Gyre. Mar Pollut Bull. 2010;60(12):2275-8.

195. Bonello G, Varrella P, Pane L. First evaluation of microplastic content in benthic filter-feeders of the Gulf of La Spezia (Ligurian Sea). J Aquat Food Prod Technol. 2018;27(3):284-91.

196. Bour A, Haarr A, Keiter S, Hylland K. Environmentally relevant microplastic exposure affects sediment-dwelling bivalves. Environ Pollut. 2018;236:652-60.

197. Brate ILN, Hurley R, Iversen K, Beyer J, Thomas KV, Steindal CC, et al. Mytilus spp. as sentinels for monitoring microplastic pollution in Norwegian coastal waters: A qualitative and quantitative study. Environ Pollut. 2018;243:383-93.

198. Brennecke D, Ferreira EC, Costa TMM, Appel D, da Gama BAP, Lenz M. Ingested microplastics (> 100 µm) are translocated to organs of the tropical fiddler crab *Uca rapax*. Mar Pollut Bull. 2015;96(1-2):491-5.

199. Capolupo M, Franzellitti S, Valbonesi P, Lanzas CS, Fabbri E. Uptake and transcriptional effects of polystyrene microplastics in larval stages of the Mediterranean mussel *Mytilus galloprovincialis*. Environ Pollut. 2018;241:1038-47.

200. Cardozo ALP, Farias EGG, Rodrigues JL, Moteiro IB, Scandolo TM, Dantas DV. Feeding ecology and ingestion of plastic fragments by Priacanthus arenatus: What's the fisheries contribution to the problem? Mar Pollut Bull. 2018;130:19-27.

201. Caron AGM, Thomas CR, Berry KLE, Motti CA, Ariel E, Brodie JE. Ingestion of microplastic debris by green sea turtles (*Chelonia mydas*) in the Great Barrier Reef: Validation of a sequential extraction protocol. Mar Pollut Bull. 2018;127:743-51.

202. Catarino AI, Thompson R, Sanderson W, Henry TB. Development and optimization of a standard method for extraction of microplastics in mussels by enzyme digestion of soft tissues. Environ Toxicol Chem. 2017;36(4):947-51.

203. Catarino AI, Macchia V, Sanderson WG, Thompson RC, Henry TB. Low levels of microplastics (MP) in wild mussels indicate that MP ingestion by humans is minimal compared to exposure via household fibres fallout during a meal. Environ Pollut. 2018;237:675-84.

204. Cheung LTO, Lui CY, Fok L. Microplastic contamination of wild and captive flathead grey mullet (*Mugil cephalus*). Int J Environ Res Public Health. 2018;15(4):11.

205. Cho Y, Shim WJ, Jang M, Han GM, Hong SH. Abundance and characteristics of microplastics in market bivalves from South Korea. Environ Pollut. 2019;245:1107-16.

206. Chua EM, Shimeta J, Nugegoda D, Morrison PD, Clarke BO. Assimilation of polybrominated diphenyl ethers from microplastics by the marine amphipod, *Allorchestes compressa*. Environ Sci Technol. 2014;48(14):8127-34.

207. Cole M, Lindeque P, Fileman E, Halsband C, Galloway TS. The impact of polystyrene microplastics on feeding, function and fecundity in the marine copepod *Calanus helgolandicus*. Environ Sci Technol. 2015;49(2):1130-7.

208. Collard F, Gilbert B, Eppe G, Parmentier E, Das K. Detection of anthropogenic particles in fish stomachs: An isolation method adapted to identification by Raman spectroscopy. Arch Environ Contam Toxicol. 2015;69(3):331-9.

209. Collard F, Gilbert B, Compere P, Eppe G, Das K, Jauniaux T, et al. Microplastics in livers of European anchovies (*Engraulis encrasicolus*, L.). Environ Pollut. 2017;229:1000-5.

210. Collicutt B, Juanes F, Dudas SE. Microplastics in juvenile Chinook salmon and their nearshore environments on the east coast of Vancouver Island. Environ Pollut. 2019;244:135-42.

211. Critchell K, Hoogenboom MO. Effects of microplastic exposure on the body condition and behaviour of planktivorous reef fish (*Acanthochromis polyacanthus*). PLoS One. 2018;13(3):19.

212. Davidson K, Dudas SE. Microplastic ingestion by wild and cultured manila clams (*Venerupis philippinarum*) from Baynes Sound, British Columbia. Arch Environ Contam Toxicol. 2016;71(2):147-56.

213. Desforges JPW, Galbraith M, Ross PS. Ingestion of microplastics by zooplankton in the northeast Pacific Ocean. Arch Environ Contam Toxicol. 2015;69(3):320-30.

214. Devriese LI, van der Meulen MD, Maes T, Bekaert K, Paul-Pont I, Frere L, et al. Microplastic contamination in brown shrimp (*Crangon crangon*, Linnaeus 1758) from coastal waters of the Southern North Sea and Channel area. Mar Pollut Bull. 2015;98(1-2):179-87.

215. Devriese LI, De Witte B, Vethaak AD, Hostens K, Leslie HA. Bioaccumulation of PCBs from microplastics in Norway lobster (*Nephrops norvegicus*): An experimental study. Chemosphere. 2017;186:10-6.

216. Digka N, Tsangaris C, Torre M, Anastasopoulou A, Zeri C. Microplastics in mussels and fish from the Northern Ionian Sea. Mar Pollut Bull. 2018;135:30-40.

217. Donohue MJ, Masura J, Gelatt T, Ream R, Baker JD, Faulhaber K, et al. Evaluating exposure of northern fur seals, callorhinus ursinus, to microplastic pollution through fecal analysis. Mar Pollut Bull. 2019;138:213-21.

218. Farrell P, Nelson K. Trophic level transfer of microplastic: *Mytilus edulis* (L.) to *Carcinus maenas* (L.). Environ Pollut. 2013;177:1-3.

219. Ferreira GVB, Barletta M, Lima ARA, Morley SA, Justino AKS, Costa MF. High intake rates of microplastics in a Western Atlantic predatory fish, and insights of a direct fishery effect. Environ Pollut. 2018;236:706-17.

220. Foekema EM, De Gruijter C, Mergia MT, van Franeker JA, Murk AJ, Koelmans AA. Plastic in North Sea fish. Environ Sci Technol. 2013;47(15):8818-24.

221. Garnier Y, Jacob H, Guerra AS, Bertucci F, Lecchini D. Evaluation of microplastic ingestion by tropical fish from Moorea Island, French Polynesia. Mar Pollut Bull. 2019;140:165-70.

222. Giani D, Baini M, Galli M, Casini S, Fossi MC. Microplastics occurrence in edible fish species (*Mullus barbatus* and *Merluccius merluccius*) collected in three different geographical sub-areas of the Mediterranean Sea

. Mar Pollut Bull. 2019;140:129-37.

223. Goldstein MC, Goodwin DS. Gooseneck barnacles (Lepas spp.) ingest microplastic debris in the North Pacific Subtropical Gyre. PeerJ. 2013;1:17.

224. Goss H, Jaskiel J, Rotjan R. *Thalassia testudinum* as a potential vector for incorporating microplastics into benthic marine food webs. Mar Pollut Bull. 2018;135:1085-9.

225. Gray AD, Weinstein JE. Size- and shape-dependent effects of microplastic particles on adult daggerblade grass shrimp (*Palaemonetes pugio*). Environ Toxicol Chem. 2017;36(11):3074-80.

226. Guven O, Gokdag K, Jovanovic B, Kideys AE. Microplastic litter composition of the Turkish territorial waters of the Mediterranean Sea, and its occurrence in the gastrointestinal tract of fish. Environ Pollut. 2017;223:286-94.

227. Hamer J, Gutow L, Kohler A, Saborowski R. Fate of microplastics in the marine isopod *Idotea emarginata*. Environ Sci Technol. 2014;48(22):13451-8.

228. Hermabessiere L, Paul-Pont I, Cassone AL, Himber C, Receveur J, Jezequel R, et al. Microplastic contamination and pollutant levels in mussels and cockles collected along the channel coasts. Environ Pollut. 2019;250:807-19.

229. Hernandez-Gonzalez A, Saavedra C, Gago J, Covelo P, Santos MB, Pierce GJ. Microplastics in the stomach contents of common dolphin (*Delphinus delphis*) stranded on the Galician coasts (NW Spain, 2005-2010). Mar Pollut Bull. 2018;137:526-32.

230. Herrera A, Stindlova A, Martinez I, Rapp J, Romero-Kutzner V, Samper MD, et al. Microplastic ingestion by Atlantic chub mackerel (*scomber colias*) in the Canary Islands coast. Mar Pollut Bull. 2019;139:127-35.

231. Jabeen K, Su L, Li JN, Yang DQ, Tong CF, Mu JL, et al. Microplastics and mesoplastics in fish from coastal and fresh waters of China. Environ Pollut. 2017;221:141-9.

232. Jeong CB, Won EJ, Kang HM, Lee MC, Hwang DS, Hwang UK, et al. Microplastic size-dependent toxicity, oxidative stress induction, and p-JNK and p-p38 activation in the monogonont rotifer (*Brachionus koreanus*). Environ Sci Technol. 2016;50(16):8849-57.

233. Jovanovic B, Gokdag K, Guven O, Emre Y, Whitley EM, Kideys AE. Virgin microplastics are not causing imminent harm to fish after dietary exposure. Mar Pollut Bull. 2018;130:123-31.

234. Kaposi KL, Mos B, Kelaher BP, Dworjanyn SA. Ingestion of microplastic has limited impact on a marine larva. Environ Sci Technol. 2014;48(3):1638-45.

235. Karami A, Golieskardi A, Ho YB, Larat V, Salamatinia B. Microplastics in eviscerated flesh and excised organs of dried fish. Sci Rep. 2017;7:9.

236. Karlsson TM, Vethaak AD, Almroth BC, Ariese F, van Velzen M, Hassellov M, et al. Screening for microplastics in sediment, water, marine invertebrates and fish: Method development and microplastic accumulation. Mar Pollut Bull. 2017;122(1-2):403-8.

237. Kosore C, Ojwang L, Maghanga J, Kamau J, Kimeli A, Omukoto J, et al. Occurrence and ingestion of microplastics by zooplankton in Kenya's marine environment: First documented evidence. Afr J Mar Sci. 2018;40(3):225-34.

238. Kroon F, Motti C, Talbot S, Sobral P, Puotinen M. A workflow for improving estimates of microplastic contamination in marine waters: A case study from North-Western Australia. Environ Pollut. 2018;238:26-38.

239. Kuhn S, Schaafsma FL, van Werven B, Flores H, Bergmann M, Egelkraut-Holtus M, et al. Plastic ingestion by juvenile Polar cod (*Boreogadus saida*) in the Arctic Ocean. Polar Biol. 2018;41(6):1269-78.

240. Li JN, Yang DQ, Li L, Jabeen K, Shi HH. Microplastics in commercial bivalves from china. Environ Pollut. 2015;207:190-5.

241. Li JN, Qu XY, Su L, Zhang WW, Yang DQ, Kolandhasamy P, et al. Microplastics in mussels along the coastal waters of china. Environ Pollut. 2016;214:177-84.

242. Li JN, Green C, Reynolds A, Shi HH, Rotchell JM. Microplastics in mussels sampled from coastal waters and supermarkets in the United Kingdom. Environ Pollut. 2018;241:35-44.

243. Lourenço PM, Serra-Goncalves C, Ferreira JL, Catry T, Granadeiro JP. Plastic and other microfibers in sediments, macroinvertebrates and shorebirds from three intertidal wetlands of southern Europe and west Africa. Environ Pollut. 2017;231:123-33.

244. Lusher AL, McHugh M, Thompson RC. Occurrence of microplastics in the gastrointestinal tract of pelagic and demersal fish from the English Channel. Mar Pollut Bull. 2013;67(1-2):94-9.

245. Lusher AL, Hernandez-Milian G, O'Brien J, Berrow S, O'Connor I, Officer R. Microplastic and macroplastic ingestion by a deep diving, oceanic cetacean: The True's beaked whale *Mesoplodon mirus*. Environ Pollut. 2015;199:185-91.

246. Lusher AL, O'Donnell C, Officer R, O'Connor I. Microplastic interactions with North Atlantic mesopelagic fish. ICES J Mar Sci. 2016;73(4):1214-25.

247. Magara G, Elia AC, Syberg K, Khan FR. Single contaminant and combined exposures of polyethylene microplastics and fluoranthene: Accumulation and oxidative stress response in the blue mussel, *Mytilus edulis*. J Toxicol Env Health Part A. 2018;81(16):761-73.

248. Mancuso M, Savoca S, Bottari T. First record of microplastics ingestion by European hake *Merluccius merluccius* from the Tyrrhenian Sicilian coast (Central Mediterranean Sea). J Fish Biol. 2019;94(3):517-9.

249. Markic A, Niemand C, Bridson JH, Mazouni-Gaertner N, Gaertner JC, Eriksen M, et al. Double trouble in the south pacific subtropical gyre: Increased plastic ingestion by fish in the oceanic accumulation zone. Mar Pollut Bull. 2018;136:547-64.

250. Mazurais D, Ernande B, Quazuguel P, Severe A, Huelvan C, Madec L, et al. Evaluation of the impact of polyethylene microbeads ingestion in European sea bass (*Dicentrarchus labrax*) larvae. Mar Environ Res. 2015;112:78-85.

251. McGoran AR, Cowie PR, Clark PF, McEvoy JP, Morritt D. Ingestion of plastic by fish: A comparison of Thames Estuary and Firth of Clyde populations. Mar Pollut Bull. 2018;137:12-23.

252. Mohsen M, Wang Q, Zhang LB, Sun L, Lin CG, Yang HS. Microplastic ingestion by the farmed sea cucumber *Apostichopus japonicus* in China. Environ Pollut. 2019;245:1071-8.

253. Morgana S, Ghigliotti L, Estevez-Calvar N, Stifanese R, Wieckzorek A, Doyle T, et al. Microplastics in the arctic: A case study with sub-surface water and fish samples off Northeast Greenland. Environ Pollut. 2018;242:1078-86.

254. Murphy F, Russell M, Ewins C, Quinn B. The uptake of macroplastic & microplastic by demersal & pelagic fish in the northeast atlantic around scotland. Mar Pollut Bull. 2017;122(1-2):353-9.

255. Nadal MA, Alomar C, Deudero S. High levels of microplastic ingestion by the semipelagic fish bogue *Boops boops* (L.) around the Balearic Islands. Environ Pollut. 2016;214:517-23.

256. Naidoo T, Smit AJ, Glassom D. Plastic ingestion by estuarine mullet *Mugil cephalus* (Mugilidae) in an urban harbour, KwaZulu-Natal, South Africa. Afr J Mar Sci. 2016;38(1):145-9.

257. Nelms SE, Galloway TS, Godley BJ, Jarvis DS, Lindeque PK. Investigating microplastic trophic transfer in marine top predators. Environ Pollut. 2018;238:999-1007.

258. Nelms SE, Parry HE, Bennett KA, Galloway TS, Godley BJ, Santillo D, et al. What goes in, must come out: Combining scat-based molecular diet analysis and quantification of ingested microplastics in a marine top predator. Methods Ecol Evol. 2019;10(10):1712-22.

259. Neves D, Sobral P, Ferreira JL, Pereira T. Ingestion of microplastics by commercial fish off the Portuguese coast. Mar Pollut Bull. 2015;101(1):119-26.

260. O'Donovan S, Mestre NC, Abel S, Fonseca TG, Carteny CC, Cormier B, et al. Ecotoxicological effects of chemical contaminants adsorbed to microplastics in the clam *Scrobicularia plana*. Front Mar Sci. 2018;5:15.

261. Ory NC, Gallardo C, Lenz M, Thiel M. Capture, swallowing, and egestion of microplastics by a planktivorous juvenile fish. Environ Pollut. 2018;240:566-73.

262. Paul-Pont I, Lacroix C, Fernandez CG, Hegaret H, Lambert C, Le Goic N, et al. Exposure of marine mussels *Mytilus* spp. to polystyrene microplastics: Toxicity and influence on fluoranthene bioaccumulation. Environ Pollut. 2016;216:724-37.

263. Pegado T, Schmid K, Winemiller KO, Chelazzi D, Cincinelli A, Dei L, et al. First evidence of microplastic ingestion by fishes from the Amazon River estuary. Mar Pollut Bull. 2018;133:814-21.

264. Pellini G, Gomiero A, Fortibuoni T, Ferra C, Grati F, Tassetti AN, et al. Characterization of microplastic litter in the gastrointestinal tract of *Solea solea* from the Adriatic Sea. Environ Pollut. 2018;234:943-52.

265. Perez-Venegas DJ, Seguel M, Paves H, Pulgar J, Urbina M, Ahrendt C, et al. First detection of plastic microfibers in a wild population of South American fur seals (*Arctocephalus australis*) in the Chilean Northern Patagonia. Mar Pollut Bull. 2018;136:50-4.

266. Peters CA, Thomas PA, Rieper KB, Bratton SP. Foraging preferences influence microplastic ingestion by six marine fish species from the Texas Gulf Coast. Mar Pollut Bull. 2017;124(1):82-8.

267. Phuong NN, Poirier L, Pham QT, Lagarde F, Zalouk-Vergnoux A. Factors influencing the microplastic contamination of bivalves from the French Atlantic coast: Location, season and/or mode of life? Mar Pollut Bull. 2018;129(2):664-74.

268. Pittura L, Avio CG, Giuliani ME, d'Errico G, Keiter SH, Cormier B, et al. Microplastics as vehicles of environmental pahs to marine organisms: Combined chemical and physical hazards to the Mediterranean mussels, *Mytilus galloprovincialis*. Front Mar Sci. 2018;5:15.

269. Porter A, Lyons BP, Galloway TS, Lewis C. Role of marine snows in microplastic fate and bioavailability. Environ Sci Technol. 2018;52(12):7111-9.

270. Procter J, Hopkins FE, Fileman ES, Lindeque PK. Smells good enough to eat: Dimethyl sulfide (DMS) enhances copepod ingestion of microplastics. Mar Pollut Bull. 2019;138:1-6.

271. Qu XY, Su L, Li HX, Liang MZ, Shi HH. Assessing the relationship between the abundance and properties of microplastics in water and in mussels. Sci Total Environ. 2018;621:679-86.

272. Romeo T, Pietro B, Peda C, Consoli P, Andaloro F, Fossi MC. First evidence of presence of plastic debris in stomach of large pelagic fish in the mediterranean sea. Mar Pollut Bull. 2015;95(1):358-61.

273. Rummel CD, Loder MGJ, Fricke NF, Lang T, Griebeler EM, Janke M, et al. Plastic ingestion by pelagic and demersal fish from the north sea and baltic sea. Mar Pollut Bull. 2016;102(1):134-41.

274. Setala O, Norkko J, Lehtiniemi M. Feeding type affects microplastic ingestion in a coastal invertebrate community. Mar Pollut Bull. 2016;102(1):95-101.

275. Silva JDB, Barletta M, Lima ARA, Ferreira GVB. Use of resources and microplastic contamination throughout the life cycle of grunts (Haemulidae) in a tropical estuary. Environ Pollut. 2018;242:1010-21.

276. Steer M, Cole M, Thompson RC, Lindeque PK. Microplastic ingestion in fish larvae in the western English Channel. Environ Pollut. 2017;226:250-9.

277. Su L, Deng H, Li BW, Chen QQ, Pettigrove V, Wu CX, et al. The occurrence of microplastic in specific organs in commercially caught fishes from coast and estuary area of east China. J Hazard Mater. 2019;365:716-24.

278. Sun XX, Li QJ, Shi YQ, Zhao YF, Zheng S, Liang JH, et al. Characteristics and retention of microplastics in the digestive tracts of fish from the yellow sea. Environ Pollut. 2019;249:878-85.

279. Tanaka K, Takada H. Microplastic fragments and microbeads in digestive tracts of planktivorous fish from urban coastal waters. Sci Rep. 2016;6:8.

280. Taylor ML, Gwinnett C, Robinson LF, Woodall LC. Plastic microfibre ingestion by deep-sea organisms. Sci Rep. 2016;6:9.

281. Teng J, Wang Q, Ran W, Wu D, Liu YF, Sun S, et al. Microplastic in cultured oysters from different coastal areas of China. Sci Total Environ. 2019;653:1282-92.

282. Vandermeersch G, Van Cauwenberghe L, Janssen CR, Marques A, Granby K, Fait G, et al. A critical view on microplastic quantification in aquatic organisms. Environ Res. 2015;143:46-55.

283. Vendel AL, Bessa F, Alves VEN, Amorim ALA, Patricio J, Palma ART. Widespread microplastic ingestion by fish assemblages in tropical estuaries subjected to anthropogenic pressures. Mar Pollut Bull. 2017;117(1-2):448-55.

284. Vered G, Kaplan A, Avisar D, Shenkar N. Using solitary ascidians to assess microplastic and phthalate plasticizers pollution among marine biota: A case study of the eastern Mediterranean and Red Sea. Mar Pollut Bull. 2019;138:618-25.

285. Vroom RJE, Koelmans AA, Besseling E, Halsband C. Aging of microplastics promotes their ingestion by marine zooplankton. Environ Pollut. 2017;231:987-96.

286. Wagner J, Wang ZM, Ghosal S, Rochman C, Gassel M, Wall S. Novel method for the extraction and identification of microplastics in ocean trawl and fish gut matrices. Anal Methods. 2017;9(9):1479-90.

287. Waite HR, Donnelly MJ, Walters LJ. Quantity and types of microplastics in the organic tissues of the eastern oyster Crassostrea virginica and Atlantic mud crab *Panopeus herbstii* from a Florida estuary. Mar Pollut Bull. 2018;129(1):179-85.

288. Wang J, Wang MX, Ru SG, Liu XS. High levels of microplastic pollution in the sediments and benthic organisms of the South Yellow Sea, China. Sci Total Environ. 2019;651:1661-9.

289. Watts AJR, Lewis C, Goodhead RM, Beckett SJ, Moger J, Tyler CR, et al. Uptake and retention of microplastics by the shore crab *Carcinus maenas*. Environ Sci Technol. 2014;48(15):8823-30.

290. Xiong X, Chen XC, Zhang K, Mei ZG, Hao YJ, Zheng JS, et al. Microplastics in the intestinal tracts of East Asian finless porpoises (*Neophocaena asiaeorientalis sunameri*) from Yellow Sea and Bohai Sea of China. Mar Pollut Bull. 2018;136:55-60.

291. Xu XY, Lee WT, Chan AKY, Lo HS, Shin PKS, Cheung SG. Microplastic ingestion reduces energy intake in the clam *Atactodea striata*. Mar Pollut Bull. 2017;124(2):798-802.

292. Zhao SY, Ward JE, Danley M, Mincer TJ. Field-based evidence for microplastic in marine aggregates and mussels: Implications for trophic transfer. Environ Sci Technol. 2018;52(19):11038-48.

293. Zhu JM, Yu XY, Zhang Q, Li YP, Tan SD, Li D, et al. Cetaceans and microplastics: First report of microplastic ingestion by a coastal delphinid, *Sousa chinensis*. Sci Total Environ. 2019;659:649-54.

294. Duncan EM, Broderick AC, Fuller WJ, Galloway TS, Godfrey MH, Hamann M, et al. Microplastic ingestion ubiquitous in marine turtles. Glob Change Biol. 2019;25(2):744-52.

295. Avio CG, Gorbi S, Regoli F. Plastics and microplastics in the oceans: From emerging pollutants to emerged threat. Mar Environ Res. 2017;128:2-11.

296. Nelms SE, Barnett J, Brownlow A, Davison NJ, Deaville R, Galloway TS, et al. Microplastics in marine mammals stranded around the british coast: Ubiquitous but transitory? Sci Rep. 2019;9:8.

297. Froese R, Pauly D, eds., 2019. FishBase. World Wide Web electronic publication. [www.fishbase.org](https://myjcuedu-my.sharepoint.com/personal/michaela_miller_my_jcu_edu_au/Documents/PhD/002.%20Confirmation%20of%20Candidature/Literature%20Review/PLoS%20One%20Submission/www.fishbase.org), version (12/2019)

298. Palomares M, Pauly D, eds., 2019. SeaLifeBase. World Wide Web electronic publication. [www.sealifebase.org](https://myjcuedu-my.sharepoint.com/personal/michaela_miller_my_jcu_edu_au/Documents/PhD/002.%20Confirmation%20of%20Candidature/Literature%20Review/PLoS%20One%20Submission/www.sealifebase.org), version (12/2019)

299. Setala O, Fleming-Lehtinen V, Lehtiniemi M. Ingestion and transfer of microplastics in the planktonic food web. Environ Pollut. 2014;185:77-83.
